# A phospholipase effector of the type VI secretion system modulates plant reproduction

**DOI:** 10.1128/mbio.01546-25

**Published:** 2025-08-05

**Authors:** Zhi-Min Tan, Jing-Ting Yang, Qing-Jie Xiao, Jing-Tong Su, Zeng-Hang Wang, Yu-Tong Jiang, Jin-Sheng Liu, Tong-Tong Pei, Xiaoye Liang, Ying An, Hong-Wei Xue, Wen-Ming Qin, Wen-Hui Lin, Tao Dong

**Affiliations:** 1 School of Life Sciences and Biotechnology, Joint International Research Laboratory of Metabolic and Developmental Sciences, Shanghai Jiao Tong University 12474, Shanghai, China; 2 Department of Immunology and Microbiology, School of Life Sciences, Guangming Advanced Research Institute, Southern University of Science and Technology 255310, Shenzhen, Guangdong, China; 3 National Facility for Protein Science in Shanghai, Shanghai Advanced Research Institute, Chinese Academy of Sciences 202002, Shanghai, China; 4 Shanghai Collaborative Innovation Center of Agri-Seeds, Joint Center for Single Cell Biology, School of Agriculture and Biology, Shanghai Jiao Tong University 117750, Shanghai, China; 5 College of Agriculture, South China Agriculture University 162683, Guangzhou, Guangdong, China; National University of Singapore, Singapore, Singapore

**Keywords:** *Xanthomonas oryzae *pv. *oryzae*, ovule initiation, T6SS, phosphatidylserine

## Abstract

Phytobacteria play diverse roles in plant biology, ranging from promoting health to causing diseases that threaten global food security and the economy. In contrast to the extensive studies of phytopathogens targeting leaves and roots, their impact on plant reproductive processes has been largely overlooked. Here, we demonstrate that a bacterial effector TleB of the type VI secretion system in *Xanthomonas oryzae* can modulate seed production of *Arabidopsis thaliana*. Using biochemical, structural, and physiological analyses, we determined TleB as a phospholipase that mediates interspecies microbial competition in *X. oryzae*. Additionally, TleB plays a key role in the infection of inflorescences by *X. oryzae*, which leads to significantly reduced seed production. Lipidomic and biochemical assays show that TleB binds to a number of anionic phospholipids that are key signaling molecules. A fluorescence reporter for auxin distribution showed TleB-mediated diminished signals *in planta*. Additionally, transgenic plants expressing TleB exhibited significantly altered seed counts. These findings introduce a novel paradigm in which phytopathogens can affect plant reproduction in a traditionally non-susceptible host, prompting a reevaluation of diverse phytobacteria-host interactions in reproductive processes and offering new insights into plant health and crop protection.

**IMPORTANCE:**

Phytobacteria are typically identified as pathogens based on visible effects on leaves and roots; those lacking such phenotypes are often considered nonpathogenic. Similarly, plant hosts that show no phenotypic changes are considered nonhosts and, thus, less studied. Our research challenges this classification by highlighting that bacteria-plant interactions on inflorescences, though less apparent and more delayed, can cause profound impacts on seed production. This discovery not only shifts the focus from the more commonly studied vegetative and root infections to the reproductive aspects of plant-pathogen interactions but also necessitates a reevaluation of host-pathogen dynamics with an emphasis on long-term effects such as seed production.

## INTRODUCTION

Phytobacteria are commonly found on almost all exposed surfaces of plants and carry out complex functions ranging from causing plant diseases to promoting nutrient acquisition and plant growth (1–3). These functions are mediated by a variety of mechanisms, among which protein secretion systems play a crucial role in phytobacteria-plant interactions (4). Of the six major secretion systems in gram-negative bacteria, the type III secretion system (T3SS) has been extensively studied as a critical virulence factor in many phytopathogens (4–7). Although the type VI secretion system (T6SS) has also been implicated in nutrient acquisition (8), interbacterial competition (9–12), and virulence against the host (11, 13–17), how the T6SS modulates plant hosts is much less understood.

The T6SS is present in approximately 25% of gram-negative bacteria, especially within the classes α-, β-, and γ-proteobacteria (18). The T6SS structure features a membrane complex (TssJ/L/M), a baseplate (TssE/F/G/K), and an Hcp inner tube encased by a contractile outer sheath (TssB/C) (19–22). Upon sheath contraction, the Hcp tube and a cone-shaped VgrG-PAAR (PAAR, Pro-Ala-Ala-Arg repeats containing protein, T6SS structure protein) tip complex, sometimes carrying their interacting effectors, are ejected into target cells (19–22). Each antibacterial effector is accompanied by a corresponding immunity protein, which neutralizes effector toxicity and provides self-protection (19–22). Some effectors also require cognate chaperones for stability and loading onto the secretion apparatus (23). In addition to their antibacterial activities, T6SS effectors exhibit great diversity and possess potent killing capabilities against amoebae, yeast, and other eukaryotic cells (24–26).

*Xanthomonas* is a widespread plant-associated genus, comprising over 35 pathogenic species that cause significant economic losses in over 400 economically important crops, such as rice, wheat, citrus, tomato, pepper, cabbage, cassava, banana, and bean crops (27, 28). *Xanthomonas oryzae* causes bacterial blight in rice, one of the most devastating rice diseases worldwide. Yield losses can range from 10% to 30%, and in severe cases, may result in complete crop failure (29). The PXO99A strain possesses several virulence factors, with the T3SS playing a central role in rice infection (30). PXO99A also encodes two distinct sets of T6SS, designated T6SS-1 and T6SS-2. The function of T6SS-1 remains unknown. In contrast, the role of T6SS-2 has been inconsistently reported: one study suggested that T6SS-2 contributes to virulence in rice but lacks antibacterial activity (31), whereas another study concluded that it mediates interbacterial competition but is dispensable for virulence (32). In the closely related strain *X. oryzae* pv*. oryzicola* GX01, neither T6SS-1 nor T6SS-2 is required for virulence in rice; T6SS-1 appears to be inactive and T6SS-2 displays antibacterial activity (33). These discrepancies may reflect differences in strain backgrounds or experimental conditions. Notably, PXO99A does not cause visible disease symptoms in *Arabidopsis thaliana* due to nonhost resistance (34).

In higher plants, there are usually three developmental phases: a juvenile vegetative phase, an adult vegetative phase, and a reproductive phase (35). The reproductive phase is marked by the development of inflorescence, the flower-bearing structure. Conventional identification of plant pathogens typically focuses on their visible effects on leaves, roots, or overall plant health. However, subtler and long-term developmental effects on flowers, seeds, and reproductive processes are often overlooked. Consequently, known virulence mechanisms are predominantly associated with those obvious phenotypic traits, as exemplified by the spotlighted T3SS for its pivotal role in virulence (28, 30). Here, focusing on the T6SS-2 and its effectors in *X. oryzae* strain PXO99A as a model, we demonstrate a novel pathway by which phytobacteria may influence plant reproduction, highlighting the need to explore plant-pathogen interactions beyond the traditional acute diseases.

## RESULTS

### TleA and TleB are two duplicated phospholipase effectors of the T6SS-2

**Fig 1:**
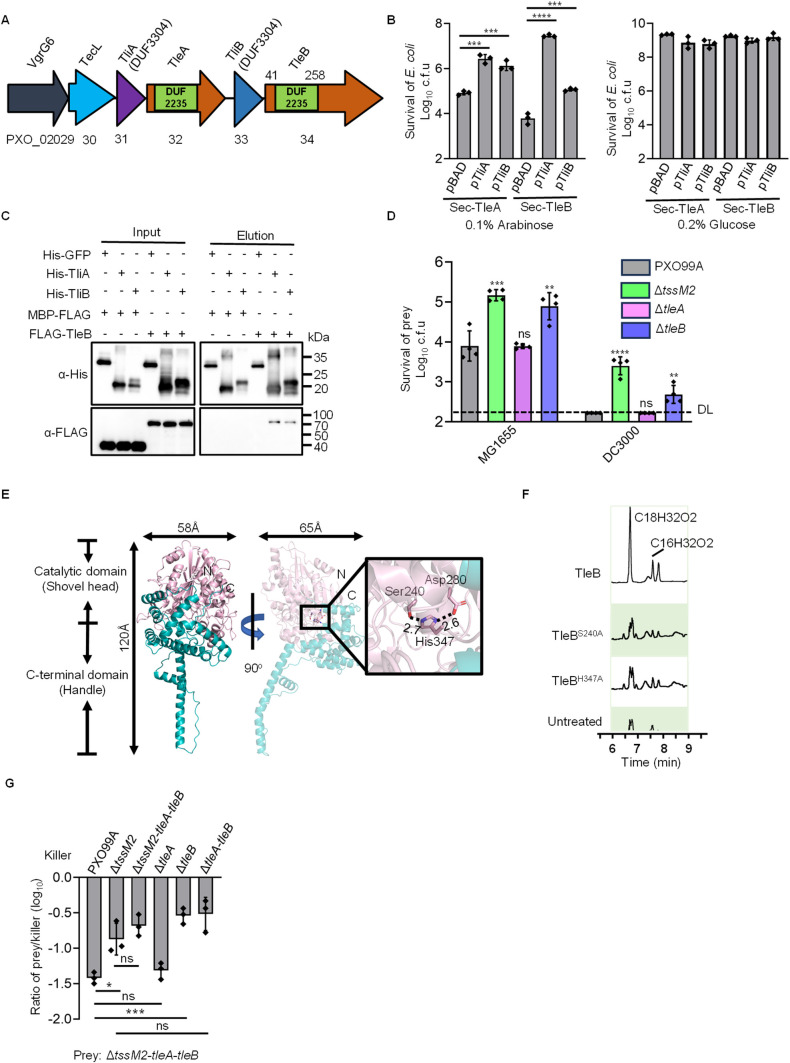
Characterization of T6SS phospholipase effector TleB. (**A**) Organization of the PXO_02029-PXO_02034 operon. The alpha/beta hydrolase domain DUF2235 of TleA and TleB was predicted using HMMER. (**B**) Toxicity assay of TleA and TleB. Results show the survival of *Escherichia coli* that express TleA or TleB with an empty vector (pBAD) or a vector carrying the immunity gene *tliA* or *tliB*. Sec, periplasmic secretion signal. (**C**) Interaction of TleB with TliA or TliB. Pull-down analysis was performed using His-GFP (control), His-TliA or TliB, MBP-FLAG (control), and FLAG-TleB. A similar pull-down assay was performed for the interaction of TleA with immunity proteins and shown in Fig. S2H. (**D**) Competition assay of PXO99A, T6SS-2-null mutant (Δ*tssM2*), or effector-immunity deletion mutants (Δ*tleA* and Δ*tleB*) against *E. coli* MG1655 (MG1655, co-cultured for 6 hours) or *Pseudomonas syringae* pv. *tomato* DC3000 (DC3000, co-cultured for 3 hours) in *Nicotiana benthamiana*, respectively. (**E**) Crystal structure of TleB, color-coded from pink (N-terminus) to green (C-terminus). The catalytic triad residues Ser240-Asp280-His347 of TleB are highlighted on the right. (**F**) UPLC chromatograms of digested lecithin products by purified TleB, TleB^S240A^, and TleB^H347A^. Peaks were identified using mass spectrometry. (**G**) Competition assay of PXO99A, T6SS-2-null mutant (Δ*tssM2*), effector-immunity deletion mutants (Δ*tleA* and Δ*tleB*), effector-immunity deletion mutant (Δ*tleA-tleB*), and Δ*tssM2-tleA-tleB* against Δ*tssM2-tleA-tleB*. Error bars indicate the mean ± standard deviation of three biological replicates, and statistical significance was calculated using a two-tailed Student’s *t*-test. *P < 0.05, **P < 0.01, ***P < 0.001, and ****P < 0.0001. DL, detection limit.

The T6SS functions as a membrane-anchored, contractile apparatus that delivers a wide array of toxic effectors into adjacent prokaryotic or eukaryotic cells (19, 36–40). Leveraging a chaperone-based effector prediction approach (38), we identified a gene cluster within PXO99A, specifically PXO_02029-PXO_02034 (Fig. 1A). This cluster encodes a VgrG protein (PXO_02029), a DUF4123 chaperone (PXO_02030), two DUF3304 proteins (PXO_02031 and PXO_02033), and two proteins with DUF2235 domains (PXO_02032 and PXO_02034). The DUF2235 domain is linked to proteins containing Tle1 phospholipase domains (Fig. S1). Notably, the two DUF2235 proteins exhibit almost identical sequences at the N-terminus and share a common C-terminal domain with other DUF2235 proteins, indicating a recent gene duplication event (Fig. S2A). The operon structure implies a set of effector-immunity protein paralogs. We propose the following nomenclature for clarity: VgrG6 (PXO_02029, Vgr, valine-glycine repeat family proteins, T6SS structure protein), TecL (PXO_02030, T6SS effector chaperone), TliA (PXO_02031, T6SS lipase immunity), TleA (PXO_02032), TliB (PXO_02033, T6SS lipase immunity), and TleB (PXO_02034).

To investigate the toxicity of TleA and TleB, we employed an arabinose-inducible pBAD vector for their expression in *Escherichia coli*, both with and without a Sec-dependent periplasmic signal sequence. Periplasmic expression of TleA and TleB resulted in significant toxicity, which could be mitigated by co-expression with TliA or TliB (Fig. 1B; Fig. S2B). Conversely, cytoplasmic expression of these proteins did not induce toxicity (Fig. S2C and D). Both TliA and TliB possess high-confidence Sec-dependent signal peptides, indicative of periplasmic targeting (Fig. S2E). Consistently, fluorescence microscopy of *E. coli* expressing TliA-sfGFP or TliB-sfGFP revealed signals at the cell periphery, presumably in the periplasmic space (Fig. S2G). Additionally, expressing only the N-terminal DUF2235 domain of TleB in the periplasm was toxic, a condition reversible by co-expression with TliA or TliB (Fig. S2F). Furthermore, pull-down assays indicate interactions between effector-immunity pairs (Fig. 1C; Fig. S2H). These findings suggest the specific targeting and toxic mechanisms of TleA and TleB in the periplasmic space.

Given the observed cross-protection of TliA and TliB, we deleted *tliA-tleA-tliB-tleB* genes, resulting in the Δ*tleA-tleB* mutant. This mutant demonstrated significantly reduced survival in a competition assay against wild type, Δ*tssM1* mutant (T6SS-1 defective), or Δ*tleA* mutant, compared to the Δ*tssM2* (T6SS-2 defective) mutant and the Δ*tleB* mutant (Fig. S2I), suggesting this is a T6SS2-dependent effector cluster. Additionally, when we introduced the *tssM2* deletion to the Δ*tleA-tleB* mutant and compared its competition ability with the Δ*tssM2* and the Δ*tleA-tleB* mutant, no significant difference was observed (Fig. 1G), further supporting its T6SS-2 dependency. Additionally, competition assays were conducted on the leaves of the *Nicotiana benthamiana* against *E. coli* MG1655 and *Pseudomonas syringae* pv. *tomato* DC3000, a significant phyllosphere pathogen. The assays demonstrated T6SS-dependent suppression of both *E. coli* and *P. syringae*, with deletion of *tleB* impairing the suppression (Fig. 1D). Complementation of the immunity protein TliB in prey strains MG1655 and DC3000 significantly increased their survival in competition assays (Fig. S2J and K). Furthermore, we complemented the mutants and performed competition assays. The results showed that the *tssM2* deletion could be functionally restored by a plasmid-borne copy, while ectopic *tleA* expression further enhanced the killing activity of the already active *tleA* mutant. In contrast, *tleB* complementation failed, despite detectable TleB expression (Fig. S2L and M).

To exclude a compromised T6SS as the cause of the diminished competition of the Δ*tleA-tleB* mutant, we evaluated Hcp secretion, a T6SS hallmark. Western blot analysis showed that Hcp secretion was unaffected by the deletion of *tleA* or *tleB* (Fig. S2N). These findings collectively indicate that TleA and TleB are T6SS-2 effectors contributing to the competitive fitness of PXO99A.

### TleB is a shovel-like effector with dual PLA1 and PLA2 phospholipase activities

Considering the high sequence similarity of TleA and TleB, we focused on characterizing the function of TleB. The crystal structure of TleB was solved by the molecular replacement method using the coordinates of the phospholipase catalytic domain of Tle1 (PDB code: 4O5P) as a search model and refined to 1.9 Å resolution (Table S1). The structure adopts a shovel-like shape, with overall dimensions of ~120 × 58 × 65 Å (Fig. 1E). Structural analysis reveals two primary domains: a phospholipase catalytic module and a C-terminal domain (Fig. 1E). The catalytic module, characterized by about 40% sequence conservation and a low 1.3 Å root-mean-square deviation, adopts a compact α/β mixed hydrolase fold comprising one central β-sheet of 8 β-strands surrounded by 14 α-helices and 2 3_10_-helices (Fig. 1E). Consistent with other phospholipases (such as Tle1^PA^ and Tle1^EAEC^) (41, 42), TleB also harbors a highly conserved catalytic triad (Ser240, Asp280, and His347) (Fig. 1E). Conversely, the C-terminal domain with an unknown function diverges more significantly in structure, comprising helices and loops that form a shovel-like shape with the “shovelhead” over the catalytic pocket and “handle” formed by long helices (Fig. 1E). This crystallographic analysis delineates TleB’s domain architecture, highlighting a conserved phospholipase module and a distinct C-terminal domain.

To determine the enzymatic activity of TleB, we employed liquid chromatography-mass spectrometry (LC-MS) analysis using lecithin—a phosphoglyceride mixture—as the substrate. The phospholipase A (PLA) superfamily encompasses PLA1 and PLA2 subtypes. PLA1 cleaves the acyl ester at the sn-1 position and PLA2 at the sn-2 position (43). Two TleB mutants, TleB^S240A^ and TleB^H347A^, were constructed by substituting alanine for the predicted catalytic residues S240 and H347, respectively. Our analysis revealed that while wild-type TleB efficiently hydrolyzed lecithin, its mutants failed to do so, indicative of abolished activity (Fig. 1F). The detection of enzymatic products (C18H32O2 and C16H32O2) highlights TleB with dual PLA1 and PLA2 phospholipase activities (Fig. S3A). These data further indicate TleB as a phospholipase effector.

### TleB modulates plant-cell lipid compositions

**Fig 2:**
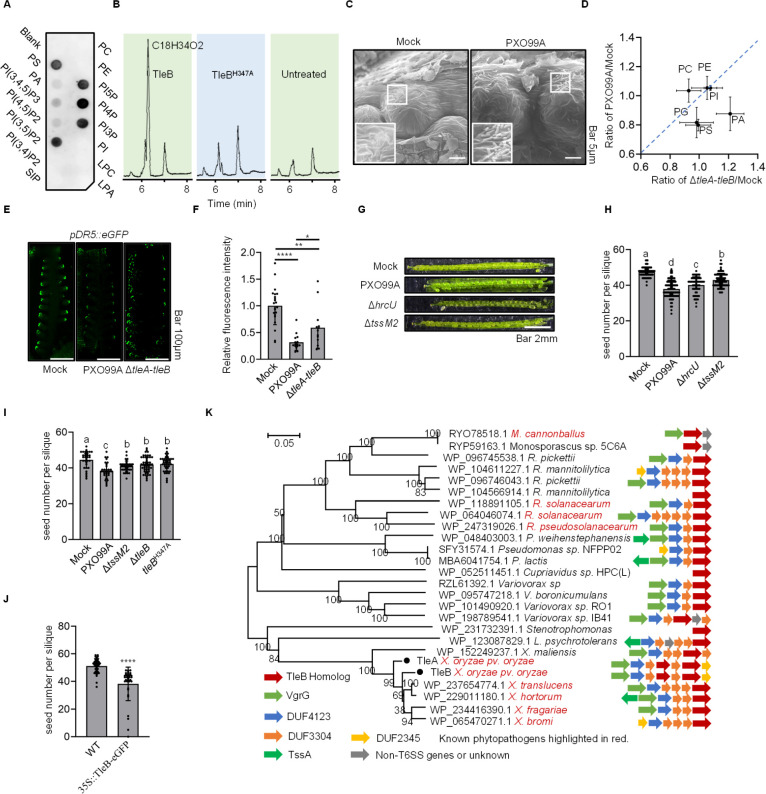
TleB inhibits seed development in *A. thaliana*. (**A**) Fat-western immunoblot analysis of TleB. Each dot represents a different phospholipid type: phosphatidylcholine (PC), phosphatidylethanolamine (PE), PS, PA, lysophosphatidic acid (LPA), lysophosphatidylcholine (LPC), sphingosine 1-phosphate (S1P), phosphatidylinositol (PI), PI3P, PI4P, PI5P, PI (3,4)P2 (PI 3,4-bisphosphate), PI (3,5)P2, PI (4,5)P2, and phosphatidylinositol 3,4,5-trisphosphate [PI (3,4,5)P3]. (**B**) UPLC chromatograms of digested PS products by TleB. Products were detected using mass spectrometry. (**C**) Scanning electron micrographs showing attached bacterial cells on the ovule surface. Mock, 10 mM MgCl_2_. (**D**) Lipidomic analysis of PS content in *A. thaliana* inflorescences. Phospholipids were profiled using mass spectrometry. Mock, 10 mM MgCl_2_. PG, phosphatidylglycerol. (**E**) and (**F**) Confocal microscopy of *pDR5::eGFP* reporter expression in *A. thaliana* inflorescences treated with mock (10 mM MgCl_2_), PXO99A, and Δ*tleA-tleB* mutant. (**G**) Images of siliques from *A. thaliana* treated with the mock (10 mM MgCl_2_), PXO99A, ΔT3SS (Δ*hrcU*), and ΔT6SS-2 (Δ*tssM2*) mutants. (**H**) Quantification of seed numbers per silique in *A. thaliana* treated with the mock (10 mM MgCl_2_), PXO99A, ΔT3SS (Δ*hrcU*), and ΔT6SS-2 (Δ*tssM2*) mutants. (**I**) Seed numbers per silique in *A. thaliana* treated with the mock (10 mM MgCl_2_), PXO99A, Δ*tssM2*, Δ*tleB*, and *tleB*^H347A^ (catalytically inactive chromosomal mutation). (**J**) Seed numbers in transgenic *A. thaliana* expressing TleB (Seed numbers indicate normal, fully developed seeds, excluding aborted ones). (**K**) Phylogenetic analysis of TleB homologs using MEGA7.0 with the Neighbor-Joining method and 1,000 bootstrap values. Using TleB as the query and BlastP, we retrieved the top 24 representative sequences for analysis. The operon structures are indicated, with T6SS-conserved proteins marked in color. Protein sequences are provided in Data S2. Total seeds, including abortive ones, are counted. For panels H and I, the letters displayed above the bar graphs indicate statistically significant differences. Identical letters denote no significant difference between the corresponding groups, while different letters signify a statistically significant difference between the groups. Data are presented as mean ± SD. Statistical significance was determined using one-way ANOVA for panel **H** and two-tailed Student’s *t*-test for panels **F**, **I**, and **J** (*P < 0.05, **P < 0.01, and ****P < 0.0001).

Because PXO99A is a phytopathogen and TleB exhibits phospholipase activity, we next tested whether TleB is involved in interaction with the host by assessing its interaction with various plant-associated lipids through a protein-lipid overlay assay. TleB demonstrated strong affinity for phosphatidylserine (PS), alongside phosphoinositides PI 3-monophosphate (PI3P), PI4P, PI5P, and PI (3, 5)P2, while exhibiting weaker interactions with other lipids like phosphatidic acid (PA) and PI (3,4,5)P3 and PI (4, 5)P2 (Fig. 2A). PS is an important and abundant anionic lipid that plays important and diverse functions in plant physiology (44), while the phosphoinositides identified are key regulators of membrane identity and trafficking in eukaryotic cells (45). Given the critical role of PS in plants, we next tested PS hydrolysis *in vitro* using purified TleB and a catalytically inactive mutant, TleB^H347A^. LC-MS analysis confirmed effective hydrolysis of PS by TleB (Fig. 2B; Fig. S3B).

When testing interaction between PXO99A and host cells, we serendipitously detected the adherence of PXO99A to ovule surfaces using scanning electron microscopy (Fig. 2C). Lipidomic profiling of *A. thaliana* inflorescences revealed a significant reduction in PS and PA levels following treatment with the wild-type strain compared to MgCl_2_ controls. In contrast, treatment with the Δ*tleA-tleB* mutant did not affect PS levels but was associated with a modest increase in PA (Fig. 2D), the significance of which remains to be clarified. As TleA and TleB share 90% sequence identity and likely perform redundant functions, we used the double mutant to ensure a robust readout of phospholipase-dependent effects.

Given the critical signaling role of PS and the other TleB-interacting anionic lipids in plants (44), we examined auxin signaling by monitoring the expression of the auxin-responsive reporter pDR5::eGFP in *A. thaliana* inflorescences. Using confocal fluorescence microscopy, we observed significantly reduced reporter signals in inflorescences treated with the wild-type strain compared to those treated with the Δ*tleA-tleB* mutant, suggesting auxin signaling is attenuated (Fig. 2E and F).

### TleB reduces the seed numbers

Although *A. thaliana* is known to display nonhost resistance to PXO99A (34), we noticed that infection of PXO99A in *A. thaliana* inflorescences significantly reduced seed numbers relative to the mock control (Fig. 2G and H). This reduction appears to be more alleviated by inactivating the T6SS-2 than the T3SS (Fig. 2G and H). The Δ*tleB* mutant, as well as the catalytically inactive *tleB* mutants, displayed seed numbers comparable to the Δ*tssM2* group, suggesting that the impact on seed formation is predominantly mediated through the phospholipase activity of TleB (Fig. 2I).

To further elucidate the role of TleB, a transgenic *A. thaliana* plant expressing TleB-eGFP under the constitutive 35S promoter was generated. Western blot analysis confirmed TleB-eGFP expression in both inflorescences and leaves (Fig. S3D). Fluorescence imaging analysis of the root revealed cytoplasmic expression of TleB-eGFP (Fig. S3E). These transgenic plants exhibited a significant reduction in the number of seeds per silique compared to control plants (Fig. 2J), despite showing no observable defects in overall plant health (Fig. S3C). These results collectively indicate that TleB can reduce seed formation in *A. thaliana*.

### TleB-family effectors exist in diverse species

Through blastp analysis aimed at mapping the distribution of TleB homologs, we identified and analyzed the top 24 non-redundant representative proteins. This analysis revealed that TleB homologs are not confined to the genus *Xanthomonas* but are prevalent across a broad spectrum of plant-associated bacteria, including but not limited to genera such as *Monosporascus, Ralstonia, Pseudomonas, Cupriavidus, Variovorax,* and *Stenotrophomonas* (Fig. 2K). This wide distribution highlights the potential ecological importance of TleB-family effectors in interactions between a diverse set of bacteria and plant hosts.

## DISCUSSION

**Fig 3:**
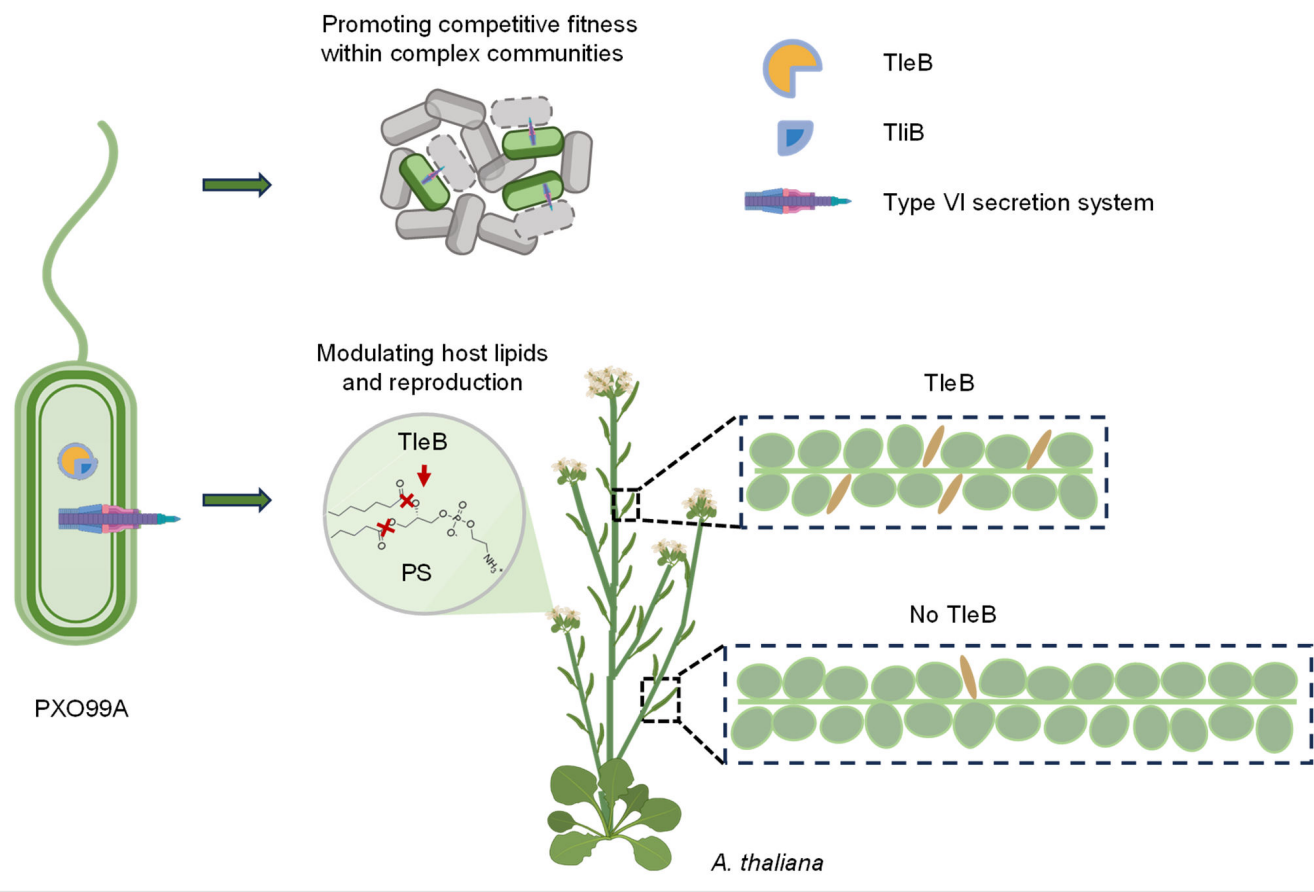
Schematic model of the TleB-mediated host-microbe interactions. The T6SS effector TleB in *X. oryzae* PXO99A exhibits dual PLA1 and PLA2 phospholipase activities, which aid in microbial competition and host-microbe interaction. By targeting and neutralizing competing microbes, TleB promotes the competitive fitness of PXO99A and successful colonization. By hydrolyzing key anionic phospholipids in the host plant, TleB reduces seed production in *A. thaliana*. The widespread presence of TleB homologs in various T6SS-encoding phytobacteria suggests a common mechanism affecting plant health. These findings highlight a previously unrecognized impact of bacterial effectors on flowering organs and relatively long-term phenotypic processes in plant-microbe interactions, providing new insights into the complexities of plant-pathogen dynamics and potential strategies for crop protection.

Here, we demonstrate that the T6SS-secreted phospholipase TleB acts as a cross-kingdom dual-functional effector, mediating both interbacterial competition and host manipulation (Fig. 3). TleB disturbs *Arabidopsis* inflorescence development and seed production through lipid degradation. These findings represent a pivotal advance in understanding plant-pathogen dynamics, as the flowering organ is less frequently regarded as an infection entry point compared to leaves and roots. Factors such as insect feeding, frost damage, and irrigation can facilitate direct pathogen contact with flowers. This subsequent impact on seed quantity manifests as a long-term effect, complicating the detection, tracing, and mitigation of its causes. Therefore, this study expands our knowledge of plant-pathogen interactions to include not only the traditional diseases affecting leaves, roots, and whole plants but also the subtle, yet crucial, alterations in plant reproductive processes. Our findings could aid future investigations in establishing a microbial cause for crop yield reduction.

*Xanthomonas* species exhibit a broad host range, capable of colonizing hundreds of plant hosts, including rice, tomato, and lettuce (46). This ecological versatility creates numerous opportunities for interactions with diverse bacterial species. For example, *P. syringae* is a well-characterized phytopathogen that infects tomato and other crops (47). Similarly, pathogenic *E. coli* strains (including O157:H7 and O104:H4) frequently contaminate fresh produce, such as lettuce, spinach, and sprouts, posing global public health risks (48, 49). These overlapping ecological niches suggest that antibacterial effectors like TleB and its homologs may provide *Xanthomonas* with a competitive advantage, potentially enhancing its survival and transmission in polymicrobial plant environments.

Although there is some evidence of T6SS in phytobacteria contributing to host virulence—for instance, inactivation of *Burkholderia glumae* BGR1’s T6SS reduces its rice infectivity (17), T6SS-1 is crucial for *Pantoea ananatis* LMG 2665T’s onion pathogenesis (11), and the T6SS effector TseG is essential for the pathogenicity of *Pantoea ananatis* DZ-12 on maize plants (50)—the mechanisms behind these processes require further elucidation. Our study reveals dual roles of the phospholipase effectors of the T6SS in PXO99A, one for bacterial competition and the other for seed development. The phospholipase A superfamily, encompassing PLA1 and PLA2 subtypes, plays a pivotal role in hydrolyzing membrane glycerophospholipids at distinct positions (43). In plants, PLA-derived phospholipid products act as crucial signaling molecules, influencing various cellular functions like cell elongation, geotropism, and defense signaling (51, 52). PLA2, in particular, participates in auxin signal transduction (53). Additionally, considering the diverse functions of PS in plants, the decrease in PS is likely causing pleiotropic effects. Platre et al. (54) mechanistically demonstrated that PS is required for the clustering of ROP6, a small guanosine triphosphatase (GTPase), at the plasma membrane in response to signals from the plant hormone auxin. Auxin plays a major role in diverse processes in plants, including seed development (55). Our observed PS reduction would be predicted to disrupt this PS-ROP6-Auxin signaling cascade. We verified this prediction using the auxin-responsive reporter DR5:eGFP, whose *in planta* expression was altered by the wild type but not the lipase mutants (Fig. 2E and F). Considering the importance of PS and other TleB phospholipid substrates and the widespread occurrence of TleB in phytobacterial species, our findings would have a broad impact on understanding not only plant physiology but also plant-microbe interactions.

The potent antibacterial properties of the T6SS may be exploited for biocontrol and therapeutic uses. Yet, our research points to a potential risk of its direct application, notably the reduction in seed numbers. Our study highlights the importance of studying effector functions. Interestingly, TleB expression is not overtly harmful to plants, which appear capable of tolerating such effects without manifesting significant damage (Fig. S3C). Notably, despite multiple experimental approaches, we were unable to detect TleB secretion. This may reflect a requirement for precise stoichiometric assembly of TleB with its cognate VgrG and chaperone, or alternatively, may suggest that TleB secretion requires *cis*-expression from its native genetic context (Fig. S2L and M). In a related study, we elucidate the mechanism of plant cellular response to TleB, identifying a molecular switch that governs the balance between resistance and development (56). Collectively, these findings highlight the importance of defining virulence features and phytopathogens by including not only phenotypic changes in roots and leaves but also long-term effects such as plant development and seed production.

## MATERIALS AND METHODS

### Strains and growth conditions

All strains, plasmids, and primers used in this study are listed in Tables S2 and S3. *E. coli* was cultured in LB medium ([wt/vol] 1% tryptone, 0.5% yeast extract, and 0.5% NaCl) under aerobic conditions at 37°C. PXO99A was cultured in NB medium ([wt/vol] 0.3% beef extract, 0.1% yeast extract, 0.5% polypeptone, and 0.5% sucrose) or NBN medium ([wt/vol] 0.3% beef extract, 0.1% yeast extract, and 0.5% polypeptone) or NBS medium ([wt/vol] 0.3% beef extract, 0.1% yeast extract, 0.5% polypeptone, and 10% sucrose) under aerobic conditions at 28°C. The concentrations of antibiotics and inducers used were as follows: ampicillin (100 µg/mL), kanamycin (50 µg/mL), gentamicin (20 µg/mL), chloramphenicol (25 µg/mL), arabinose (0.1%, wt/vol), IPTG (0.1 or 1 mM as indicated), and glucose (0.2%, wt/vol). PS was obtained from Avanti (CAS: 90693-88-2). IAA was obtained from ANPEL-TRACE (CAS: 87-51-4). Mutants were constructed based on the suicide plasmid pK18mobSacB using homologous double exchange, and all constructs were verified through sequencing.

### Western blotting

The proteins were separated through SDS-PAGE and then transferred onto a polyvinylidene difluoride membrane (Bio-Rad). Subsequently, the membrane was blocked using 5% non-fat milk in TBST buffer (50 mM Tris, 150 mM NaCl, and 0.1% Tween-20, pH 7.6) at room temperature for 1 hour. The membrane was then exposed to primary antibodies, followed by secondary HRP-conjugated antibodies in TBST containing 1% milk at room temperature for 1 hour. Signals were visualized using the Clarity ECL solution (Bio-Rad).

The monoclonal antibody to RpoB (Product #663905) was sourced from BioLegend, the antibody to eGFP (Product #SB-AB0005) from ShareBio, and the antibody to ACTIN (Product #AC009) from ABClonal. The polyclonal antibody to Hcp2 was custom-made by Shanghai Youlong Biotech. Monoclonal antibodies to epitope tags (Product #SLAB2803 [His] and Product #SLAB0102 [FLAG]) were from Smart-Lifesciences. Secondary antibodies were sourced from ZSGB-Bio (Product #ZB-2305 [mouse] and #ZB-2301 [rabbit]). The Hcp antibody was diluted 1:10,000, while the other antibodies were diluted 1:20,000.

### Bacteria competition assay

Bacteria were cultured in NB liquid medium at 28°C and grown to an OD_600_ of approximately 1. The cells were then harvested by centrifugation and resuspended. The ratio of killer to prey cells was adjusted to 10:1, and the mixture was spotted onto NB plates at 28°C (MG1655: 3 hours and DC3000: 2 hours). After incubation, the survival of killer and prey cells was assessed by counting the colony-forming units (CFUs) on NB or LB medium supplemented with selective antibiotics, using a 10-fold serial dilution.

For the competition assay *in planta*, we used 4-week-old *N. benthamiana* plants. The killer strain (PXO99A, Km^R^) and prey strains (*E. coli* MG1655 and *P. syringae* pv. *tomato* DC3000 Gm^R^) were mixed in a 1:1 ratio, with the killer strain at OD_600_ = 10 and the prey strain at OD_600_ = 1. Bacterial cells were injected into the back of tobacco leaves, and the plants were incubated in the plant greenhouse for 3 hours (DC3000) and 6 hours (MG1655). After incubation, bacterial cells were collected from the infected area using a blunt-tipped 1 mL pipette, quickly cooled, and ground with liquid nitrogen. The samples were then resuspended in liquid NB medium and subjected to 10-fold serial dilution, resistance plate screening, and counting statistics.

The mean log_10_ CFU of recovered cells was calculated and plotted, with error bars representing the mean ± standard deviation of three biological replicates. Statistical analysis was performed using a two-tailed Student’s *t*-test to determine the *P*-values.

### Protein pull-down assay

The genes of interest were tagged with His or FLAG and cloned into the pBBR1MCS-2 and pET vectors for expression. Cells were cultured in LB or NB medium with appropriate antibiotics until reaching an optical density (OD)_600_ of 0.6. Induction was carried out by adding 0.1% arabinose at 30°C for 3 hours or at 28°C for five hours, or by adding 1 mM IPTG at 20°C overnight. After induction, the cells were collected by centrifugation, and the pellet was resuspended in 1 mL of lysis buffer (20 mM Tris, pH 8.0, 500 mM NaCl, and 50 mM imidazole, supplemented with protease inhibitor from Thermo Scientific). The cells were then lysed, and cell debris was removed by centrifugation. The resulting supernatant was mixed with Ni-NTA beads (Smart Lifesciences). Next, the protein-bound beads were washed four times with washing buffer (20 mM Tris, pH 8.0, 500 mM NaCl, and 50 mM imidazole) to remove any non-specifically bound contaminants. Finally, the proteins of interest were eluted using elution buffer (20 mM Tris, pH 8.0, 500 mM NaCl, and 500 mM imidazole). The eluted samples were then analyzed by Western blotting.

### Protein toxicity assay

Cells harboring different pBAD vectors were cultured overnight at 37°C in LB medium supplemented with 0.2% glucose. Cultures were then diluted into fresh medium and grown to an OD_600_ of ~1, followed by the addition of either 0.1% arabinose or 0.2% glucose. After incubation at 37°C for 5 hours, cultures were serially diluted 10-fold and plated onto LB agar containing either 0.1% arabinose or 0.2% glucose. Each experiment was performed in triplicate.

### Bioinformatics assay

The PXO99A genome was downloaded from the NCBI database (GenBank accession number NC_010717.2). The sequences of interest, PXO_02032 and PXO_02034, were analyzed using HMMER (https://www.ebi.ac.uk/Tools/hmmer/) and BlastP (https://blast.ncbi.nlm.nih.gov) with the nonredundant protein sequences database to study their structures and search for homologs.

The top 24 representative hit sequences were downloaded from the NCBI database. Clustal X and MEGA7 (57) software were employed for sequence alignment and phylogenetic analysis to study the evolutionary relationships among these sequences. For statistical analysis, the GraphPad Prism software version 9.5.1 was used. The model figure depicting the results was generated using Biorender (https://biorender.com).

### Protein purification

The gene of interest was cloned into a pET vector and subsequently transformed into *E. coli* BL21(DE3) cells. The cells were grown in LB medium containing appropriate antibiotics until reaching an OD_600_ of 0.6. At this point, the inducer IPTG was added, and the cells were incubated at 20°C for 18 hours. After induction, the cells were collected by centrifugation and resuspended in lysis buffer (50 mM NaH_2_PO_4_, pH 8.0, 300 mM NaCl, and 10 mM imidazole). The cells were then subjected to sonication, and the sonicated lysates were centrifuged at 15,000 × *g* for 30 minutes at 4°C. The resulting supernatants were incubated with Ni-NTA resin (Smart Lifesciences) for protein purification. The proteins of interest were eluted using elution buffer (50 mM NaH_2_PO_4_, pH 8.0, 300 mM NaCl, and variable concentrations of imidazole) and subsequently analyzed by SDS-PAGE and Western blotting to confirm their expression and purification.

### Plant transformation

The *tleB* gene fragment was amplified using PXO99A genomic DNA as a template and cloned into the binary destination vector pl34-eGFP using Gibson recombinase to obtain the recombinant plasmid pl34-*tleB*-eGFP. This plasmid was transformed into *Agrobacterium tumefaciens* strain GV3101. The transformed *Agrobacterium* was then used to introduce the *tleB*-eGFP construct into *A. thaliana* Col-0 plants via floral dipping. Positive transgenic plants were screened on hygromycin resistance plates.

### Crystallization, data collection, and structure determination

The protein was crystallized using the vapor diffusion method at a protein concentration of approximately 10 mg/mL. Crystals were obtained after approximately 3 days from the following crystallization conditions: 0.1 M bis-tris, pH 6.8, 23% PEG3350 at 20°C. The crystals were flash-frozen in liquid nitrogen after soaking in cryoprotectant (20% glycerol added to crystallization buffer). X-ray diffraction data were collected at beamline BL18U1 of the National Center for Protein Sciences and processed using XDS. The data were merged and normalized using Aimless within the CCP4 program suite (58). The crystal structure was determined by molecular replacement using the Phaser program embedded in the PHENIX suite (59), and the truncated structure of Tle1 (PDB ID:4O5P) was used as the search model. Iterative refinement and model building were performed by PHENIX.refine (59) and Coot (60) to obtain a complete structure. Statistical data summarizing data collection and refinement are provided in Table S1.

### Enzymatic assay

For the hydrolysis of lecithin, the substrate was initially dissolved in ethanol at a concentration of 200 mM, followed by a 20-fold dilution in PBS containing 1% Triton X-100. Each reaction mixture consisted of 100 µL of this diluted solution and 10 µg of TleB. Following incubation at 30°C for 2 hours, the resulting products were subjected to UPLC-QTOFMS for detection and analysis. Similarly, for the hydrolysis of phosphatidylserine, the substrate was dissolved in 1× PBS supplemented with 0.5% methanol to achieve a final concentration of 1 mg/mL. Each reaction was set up with 100 µg of TleB and 400 µg of phosphatidylserine. After incubation at 28°C for 3 hours, the resulting products were subjected to UPLC-QTOFMS for detection and analysis.

### Infection assay

To infect *A. thaliana* Col-0 inflorescences, a 200 µL PCR tube containing 50 µL of bacterial solution was inverted on a stick. The bacterial solution, with an optical density (OD_600_) of 0.8, was prepared by resuspending bacteria in a 10 mM MgCl_2_ solution containing 0.1% Triton X-100 (Sigma T9284). Before the infection, blooming flowers were removed. The top of the *A. thaliana* Col-0 inflorescence was then immersed in the bacterial solution for 3 hours. After incubation, the PCR tube was removed, and the plants were cultured under normal greenhouse conditions (20°C–22°C, 16 hours of light, and 8 hours of darkness). Subsequently, the total number of seeds in six to eight mature siliques was counted after 7–10 days of culture for further analysis.

### Observing bacterial ovule colonization

*A. thaliana* inflorescences were treated with bacteria for 3 hours and dissected after 24 hours of light culture. The pistils were immersed in 4% glutaraldehyde, subjected to a 10-minute vacuum treatment, and washed five times with PBS buffer for 5 minutes each. Subsequently, the samples were dehydrated using an ethanol gradient (45%, 55%, 70%, 85%, 95%, 100%, and 100%). The dehydrated samples were then subjected to carbon dioxide critical point drying, followed by gold spraying, and finally observed using a scanning electron microscope.

### Observing fluorescent reporter plants labeled by pDR5::eGFP using confocal microscopy

The PXO99A wild-type and Δ*tleA-tleB* strains (lacking effector and immunity proteins) were separately used to infect inflorescences of fluorescent reporter plants expressing *pDR5::Egfp* (61). The MgCl_2_ solution (10 mM) was used as a control. After 24 hours of infection, *Arabidopsis* pistils were fixed in 4% paraformaldehyde, vacuumed for 1 hour, washed twice with PBS, and then immersed in ClearSee solution (containing 10 g xylitol, 15 g sodium deoxycholate, and 25 g urea, in ddH_2_O to a final volume of 100 mL) to achieve transparency for over 7 days. The fluorescence of DR5 in the ovules was observed using a laser confocal microscope (Nikon Ni-E A1 HD25). Images were captured and saved. Subsequently, ImageJ was used to calculate the fluorescence grayscale and compare the distribution of DR5 in the inflorescences under different treatment conditions.

### Fat-Western blotting

Lipid-protein binding was examined using fat-Western blotting. Nitrocellulose membranes containing different lipids (PIP Strips membrane; P23751 purchased from Thermo Fisher) were used. The nitrocellulose membrane was incubated in a 3% fatty acid-free BSA (wt/vol) TBST solution (fatty acid-free serum albumin, pH 7.0, CAS 9048-46-8, purchased from Sangon Biotech) for 1 hour at room temperature, followed by three washes with TBST for 10 minutes each.

The membrane was then incubated with TBST solution containing purified TleB protein overnight at 4°C, followed by three 10-minute washes with TBST. Subsequently, the membrane was incubated with anti-His tag antibody (Abcam) for 1 hour at room temperature, washed three times with TBST, and incubated with a mouse secondary antibody for 1 hour at room temperature. After three washes with TBST buffer for 10 minutes each, the protein signals were detected using an ECL luminescent solution and a chemiluminescence imaging system.

### Protein secretion assay

Bacteria were grown in NB liquid medium at 28°C until reaching an OD_600_ of 1 and then centrifuged at 2,500 × *g* for 8 minutes to collect the bacteria. The pellets were resuspended in fresh NB and incubated at 28°C for 1 hour. Cells were centrifuged twice at 10,000 × *g* for 4 minutes each at room temperature. The microspheres were resuspended in 2× SDS loading buffer (Epizyme Biotech) and used as whole-cell samples. Trichloroacetic acid (20% [vol/vol]) was added to the supernatant, which was then precipitated at −20°C for 30 minutes and centrifuged at 15,000 × *g* for 30 minutes at 4°C. The precipitate was washed with acetone, air-dried, and resuspended in 2× SDS loading buffer. Whole-cell and secretion samples were boiled at 98°C for 10 minutes and subjected to SDS-PAGE and Western blot analysis.

### Phospholipid extraction and mass spectrometry analyses

Inflorescences from 30-day-old bacterially treated *A. thaliana* plants were collected and immediately immersed in 3 mL of 75°C preheated isopropyl alcohol containing 0.01% butylated hydroxytoluene (BHT; Sigma B1378) for 15 minutes. The isopropyl alcohol with BHT was prepared in 50 mL glass tubes with Teflon-lined screw caps. Subsequently, 1.5 mL of chloroform and 0.6 mL of Watson’s drinking water were added to the tubes, and the mixture was incubated on a circular shaker at room temperature for 1 hour. The lipid extract was transferred to a fresh glass tube with a Teflon-lined screw cap. An additional 4 mL of chloroform/methanol (2:1, containing 0.01% BHT) was added, and the mixture was shaken for 30 minutes. This extraction step was repeated for all samples until they turned white, which typically required the same number of extractions per sample. Next, 1 mL of 1 M KCl was added to the combined extract, mixed thoroughly, and centrifuged at 4,500 × *g* for 3 minutes at 4°C. The lower phase was collected using a 5 mL pipette. This was followed by the addition of 2 mL water, mixing, and a second centrifugation at 4,500 × *g* for 3 minutes at 4°C. The lower phase was again collected with a 5 mL pipette. The solvent was evaporated under nitrogen, and the samples were stored at −80°C. The extracted leaves were dried in a 105°C oven and weighed to determine the dry weight.

The sample was dried under nitrogen and resuspended in 800 µL of chloroform using 8 mg of inflorescence dry weight extract for detection. Each sample was then supplemented with 30 µL of an internal reference standard mixture (3 µL of 17:0-20:4-PC, 3 µL of 17:0-20:4-PE, 3 µL of 21:0-22:6-PG, 3 µL of 17:0-20:4-PI, and 18 µL of 17:0-14:1-PS [purchased from Avanti Polar Lipids]) and brought up to a total volume of 500 µL with chloroform. The samples were analyzed by mass spectrometry using a Shimadzu CBM-20A Lite HPLC system coupled with a Triple Quad 5500 QTRAP liquid chromatography-tandem mass spectrometer.

### Fluorescence microscopy analysis of TliA and TliB subcellular localization in *E. coli*

Overnight cultures grown in LB medium with appropriate antibiotics were diluted 1:100 in fresh LB medium with antibiotics and grown to an OD_600_ of 0.6. Expression was induced with 0.1% arabinose for 1 hour. Cells were resuspended in 0.5× PBS to an OD_600_ of 10 and spotted onto 1% agarose–0.5× PBS pads for imaging (Nikon Ti-E A1R HD25+SIM S).

### Confocal microscopy analysis of TleB-eGFP subcellular localization in *Arabidopsis* seedling roots

*A. thaliana* seeds were sown on 1/2 MS medium and vernalized at 4°C for 2 days before being transferred to a plant growth chamber at 22°C for 6 days. Uniformly grown seedlings were selected, immersed in 4 µM FM4-64 dye for 3 minutes, washed to remove excess dye, and examined by confocal microscopy.

## Data Availability

All data are available in the main text or the supplemental material.

## SUPPLEMENTAL MATERIAL

Supplemental materials mbio.01546-25-s0001.pdf Fig. S1 to S3; Tables S1 to S3.

Data S1 mbio.01546-25-s0002.pdf TleB homologs sequence for Fig. S1.

Data S2 mbio.01546-25-s0003.pdf TleB homologs sequence for Fig. 2K.

ASM does not own the copyrights to Supplemental Material that may be linked to, or accessed through, an article. The authors have granted ASM a non-exclusive, world-wide license to publish the Supplemental Material files. Please contact the corresponding author directly for reuse.
