# Supplementary material for: A phospholipase effector of the type VI secretion system modulates plant reproduction
Source: mBio. 2025 Aug 5;16(9):e01546-25. doi: 10.1128/mbio.01546-25 (PMC12421835; doi:10.1128/mbio.01546-25)
Supplement: Supplemental materials — Fig. S1 to S3; Tables S1 to S3. [file mbio.01546-25-s0001.pdf]

Supplementary Materials for

A phospholipase effector of the type VI secretion system modulates plant reproduction

Zhi-Min Tan[1,2#], Jing-Ting Yang[1#], Qing-Jie Xiao[3], Jing-Tong Su[2], Zeng-Hang Wang[1,2], Yu-Tong Jiang[1,4], Jin-Sheng Liu[1], Tong-Tong Pei[2], Xiaoye Liang[2], Ying An[2], Hong-Wei Xue[4,5], Wen-Ming Qin[3], Wen-Hui Lin[1*] and Tao Dong[2*]

*Correspondence: dongt@sustech.edu.cn and whlin@sjtu.edu.cn

The PDF file includes:

Materials
Figs. S1 to S3
Tables. S1 to S3

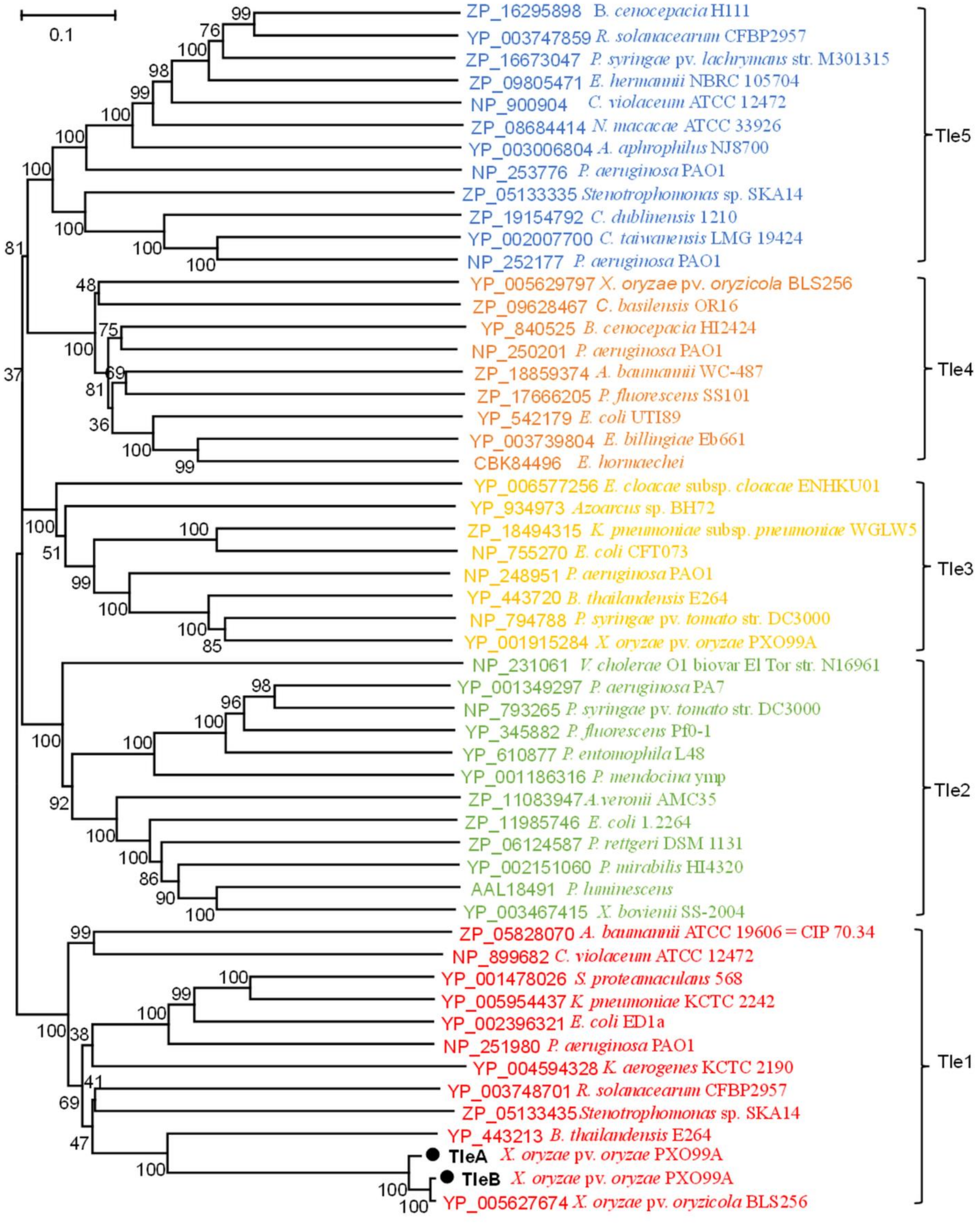

Figure S1. Phylogenetic analysis of TleA and TleB with representative members of the Tle1-5 families. MEGA7.0 was employed to build Neighbor-Joining phylogeny with bootstrap 1000 times. Protein sequences are provided in Supplementary Data 1

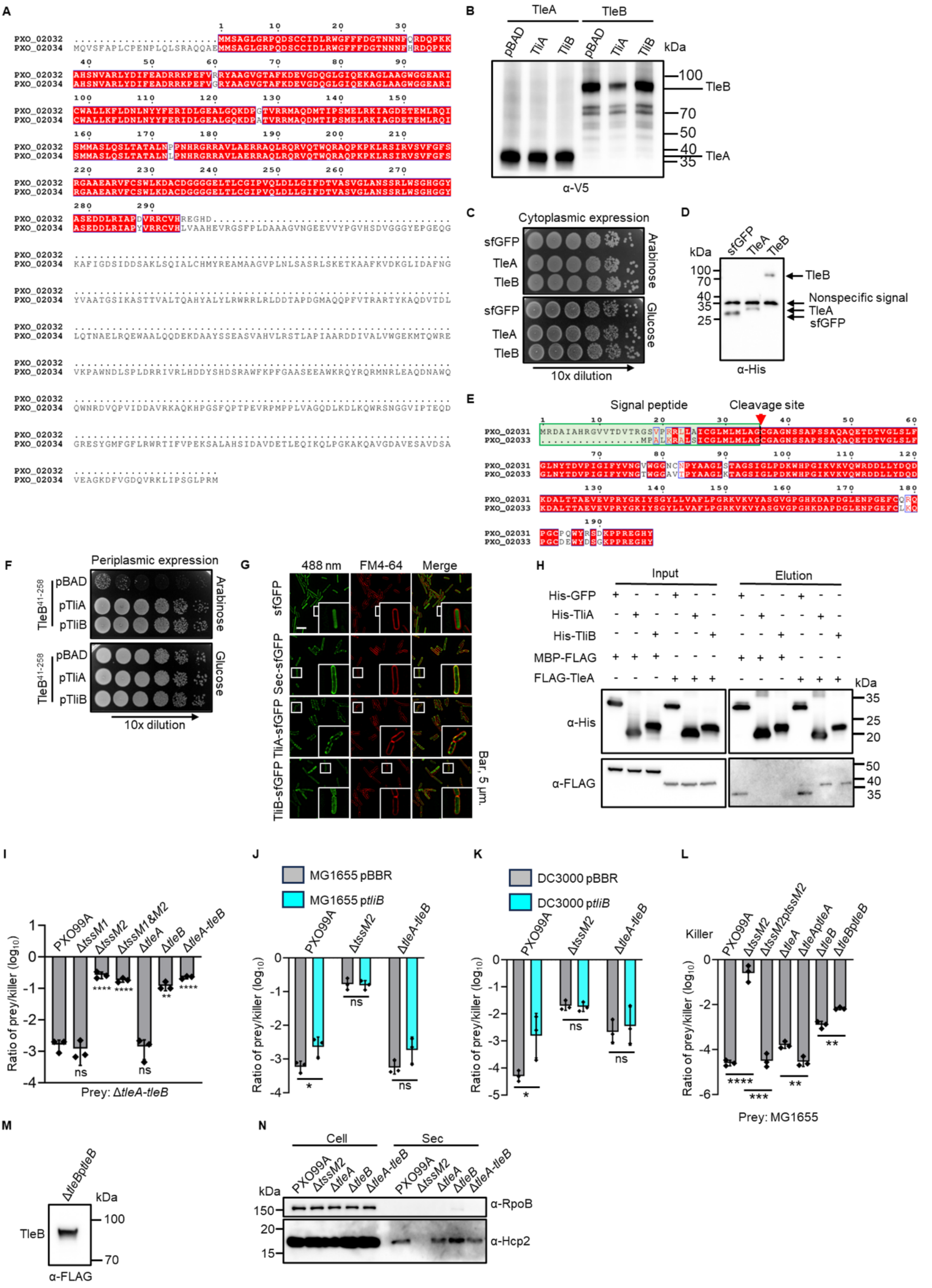

Figure S2. Characterization of PXO99A effector proteins. (A) Sequence alignment of the PXO_02032 (TleA) and PXO_02034 (TleB) proteins. TleA and TleB share 98% identity and 39% coverage. (**B**) Western blotting analysis of TleA and TleB expression shown in Fig. 1B. (**C**) Toxicity

of expressing TleA and TleB in *E. coli* cytoplasm. TleA and TleB were expressed on pBAD vectors. The expression of sfGFP, TleA, and TleB was detected by Western blotting analysis, and the result is shown in (**D**). (**E**) Sequence alignment of the PXO_02031 (TliA) and PXO_02033 (TliB) proteins. Conserved amino acids are highlighted in the red box. For (**A**) and (**E**) alignment views were generated using ESPript (https://espript.ibcp.fr/ESPript/ESPript/). Conserved amino acids are highlighted in red, and predicted signal peptides are enclosed in the green box. (**F**) Toxicity of expressing the N-terminal DUF2235 domain of *tleB* with an empty vector (pBAD) or a vector carrying the immunity gene *tliA* or *tliB* together as indicated in *E. coli*. All genes were cloned on pBAD vectors. Glucose and arabinose were used for repression and induction, respectively. (**G**) *E. coli* cells expressing TliA-sfGFP or TliB-sfGFP from the pBAD vector were cultured under repression (0.2% glucose) or induction (0.2% arabinose) conditions. Cells were imaged using structured illumination microscopy (SIM). (**H**) Interaction of TleA with TliA or TliB. Pull-down analysis was performed using His-GFP (control), His-TliA or TliB, MBP-FLAG (control), and FLAG-TleA. (**I**) Competition assay of PXO99A wild type, T6SS-1-null mutant (Δ*tssM1*), T6SS-2-null mutant (Δ*tssM2*), T6SS-1&2-null mutant (Δ*tssM1&M2*), or effector-immunity deletion mutants (Δ*tleA*, Δ*tleB*, and Δ*tleA-tleB*) against prey effector-immunity deletion mutants (Δ*tleA-tleB*). (**J**) Competition assay of PXO99A wild type, T6SS-2-null mutant (Δ*tssM2*), or effector-immunity deletion mutants (Δ*tleA-tleB*) against prey MG1655 with or without immunity protein TliB. (**K**) Competition assay of PXO99A wild type, T6SS-2-null mutant (Δ*tssM2*), or effector-immunity deletion mutants (Δ*tleA-tleB*) against prey *P. syringae* pv. *tomato* DC3000 with or without immunity protein TliB. (**L**) The PXO99A wild-type (WT), a T6SS-2-null mutant (Δ*tssM2*), and Δ*tssM2* complemented with the structural gene *tssM2*, effector-immunity deletion mutants Δ*tleA* and Δ*tleB*, as well as their respective complemented strains expressing *tleA* or *tleB* against prey MG1655, and prey survival was quantified to evaluate T6SS-2-dependent killing. (**M**) Protein expression was assessed in the effector-immunity deletion mutant Δ*tleB* complemented with *tleB*. Gene was expressed from plasmid with C-terminal FLAG tag. Whole-cell lysates were separated by SDS-PAGE and analyzed by immunoblotting using anti-FLAG antibodies to detect TleB. (**N**) Secretion assay of Hcp2 in the wild type (PXO99A), Δ*tssM2*, Δ*tleA*, Δ*tleB*, and Δ*tleA-tleB* mutants. RpoB serves as a control for cytosolic expression and cell lysis. Error bars indicate the mean ± standard deviation of three biological replicates and statistical significance was calculated using a two-tailed Student's *t*-test. $*p < 0.05$, $**p < 0.01$, $***p < 0.001$, $****p < 0.0001$

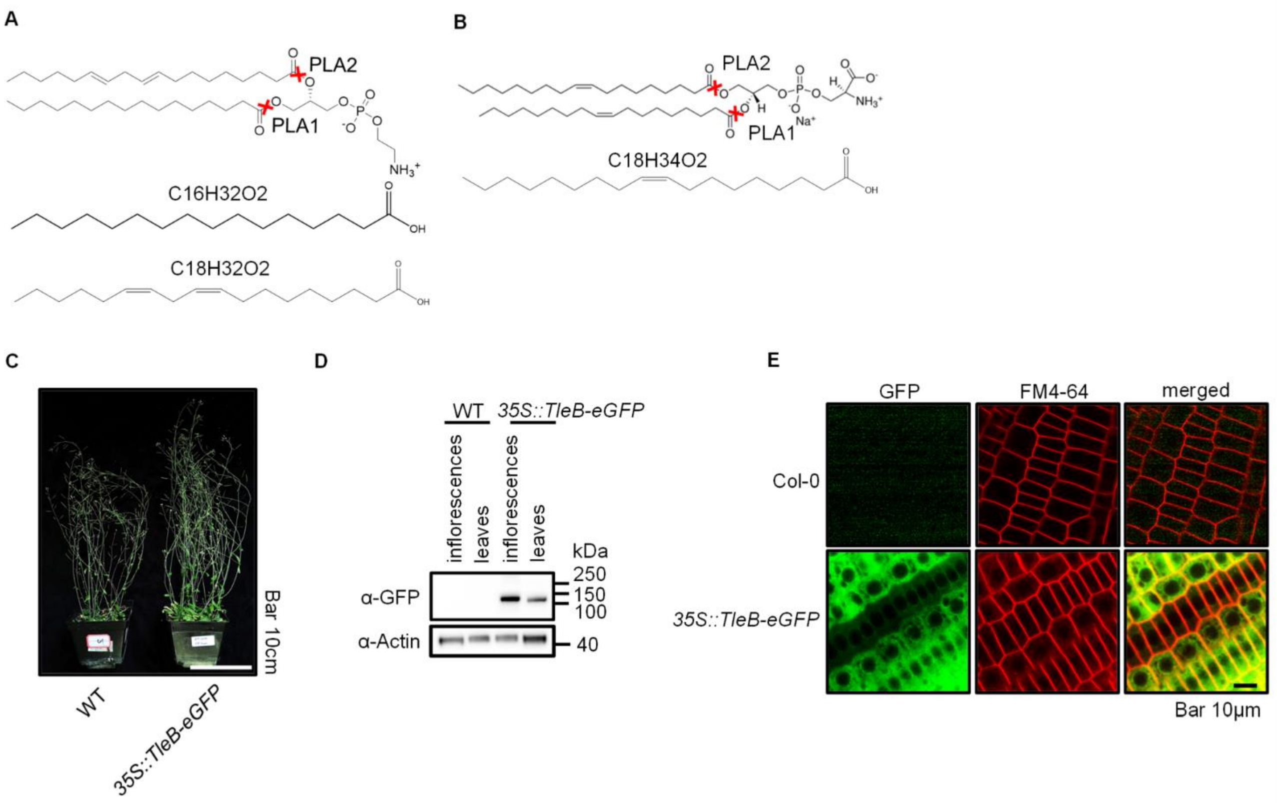

Figure S3. The *in vitro* and *in vivo* activities of TleB. (**A**) Characterization of the TleB cleavage sites on phosphatidylcholine. (**B**) Characterization of the TleB cleavage sites on phosphatidylserine. For (A) and (B), the substrates were co-incubated with TleB proteins, and the digested products were analyzed by mass spectrometry. (**C**) The appearance of transgenic *A. thaliana* plants expressing TleB. After 39 days of greenhouse culture, the plants were photographed to compare the effect of TleB expression on *A. thaliana*. (**D**) Western blotting analysis of TleB-eGFP in transgenic plants. Inflorescences and leaves from 4-week-old *A. thaliana* Col-0 and transgenic plants expressed TleB-eGFP were collected for crude total protein extraction, followed by Western blotting analysis. (**E**) Fluorescence microscopy analysis of TleB-eGFP subcellular localization in *Arabidopsis* seedling roots.

Data	TleB
Integration Package	XDS
Beamlines	BL18U1
Space Group	P121
Unit Cell (Å)	a=66.6, b=70.4, c=78.8
Unit Cell (°)	α=90, β=99.3, γ=90
Wavelength (Å)	0.9793
Resolution (Å)	46.65-1.9
Rmerge (%)	17.8 (160)
CC1/2	99.6 (45.1)
I/sigma	10.2 (1.3)
Completeness (%)	99.9(100)
Number of measured reflections	379327
Number of unique reflections	56744
Redundancy	6.7 (6.8)
R_{work} / R_{free} (%)	17.26/19.63
No. atoms	
Protein	5375
Waters	494
Average B factor ($Å^2$)	29.1
R.m.s. deviations	
Bond lengths (Å)	0.01
Bond angles (°)	1.02
Ramachandran plot statistics (%)	
Most favorable	97.50
allowed	2.5
Disallowed	0

Table S1. Data collection and structure statistics

Table S2. Plasmids and strains

Plasmid	Description	Reference
pBAD24kan-Sec signal-*tleA*-3V5	Arabinose induces expression of TleA with a C-terminal 3V5 tag	This study
pBAD24kan-Sec signal-*tleB*-3V5	Arabinose induces expression of TleB with a C-terminal 3V5 tag	This study
pBAD18cm	Arabinose induces vector, chloramphenicol resistance	Lab stock
pBAD24cm-*tliA*	Arabinose induces expression of *tliA*	This study
pBAD24cm-*tliB*	Arabinose induces expression of *tliB*	This study
pK18mobSacB	Suicidal conjugation vector for all chromosomal allelic changes	He Yawen Lab
pK18mobSacB-*tssB1*	Suicidal conjugation vector to construct in-frame deletion mutant of *tssB1* (PXO_00266)	This study
pK18mobSacB-*tssB2*	Suicidal conjugation vector to construct in-frame deletion mutant of *tssB2* (PXO_02045)	This study
pk18mobSacB-*tssM2*	Suicidal conjugation vector to construct in-frame deletion mutant of *tssM2* (PXO_04696)	This study
pK18mobSacB-*tleA*	Suicidal conjugation vector to construct in-frame deletion mutant of *tliA* (PXO_02031), *tleA* (PXO_02032)	This study
pK18mobSacB-*tleB*	Suicidal conjugation vector to construct in-frame deletion mutant of *tliB* (PXO_02033), *tleB* (PXO_02034)	This study
pK18mobSacB-*tleA&B*	Suicidal conjugation vector to construct in-frame deletion mutant of *tliA* (PXO_02031), *tleA* (PXO_02032), *tliB* (PXO_02033), *tleB* (PXO_02034)	This study
pBBR1MCS-2	A broad-host-range cloning vector, kanamycin resistance	He Yawen Lab
pBBR1MCS-5	A broad-host-range cloning vector, gentamycin resistance	He Yawen Lab
pBBR1MCS5-*tliA*	TliA (PXO_02031) stable expression plasmid	This study
pBBR1MCS5-*tliB*	TliB (PXO_02033) stable expression plasmid	This study
pET28a-His-*tliA*	IPTG inducible expression of TliA with an N-terminal His tag	This study
pET28a-His-*tliB*	IPTG inducible expression of TliB with an N-terminal His tag	This study
pET28a-His-*GFP*	IPTG inducible expression of GFP	Lab stock
pBAD24kan-MBP-FLAG	Arabinose induces expression of MBP	Lab stock

pBBR1MCSARAC-FLAG-*tleA*	Arabinose induces expression of TleA with an N-terminal FLAG tag	This study
pBBR1MCSARAC-FLAG-*tleB*	Arabinose induces expression of TleB with an N-terminal FLAG tag	This study
pET28a-9His-*tleB*	IPTG inducible expression of TleB with a N-terminal 9His tag	This study
pET28a-His-*tleB*^{S240A}	IPTG inducible expression of TleB^{S240A} with a N-terminal His tag	This study
pET28a-His-*tleB*^{H347A}	IPTG inducible expression of TleB^{H347A} with a N-terminal His tag	This study
pK18mobSacB-*tleB*^{H347A}	Suicidal conjugation vector to construct chromosomal point mutation of *tleB*^{H347A} (PXO_02034)	This study
pK18mobSacB-*hrcU*	Suicidal conjugation vector to construct in-frame deletion mutant of *hrcU* (PXO_03402)	This study
pl34-*tleB*-eGFP	TleB (PXO_02034) expression plasmid in a plant	This study
pBBR1MCSArac-*tleB*-His	Arabinose induces expression of TleB with a C-terminal His tag	This study
pBBR1MCSArac-*tleA*-His	Arabinose induces expression of TleA with a C-terminal His tag	This study
pBBR1MCSArac-sfGFP-His	Arabinose induces expression of GFP with a C-terminal His tag	This study
pBAD24Kan-Sec_*tleB*$^{(41-258)}$-3V5	Arabinose induces expression of TleB$^{(41-258)}$ with a C-terminal 3V5 tag	This study
pBAD24cm-*tliA*-sfGFP-His	Arabinose induces expression of TliA with a C-terminal His tag	This study
pBAD24cm-*tliB*-sfGFP-His	Arabinose induces expression of TliB with a C-terminal His tag	This study
pBAD24cm-sfGFP-His	Arabinose induces expression of sfGFP with a C-terminal His tag	Lab stock
pBAD24kan-Sec-sfGFP-His	Arabinose induces expression of sfGFP with a N-Sec signal peptide and C-terminal His tag	Lab stock

**Strain**	**Genotype**	**Description**	**Reference**
Xanthomonas oryzae pv. *oryzae* PXO99A	Parental	Parental strain	This study
	Δ*tssB1*	T6SS-1 null, in-frame deletion of *tssB1*	This study
	Δ*tssB2*	T6SS-2 null, in-frame deletion of *tssB2*	This study
	Δ*tssB1-tssB2*	T6SS-1&T6SS-2 null, in-frame deletion of *tssB1* and *tssB2*	This study
	Δ*tleA*	in-frame deletion of *tliA* and *tleA*	This study
	Δ*tleB*	in-frame deletion of *tliB* and *tleB*	This study
	Δ*tleA-tleB*	in-frame deletion of *tliA*, *tleA*, *tliB* and *tleB*	This study
	$tleB^{H347A}$	chromosomal point mutation of $tleB^{H347A}$ (PXO_02034)	This study
	Δ*hrcU*	in-frame deletion of *hrcU* (PXO_03402)	This study
	Δ*tssM2*	in-frame deletion of *tssM2* (PXO_04696)	This study
	Δ*tssM1*	T6SS-1 null, in-frame deletion of *tssM1*	This study
	Δ*tssM1-tssM2*	T6SS-1 and T6SS-2 null, in-frame deletion of *tssM1* and *tssM2*	This study
	Δ*hrcU-tssB1-tssB2*	T3SS, T6SS-1, and T6SS-2 null, in-frame deletion of *hrcU*, *tssB1*, and *tssB2*	This study
	Δ*tssM2-tleA-tleB*	T6SS-2 null, in-frame deletion of *tssM2* (PXO_04696), in-frame deletion of *tliA*, *tleA*, *tliB* and *tleB*	This study
E. coli T-Fast		The strain used for cloning and gene expression	TIANGEN
E. coli MG1655		The strain used for a competition assay	Lab stock
E. coli WM6026		Strain used for conjugation	Lab stock
E. coli BL21 DE3		The strain used for protein expression	Lab stock
Agrobacterium tumefaciens GV3101		The strain used for transforming plants	WEIDI
P. syringae pv. tomato DC3000		The strain used for a competition assay	Lab stock

Table S3. Primers

Primers	Sequences (5’-3’)	Descriptions
pK18mobSacB-R-Hifi1	CCGAGCTCGAATTCGTAATC	Reverse primer to amplify vector pK18mobsacB for constructing deleting plasmid
pK18mobSacB-F-Hifi1	TCGTTTTACAACGTCGTGACT	Forward primer to amplify vector pK18mobsacB for constructing deleting plasmid
tssB1-KO5	AGCCTCGCAGCTCTGCAACT	Forward primer for knockout validation
tssB1-KO1	GATTACGAATTCGAGCTCGG GCACTGCACGTGGTGTACCA	Forward primer to amplify upstream of *tssB1* for constructing deleting plasmid
tssB1-KO2	CTTCGACTTGGGAGCCTCGACGTCGTACTCGATCTG	Reverse primer to amplify upstream of *tssB1* for constructing deleting plasmid
tssB1-KO3	GAGTACGACGTCGAGGCTCCCAAGTCGAAGGATGC	Forward primer to amplify downstream of *tssB1* for constructing deleting plasmid
tssB1-KO4	AGTCACGACGTTGTAAAACGA CCTCGGTCGGGCACTTCAT	Reverse primer to amplify downstream of *tssB1* for constructing deleting plasmid
tssB1-KO6	CGATGTACCAGCGGCATCAG	Reverse primer for knockout validation
hrcU-KO1	GATTACGAATTCGAGCTCGG CAACTTGCGCTGATCGTTG	Forward primer to amplify upstream of *hrcU* for constructing deleting plasmid
hrcU-KO2	GGAAGAAAAAGCCCTGCCATGCTAGGAG	Reverse primer to amplify upstream of *hrcU* for constructing deleting plasmid
hrcU-KO3	ATGGCAGGGCTTTTTCTTCCGACATTGCCTTAT	Forward primer to amplify downstream of *hrcU* for constructing deleting plasmid
hrcU-KO4	AGTCACGACGTTGTAAAACGAAGCTCGTGCATCAA CTTGATCT	Reverse primer to amplify downstream of *hrcU* for constructing deleting plasmid
hrcU-KO5	GCGAAGTCAGGAGCGGTTTT	Forward primer for knockout validation
hrcU-KO6	CGTCTGCACGGCGTTCTT	Reverse primer for knockout validation
tssB2-ko5	AGCGGATAACAATTTCACACAGGA	Forward primer for knockout validation

tssB2-ko1	CGGTACCCGGGGATCCTCTAGAAGGTCGCTGAGTTCCTTCTC	Forward primer to amplify upstream of *tssB2* for constructing deleting plasmid
tssB2-ko2	ATGGCTAAGAAGGAAGAAAAGGAGAAGTAAGCCATGG	Reverse primer to amplify upstream of *tssB2* for constructing deleting plasmid
tssB2-ko3	TTACTTCTCCTTTTCTTCCTTCTTAGCCATTGGTG	Forward primer to amplify downstream of *tssB2* for constructing deleting plasmid
tssB2-ko4	TGCCTGCAGGTCGACTCTAGAGGCGGCTACAATAAGAACCA	Reverse primer to amplify downstream of *tssB2* for constructing deleting plasmid
tssB2-ko6	CGCCAGGGTTTTCCCAGTCACGAC	Reverse primer for knockout validation
tssM2-KO5	gcaggtgccggaactggat	Forward primer for knockout validation
tssM2-KO1	GATTACGAATTCGAGCTCGG aaggcccgttggagcatcac	Forward primer to amplify upstream of *tssM2* for constructing deleting plasmid
tssM2-KO2	ttggccaccttcttggagattgctgaacatcgggtgct	Reverse primer to amplify upstream of *tssM2* for constructing deleting plasmid
tssM2-KO3	atgttcagcaatctccaagaaggtggccaatgagtc	Forward primer to amplify downstream of *tssM2* for constructing deleting plasmid
tssM2-KO4	AGTCACGACGTTGTAAAACGAcatattcatgtccccaggaaagt	Reverse primer to amplify downstream of *tssM2* for constructing deleting plasmid
tssM2-KO6	gaacgcggttatcaggccact	Reverse primer for knockout validation
PXO- PXO-*tleA*-sec-F	TAGCGTTTAGCGCATCGGCGATGATGAGCGCGGGGCTG	Forward primer to amplify *tleA*
PXO-*tleA*-3v5-r	GGATTAGGAATAGGTTTACCGCATGCGTCATGTCCCTCCCTATGCACGC	Reverse primer to amplify *tleA*
pBAD24-sec-hifi-r	CGCCGATGCGCTAAACGC	Forward primer to amplify pBAD24-sec
pBAD24-V5-hifi-f	GGTAAACCTATTCCTAATCCTCTCCTT	Reverse primer to amplify pBAD24-sec
PXO-*tleB*-sec-F	TAGCGTTTAGCGCATCGGCGATGCAAGTTTCCTTCGCTCCA	Forward primer to amplify *tleB*

PXO-*tleB*-3v5-r	GGATTAGGAATAGGTTTACCGCATGCCATCCTCGG CAAGCCACTT	Reverse primer to amplify *tleB*
pBAD24-hifi-R3	cgtttcctcctgctagcccaaaaaaac	Reverse primer to amplify pBAD24cm
pBAD24cm-*tliA*-F	ttgggctagcaggaggaaacg ATGCGTGACGCGATCGCG	Forward primer to amplify *tliA*
pBAD24cm-*tliA*-R	gccaaaacagccaagcttTca TCAGTAGTGACCCTCCCTGGGCG	Reverse primer to amplify *tliA*
pBAD24-hifi-F3	tgAaagcttggctgttttggcg	Forward primer to amplify pBAD24cm
pBAD24cm-*tliB*-F	ttgggctagcaggaggaaacgATGCCAGCATTGAAACGTGCG	Forward primer to amplify *tliB*
pBAD24cm-*tliB*-R	gccaaaacagccaagcttTcaTCAGTAATGACCCTCCCTGGGC G	Reverse primer to amplify *tliB*
tliA-tleA -KO5	CGTGGCTGGACGCGCTGT	Forward primer for knockout validation
tliA-tleA -KO1	GATTACGAATTCGAGCTCGG TGCAGACGCGCTTGCCGT	Forward primer to amplify upstream of PXO-02031 for constructing deleting plasmid
tliA-tleA -KO2	TATGCACGCAGATCGCGTCACGCATCCTGC	Reverse primer to amplify upstream of PXO-02031 for constructing deleting plasmid
tliA-tleA -KO3	TGACGCGATCTGCGTGCATAGGGAGGGACATG	Forward primer to amplify downstream of PXO-02032 for constructing deleting plasmid
tliA-tleA -KO4	AGTCACGACGTTGTAAAACGA TCCGACACCAGACGCATAGACCTT	Reverse primer to amplify downstream of PXO-02032 for constructing deleting plasmid
tliA-tleA -KO6	TTCGCCAGGATTCTCCAAACCAT	Reverse primer for knockout validation
tliB-tleB -KO5	CGTCGATGGAACTGCGCAAGAT	Forward primer for knockout validation
tliB-tleB -KO1	GATTACGAATTCGAGCTCGG GATGCTGCGGCAGATCAGCAT	Forward primer to amplify upstream of PXO-02033 for constructing deleting plasmid
tliB-tleB-KO2	TCCTCGGCAATTTCAATGCTGGCATCACGGTCT	Reverse primer to amplify upstream of PXO-02033 for constructing deleting plasmid

tliB-tleB-KO3	AGCATTGAAATTGCCGAGGATGTGAGGGGAAG	Forward primer to amplify downstream of PXO-02034 for constructing deleting plasmid
tliB-tleB-KO4	AGTCACGACGTTGTAAAACGA CGGTGAGCCTGCAGGCACAT	Reverse primer to amplify downstream of PXO-02034 for constructing deleting plasmid
tliB-tleB-KO6	GCCAGCACACCGCGCTGT	Reverse primer for knockout validation
tliA-tleB -KO2	TCCTCGGCAAGATCGCGTCACGCATCCTGC	Reverse primer to amplify upstream of PXO-02031 for constructing deleting plasmid
tliA-tleB -KO3	TGACGCGATCTTGCCGAGGATGTGAGGGGAAG	Forward primer to amplify downstream of PXO-02034 for constructing deleting plasmid
pBBR-*tliA*-F	tggcggccgctctagaactagTCAGTAGTGACCCTCCCTGGGC	Forward primer to amplify PXO-02031
pBBR-R-hifi	ctagttctagagcggccgcca	Reverse primer to amplify vector pBBR
pBBR-*tliA*-R	ctcgaggtcgacggtatcgataATGCGTGACGCGATCGCG	Reverse primer to amplify PXO-02031
pBBR-F-hifi	tatcgataccgtcgacctcgag	Forward primer to amplify vector pBBR
pBBR- *tliB*-F	tggcggccgctctagaactagTCAGTAATGACCCTCCCTGGGC	Forward primer to amplify PXO-02033
pBBR- *tliB* -R	ctcgaggtcgacggtatcgataATGCCAGCATTGAAACGTGCG	Reverse primer to amplify PXO-02033
pET- *tliA* -F	gtgccgcgcggcagccatatgtgcggcgctggcaattcg	Forward primer to amplify PXO-02031
pET- *tliA*-R	ctcgagtgcggccgcaagctttcagtagtgaccctccctgggcg	Reverse primer to amplify PXO-02031
pET- *tliB* -R	ctcgagtgcggccgcaagctttcagtaatgaccctccctgggc	Reverse primer to amplify PXO-02033
pBBR1-flag-Hifi-F	CTTGTCATCGTCGTCCTTGTAATCg	Forward primer to amplify vector pBBR
pBBR-flag- *tleB* -F	TACAAGGACGACGATGACAAGatgcaagtttccttcgctcca	Forward primer to amplify PXO-02034
pBBR-flag- *tleB* -R	tctgcgttctgatttaatctgtaTTAcatcctcggcaagccact	Reverse primer to amplify PXO-02034
pBBR1-flag-Hifi-R1	TAAtacagattaaatcagaacgcagaagcg	Reverse primer to amplify vector pBBR
pBBR-flag- *tleA* -F	TACAAGGACGACGATGACAAGATGATGAGCGCGG GGCTG	Forward primer to amplify PXO-02032

pBBR-flag- *tleA* -R	tctgcgttctgatttaatctgtaTTAGTCATGTCCCTCCCTATGCACG	Reverse primer to amplify PXO-02032
pET28a- *tleB* -EcoRI-Hifi-1	agcaaatgggtcgcggatccgaattcatgcaagtttccttcgctccactgt	Forward primer to amplify PXO-02034
pET28a- *tleB* -HindIII-Hifi-2	tggtgctcgagtgcggccgcaagctttcacatcctcggcaagccactt	Reverse primer to amplify PXO-02034
pBAD24-sec-hifi-r	cgccgatgcgctaaacgc	Reverse primer to amplify vector pBAD24
pBAD24- *tleB* (41-258)-F	gcgtttagcgcatcggcgcgctgggggttcttcttcg	Forward primer to amplify 2034(41-258)
pBAD24- *tleB* (41-258)-R	attaggaataggtttaccgcatgcgtcgcaggcatccttgagcc	Reverse primer to amplify 2034(41-258)
pBAD24-V5-hifi-f1	gcatgcggtaaacctattcctaatcctctcctt	Forward primer to amplify vector pBAD24
tleB -S240A5	AACCGGTGGCCGCCACAT	Forward primer for point mutant validation
tleB -S240A1	GATTACGAATTCGAGCTCGG TCGATCAGGCCCTTGTCCACC	Forward primer to amplify upstream of PXO-02034 for constructing point mutant plasmid
tleA -S217A2	GTTCGGCTTCGCGCGCGGCGCGGCCGAG	Reverse primer to amplify upstream of PXO-02034 for constructing point mutant plasmid
tleB -S240A3	CCGCGCCGCGCGCGAAGCCGAACACCGA	Forward primer to amplify downstream of PXO-02034 for constructing point mutant plasmid
tleB -S240A4	AGTCACGACGTTGTAAAACGA CGACATTTTCGAGGCCGACCG	Reverse primer to amplify downstream of PXO-02034 for constructing point mutant plasmid
tleB -S240A6	CCGAAGAAGGCGCATAGCAATGT	Reverse primer for point mutant validation
tleB -H347A5	CCACACCAGCGCGACGATGT	Forward primer for point mutant validation
tleB -H347A1	GATTACGAATTCGAGCTCGG CAGTGTGCTTCGCAACACGTGG	Forward primer to amplify upstream of PXO-02034 for constructing point mutant plasmid
tleB -H347A2	CCCCGGCGTGGCCTCGGACGTGGGCGGC	Reverse primer to amplify upstream of PXO-02034 for constructing point mutant plasmid

tleB -H347A3	CCACGTCCGAGGCCACGCCGGGTACAC	Forward primer to amplify downstream of PXO-02034 for constructing point mutant plasmid
tleB -H347A4	AGTCACGACGTTGTAAAACGA CGGCGACGAAACCGAGATGCT	Reverse primer to amplify downstream of PXO-02034 for constructing point mutant plasmid
tleB -H347A6	AGGACATGACGATTCCGTCGATGG	Reverse primer for point mutant validation
pl34- *tleB* -F	ctctctctcaagcttggatccATGCAAGTTTCCTTCGCTCCA	Forward primer to amplify PXO-02034
pl34- *tleB* -R	caccatactagttctggatccCATCCTCGGCAAGCCACTT	Reverse primer to amplify PXO-02034
tleB -F	GGCTAGCAGGAGGAATTCACC ATGCAAGTTTCCTTCGCTCC	Forward primer to amplify PXO-02034
tleB -R-His	TCAGTGGTGATGATGGTGATG CATCCTCGGCAAGCCACT	Reverse primer to amplify PXO-02034
tleA -F	GGCTAGCAGGAGGAATTCACCATGATGAGCGCGGG GCTG	Forward primer to amplify PXO-02032
tleA -R-His	TCAGTGGTGATGATGGTGATGGTCATGTCCCTCCCT ATGCA	Reverse primer to amplify PXO-02032
pBBR-sfGFP-His-F	ggaggaattcaccatggtaccGtctaaaggtgaagaactgttcacc	Forward primer to amplify sfGFP
pBBR-sfGFP-His-R	tgcctgcaggtcgactctagagtggtgatgatggtgatgtttgtagagctcatccatgc	Reverse primer to amplify sfGFP
TliB-sfGFP-1	TGATGAAAGCTTGGCTGTTTTG	primer to amplify element 1 for plasmid pBAD24cm-*tliB*-sfGFP-His
TliB-sfGFP-2	GATAAGCTGTCAAACATGAGCAGATC	primer to amplify element 1 for plasmid pBAD24cm-*tliB*-sfGFP-His
TliB-sfGFP-3	ctcatgtttgacagcttatcATCGATGCATAATGTGCCTGTC	primer to amplify element 2 for plasmid pBAD24cm-*tliB*-sfGFP-His
TliB-sfGFP-4	tgcggccgcGTAATGACCCTCCCTGGGCG	primer to amplify element 2 for plasmid pBAD24cm-*tliB*-sfGFP-His
TliB-sfGFP-5	agggtcattacGCGGCCGCAGGAGGAGGA	primer to amplify element 3 for plasmid pBAD24cm-*tliB*-sfGFP-His

TliB-sfGFP-6	aaacagccaagctttcatcaGTGGTGATGATGGTGATGTTTGTAG	primer to amplify element 3 for plasmid pBAD24cm-*tliB*-sfGFP-His
TliA-sfGFP-4	tgcggccgcGTAGTGACCCTCCCTGGGCG	Forward primer to amplify TliA
TliA-sfGFP-5	agggtcactacGCGGCCGCAGGAGGAGGA	Reverse primer to amplify TliA
