# Supplementary material for: A phospholipase effector of the type VI secretion system modulates plant reproduction
Source: mBio. 2025 Aug 5;16(9):e01546-25. doi: 10.1128/mbio.01546-25 (PMC12421835; doi:10.1128/mbio.01546-25)
Supplement: Data S1 — TleB homologs sequence for Fig. S1. [file mbio.01546-25-s0002.pdf]

Supplementary Data 1. TleB homologs sequence for Figure S1.

>PXO_02032
MMSAGLGRPQDSCCIDLRWGFFFDGTNNNFQRDQPKKAHSNVARLYDIFEADRRKPEFVRRY
AAGVGTAFKDEVGDQGLGIQEKAGLAAGWGGEARICWALLKFLDNLNYYFERIDLGEALGQ
KDPGTVRRMAQDMTIPSMELRKIAGDETEMLRQISMMASLQSLTATALNPPNHRGRRAVLAER
RAQLRQRVQTWQRAQPKPKLRSIRVSVFGFSRGAAEARVFCSWLKDACDGGGGELTLCGIPV
QLDLLGIFDTVASVGLANSSRLWSGHGGYASEDDLRIAPDVRRCVHREGHD
>PXO_02034
MQVSFAPLCPENPLQLSRAQQAEMMSAGLGRPQDSCCIDLRWGFFFDGTNNNFHRDQPKKAH
SNVARLYDIFEADRRKPEFVGRYAAGVGTAFKDEVGDQGLGIQEKAGLAAGWGGEARICWAL
LKFLDNLNYYFERIDLGEALGQKDPATVRRMAQDMTIPSMELRKIAGDETEMLRQISMMASLQ
SLTATALNLPNHRGRRAVLAERRAQLRQRVQTWQRAQPKPKLRSIRVSVFGFSRGAAEARVFC
SWLKDACDGGGGELTLCGIPVQLDLLGIFDTVASVGLANSSRLWSGHGGYASEDDLRIAPYVR
RCVHLVAAHEVRGSFPLDAAAGVNGEEVVYPGVHSDVGGGYEPGEQGKAFIGDSIDDSAKLS
QIALCHMYREAMAAGVPLNLSASRLSKETKAAFKVDKGLIDAFNGYVAATGSIKASTTVALTQ
AHYALYLRWRRLRLDDTAPDGMAQQPFVTRARTYKAQDVTDLLQTNAELRQEWAALQQDEK
DAAYSSEASVAHVLRSTLAPIAARDDIVALVWGEKMTQWREVKPAWNDLSPLDRRIVRLHDD
YSHDSRAWFKPFGAASEEAWKRQYRQRMNRLEAQDNAWQQWNRDVQPVIDDAVRKAQKHP
GSFQPTPEVRPMPPLVAGQDLKDLKQWRSNGGVIPTEQDGRESYGMFGFLRWRTIFVPEKSAL
AHSIDAVDETLEQIKQLPGKAKQAVGDAVESAVDSAVEAGKDFVGDQVRKLIPSGLPRM
>YP_003739804
MAEEKTAGAGDGQPSTCILPEWEHNGLVKWKGIQNQKKSLDAQVVLLQPPIRVIPVIFLPGVM
GTNLMSDGSSKREQPIWRGDSEVGVYFKWAGQDGNRRRKLLSPDTTKVDNRGDINQNIYSPF
SDDGNLFPTRRERGWGGVLSFSYGKFLSVFQGALLDDWQRDMVNYAARVGGKGGILSQLVG
ARLSEDKATKIADESVMTQQELDHFRNFLFPLHVYGYNWLQDNKTSAEGLVDYIEEVIDLYTH
QHCHGMAFPAGQEKVIIVTHSMGGLVARYASQISGAQDKILGIVHGVIPDIGSPAAYRRMKVGA
KQEGMAGAVLGNTAQELMPVLARAPAPLQLLPSAKYLGGAPWLTVEGGNEDGTDIKLPKRRD
PFSEIYLNETLWCRLYEADIIDKDAAEIKRNWNAYSDLISKAVQRFIEKLDNKYHPNTYAFYGH
KIGSDGTLSWNRITQVYLKKTGDNDLKLPNNYREVPLPPHGAQVYRLGSSNTPGDGTVPVESL
NAIRQSRSIKSVLATNVDHQGAYNVNSLNDIPNKPALKFTLRAIVKMVQEVPVP
>CBK84496
MDDNKNSPPNRSYHRPIWDDDGTFHYKVFSQPKDKNHISVCLKPPTKVIPVIFIPGVMGSNLKS
GDKKVWQFSLSSLKKWPLAGPQKRKQLLDPTTTVVDDSGEILNDGADGKKFPSRHARGWGS
AFYMNYGEALDRLQYLLSDDEILMDNYFRETQLQTARQRFIGVRIGNEPQEQVLSEEEVAHGH
KFLFPLHVFGYNWLQSNADSAALLGEYIRKVLSAYHGRLAVNKVVLITHSMGGLVARHYSEN
MGGQDSILGIVHGVMPDLGSPAAYRRMKIGERGITGMIIGESAEKLMPVLAQSPGPLQLLPGM
AYGQGWLKINSKETQLSLPVADPYEEIYLNKTAWWRLCEQDLLLDDTRVECNKYEKTISNIVK
KFIEKLNGKYHPQTWLFYGASKSNPSDGFLTWEERIPLSVKEAQRSRENAANPFELSPLRSHQL
ISSASPGDGTVPITSVCTSSSRIQGVLATDVDHEGAYAVDPVDLSRSVYSDLSDALVFTVRSVVK
IVQQVPAP
>YP_542179
MTSTMHMDRADKNKVGTVEIEDLRVIPVIFLPGVMGSNLMDKKGKSIWRYDDSMSLMGWSL
PTSGPKERKRLLHPDRVEVDNRGRIPAPPDAQEKLIQLGQQYPEDPSDKEAMDNYTQAVRDIL
DNIEPEAKLFGSRKDRGWGEVANASYGSFLDVLQTALYRDKPTKKGETLSATYQQLLDVPLGL

EYGPDSLDEEYLEVIRLYQFPVHVVGYNWLGSNMLSAIRLQEQIKKIVGGYQKRGMKCHKVIL
VTHSMGGLVARYFSECLSGNTDVYGIVHGVLPSIGAAATYTRMKRGTENPESNPEGYVISHILG
RNAAEMVAVFSQSPGPMELLPMNDYGEEWLNIVDRDGSTLTLPKNLPIKEGIAPEERTYAELYL
NRENWWKLVDENLLNPFNTSLNQKQIDTDWNIYENLITESVNPFHKQIAGKYHINTYSFYGRA
KLGDIPEAHLTQENVLWKGSLSMGKKSDISLEPKFIDGRLDLNEVGNIRTIKDEFSPEEQAWEIN
TDDGDTYVKIGQRFTLRDSCENGDGTVPLRAGQIVHKNILERLAVQVSHEAAYRNPVSQAFAL
RSIIKIAQEVKKDGKMSYSD
>ZP_18859374
MVNKSIAQSGSATSPTTNKAGTYVEVPLQDTIPIIFVPGIMGSNIFNTALNKPVWKLGNGGGMV
GTIYSQMQKSPATLQNELDPLNTRVDTSGDIKVDSRLKLTEKTLRERYWGTVHWDSYGGILTY
LQMVLNNVDLNEKPIYGGGVGGGYAVIHQMKQQEAVYEWKSLLNQGESKKWTPQEPFISINQ
QEIEHLKKFHFPVYAMGYNWLQSSENGAATVAQKLDKIKQEYGTRFHKFIIVTHSMGGLLTRR
LVQLRGGDIAGVVHGVMPAEGAAAAYRRLVAGSSEGGLVSAVAANVIGKNTEHVTAVLANAP
GGLELLPSKAYNNGRPWLFLNGSGLMNGKDLQTNVVSLPKRDPYEEIYKADGVWWEMVKEE
LVDPANMVKKSNPNKSVKNIYKLKINEVKNFHDKITNKYHPCTYVNYGHDPKHSSFGTLTWT
LDRPLRGLTAEQMKKLPRATAKEIGTYRQKIVQEQMKKIKEGKGADANNRDLALENNGIRYIS
LVSGNLGVFSISNQNAPGDGTVPYQSGCAPLKQAGVKQVFKMTGFDHQGSYNNTQVKRSVLY
SIVKIIKENNIQPKYR
>ZP_17666205
MADTSQMPRIAPLSYNARGNPVHTWTLTPSHITDPVHCILPPDGVLPVIFVPGIMGSNLKSKPE
KGKGKNKAAPVWRLDAGFMGKNMWLALNWINKKAGIRQQLLHPARVEVDNKGAVPERSVG
TVIVPPEPDRKKTILALTKRYEERGWGEVSETSYHAFLLWLEDTLNSEFMPHKWPQFDIRPEHL
HTRSVEPGPVQVTELKPEMPIAMPGLGANLAAQLPSIISDELAARGGYRMPVHACGYNWLDS
NEEAASRLAVRIDELMQQYGRNCQQVILVTHSMGGLVARRCGQLPGMADKIAGVVHGVMPAI
GAPVAYRRCKVGMRDEDPIAGAVIGPTGQEVTAVFAQAPGALQLLPTQDYAPGWLRLIDERGA
PAMPRQPVKDPYEEIYLRRDRWWGLLREEWLAPKDGDPITWETFAINIKKASSFHQRIAGSYH
PQTYVVYYGNDDKYPSFETITWEMRRGSRLNGPYASSPDAFTVSSLQMPEVRDDGRSPLYVGG
QAESVMPPRGDPNAPVKTVQTSYWELHCRMQDGAGDGTVPVSSGRAPIRQIRQGSVRQQVQ
APGFDHEASYANPLTQQFTLYSLIKIAAKAKRPLCAG
>YP_005629797
MTTNENGAAASAPTGEDEWDAPQERVATRYVDAQGHTIYAWNLTSSKLTDPVKLYMPSHRIVP
IVFVPGIMGSNLKALKDIYRSGQDGKASSRKKIATKGQRIWNVDSMTSPVRADNSISWPGQDA
AARQLMLNMDAVEVDDRGSIELRSEESNVYLPDEGRTRSAQRRREEIRQARLDDKRRRGWGT
VSWCSYGAFLNWLEEQLAGATYRNGKPSLAFLELFKHVGSSPTGAIHAPPPLTEEQIKKLVKFR
FPVHAVGYNWLKSNLDSGQYLADKIAAIRKHYTDLGMDCQKVIVITHSMGGLVARAASQACS
ADKDILAVIHGVMPTDGAGAFYKRFVGGLTGEGAGVFGSLVGKVTALVLGASGRETTPVLGFG
PGPMELAPNQLYNGGKPWLFIKDAHGKTLLSLPERGNPYEEIYRSNPWWQAVNPAWLNPAGL
AIDALQRHRRLLDKAESYHKQLAQKFHQPTYAYWGNDAADHKAWGTVTWRAVVANVYDPK
PGDVFFGDAMPTRAWSERPGLPIVGHPAQWRWSPGHDDLPANEGLPERYLLDGKGQTLRCTIQ
PAADAGDGTVPAGQSGAGVMRGSPAVACRTVGYDHQMSYNNDSVRAFVFDSVVRAIEPIVVK
A
>YP_840525
MSDNTNQSGGNSDFSDDTEQVVRQVGRTDKDGASVGHFTLTPASDTRQKELLCDVRPIIPVIFL
PGVMGSPLVNKDTGEDIFFPPNTDGMLGKAGALPALLGMWFRGASTRETLYDPTVAQVTPFGP

IRAGKLQKDDKEDQYVDEAEARRRGWGSVYRSSYQPMLAWLEEQLNEPKYMGKHKGAWIE
TDPDGTEWTLKPVLGTEPADYGATDAGAQKRGQAITEECAEFEHFAKYRYRVYAIGYNWLQS
NEKSAKDVIEGLDVEDKKTGKKMRLMGIKEIIAENHSGKAIILTHSMGGLVARMAIAMHGAAG
LMHGVFHNVLPATGAPIAAKRFRTGGGSEGGINGFINGALLGSDADEFVAVAANAPGPLELLPM
PDYHNGDPWWIFARLDGTPVMKLPQQGDVYNEVYINQKWYGLVPEQSTSLLDPAGIVQKRLD
NQPVKMSLSENFAKTMLFVVRNQEKIKDTYHDKTYVAYGDGALTPKASTASSEGNAKPKMEK
SEKLDDLLAWGTVIWKGNIPPDVTEEELRAARFLGEKHDDSHTGTLRVHLDSRNVTIEFEVQK
VAKLPPGSDAPDPQKNGIVPGDGTVPVWSAEAPAHSAEGGAARGIQMVFDQGGYVHQDSYK
HPWARWALLYSIVQIAKDAPAC
>NP_250201
MSSEPLEPNQDVIIPRSRDSLGRPVYKAQLTRTDNQSEKVALIRQTAPLPVIFIPGIMGTNLRNKA
DKSEVWRPPNGLWPMDDLFASIGALWTWAWRGPKARQELLKAEQVEVDDQGTIDVGQSGLS
EEAARLRGWGKVMRSAYNPVMGLMERRLDNIVSRRELQAWWNDEALSPPGDQGEEQGKVG
PIDEEELLRASRYQFDVWCAGYNWLQSNRQSALDVRDYIENTVLPFYQKECGLDPEQMRRMK
VILVTHSMGGLVARALTQLHGYERVLGVVHGVQPATGSSTIYHHMRCGYEGIAQVVLGRNAG
EVTAIVANSAGALELAPSAEYREGRPWLFLCDAQGQVLKDIDGKPRAYPQNQDPYEEIYKNTT
WYGLVPEQNSQYLDMSDKKEGLRVGPRDNFEDLIDSIANFHGELSAAGYHSETYAHYGADDS
RHSWRDLIWKGDPTPLETPGATLNDDENGTYNSWFRRGLPTIVQGPLETGNPLDASGSGGDET
VPTDSGQAPALAGVKASFRHGSKGKGQANTKRGYEHQESYNDARAQWAALYGVIKITQLAD
WHPNDKGGT
>ZP_09628467
MATDRIHLPIGEEIGGYAGATVALTPSTDKRRISLPVPPDWVIPIIFIPGVMGTHLRMGKKRQADL
DREDNRAWRPDEKLDSLSRRNDPPKRRQLNFDPDETEVDRYQITEDAGKFDMTGEATANSDR
RHGNVPDGLPNIGLLMSAPLPPAAEQWKAKRGKQEATAAQKARWRGWSEVMFESYGTVIKL
LEARMNELLTPTGDVSLGWKMPLSIPVLGVNPREWGCAGDPLTEEELRRVGNCWYPVYAMG
YNWLQSNGVSAGKLATRIDEVIKMYRANGRRCEEVIVVTHSMGGLVARAMLNPKYGKGIGEK
ILGIYHSVQPPIGAGAAYKRVRTGIDDAKGSVPAAIARAVIGKTGKEVTAVFANASGPLELLPTA
SYPRGWLRVQTSEYRQVMALPIASDAPLKAYFDELDLHKKLGTAKPAPPVAVGDPVYDIYARE
PRAWWRLLNPDWVNPADKKYEGADPNKITLERIAETQKFHKNIKDLYHPTTYASYGEDPSQKS
YGTVTYRVNATDLSRFGDPLSWTFESEDGEGRIVVRAMNRQTLQLRLEPPIDAGDQTVPSEAS
ASHVRATMVFRQTGYEHQNSYNDDKVLASTLYSIVKIANTAPWWNK
>ZP_18494315
MSNKPEPIRELECKFDDNGSPSWDSFPSHKNCQVRGGCDLPPHLPGIIILVHGVNSTGEWFSVA
EKKLCEGLNKRLGLTGTSYELETNKYLFDDKIDAVPLMSRDLPDVNINKSPVIRFYWGYASSK
GNEDRYIIPLANKKGVDYHQLKRENMPYANIMAQGPFFWGGGPFQNGTNNLHSLWSEKGFKE
RVGGVKVQWFNEDKDRLLTNAPPRKYYAHAAKRLADMVDSIRNKYPKDTVTIISHSQGTMIA
MAAVAIAKNAPDALFLLNSPYALDHNDLNGASLPADECISPEGRQNTLSAIIDKVACRKNHLLS
LGYEGLCVGQTADKKNWRPDVALVGENGDGLAERDNHGRTYIYFCPHDRVMGSRPLRSIGW
QGLPNDSQGQPHPLLKKHQGYLFQRMLARSTPCGETPNPVTPFAKLPDGKPFWDDKGDKYQS
SSFTYPDPPEWQTVFINAEMVPEPIEAAKLADFDESRVGAEHDDREIDGWGEINPDKKSKNDN
TYDNYINLYPDQDIVTGFKNTGTESEPRMVPVTRKETFEEKDLRLRTYVSQPTDHSTLPMRADF
MSKVVAYDLPIGYCDATWDKEFMADLRRKADWTQGEDPYLFTGIPDNVPEPDIISRETVTDKF
NKEKYKLPMYRSVNKA
>NP_755270

MSTNKSEPTRKVDVHLTDNGTPFAYSMTSHKNVKVRAEVQPPLQLPGLIIFVHGVNSEGEWYD
YAERSLAGLNQRLGLEGEHGLKENNYEGGFFVNSDKSEGGWEHTYEIEGSQKKWVSGPRKIT
KGGDGRSPVIRFYWGYRAADNETDTYAIPLKNKKGDNYYDLPPESRKAKGPWFWGGGPFQN
GCNQLVSLWSKTGFNNNPSLLGVPLPFSTQVLNGERDRLLSDAPPRHYYAHAAGRLAKLIKTIR
NQHPEDTVTVLSHSQGTMIALAAAAIEAPDALFVMNSPYALENEPTTYISYPIKEIISRKARSATF
ADIVKKVAENKTRLKQQGCDNLLAGMSSDGNSWIPEGKTHNGLPERDNHGTTWIYCNPHDRV
MGSSPLRSIGWQGLPDTKDGTPHTLFKQAGDTLYVRILGRNTPCGGTPTAQTHFSNLGDGKPF
WDSTTTLLQRATWPDPDSGQTLTINAPQVPEPLTAEELKNFDQDYARDEKQSGGAGYAYGQIN
PETKKPVDTDYRYYISLYGYFDRKMVPKKDSGYYQSGPGSKEDRVKYEKQSQEEMLEEVRTY
VQRPTDHSTLPSDERFMSRVVAYDLPIGYCWHSWDKAGLEELRRQADWLESDDYYFSGKLTV
PPIPPAIKQDVAEDAEQRKAEEKARLRNV
>YP_934973
MNTSDFIAVREAVLQPDKGTATCVAQLPLPGVIILVHGVNSDGEWYEATEQGLCAGLNTRLGR
RDEDLAIKGVAGGQMVPTTYAPEIDENGFLDRNRDSETFIRDTPFSPVIRFRWGYKANKDELAE
YGSGIWLNEHNYWGGGPFANGCTSLGDLWGAGVDDRLFLWFTVQHLNPETGRQVYACPPRH
YYAFAALRLAELVRSIRQKQADVPITIVCHSQGNMVGLAAAFYGARIGTVTDSEGKSAPAIADN
YVLANAPYSLVEKGMGTDDWAQRYSVNSKGQWGRQTRNAREQTLANFFALIRSRIGSDQPKD
EVQRVCGNGEAYDVATDRSLRCRNGRVTVYCNPHDRVISSLTVQGIGWRGMNAEDFETTQAH
GVLFQRVWAQGNPVGASADGEYRYWDGESAFWHPPPRKARYSLKQGVEAGASIIGKVMTVV
STPLIWLIVLAVRAFDKSPRVNAMPDPEWRVPVNAPVLPEPHLPQGSRLGQPTAFDQDGDPDH
DRLRPRLNDGGSEPDDPYQAYRRDATEVTEAAPQADADTEGRLKYEHRARLRMNDRRRGGD
GEGANQAPGETTKRWDAWARKEIKSFLDQSLDQHATDHSTIMTCAANMEKVLAYDVAVGLSR
LTDKDWRDLRVAADWQLWEGLDRSDAHRYFGEYFDTGRLGHGGGKRLPLHEHPDFNTTLHS
AAIPPGVVDERRHAAESREARNGKPT
>YP_006577256
MADEKKSPYCPRVVSENDIPVPKSLSLTAQTCRVGIPRPMPGIVILVHGVNDVGEAYQNQERGII
AGLNKRLNRSDMYAHEWHDFIMMHNEEAQKKIKAPGRSPVIPFYWGYKPVTHDEYRADQQR
YRNEVSKLKAEAHLPFDAYQEDDAKKKAELGNDGQGAFKYQNDNFGNALDVNYAKGGGTF
ANATTNIPDMLGPGAGGVALAAAGFMTLHANGGDFTHPIFPNPHRIYQFFAAQRLADLILTIRR
EPVTENDVINIVAHSQGTLITMLANMLVKQAGYEPVNCVILNHSPYSLESRLAEDIQPGHHQTS
DARVQTFKNFCALMATHYKGGEITEADILAMEAACALRKPSDNPLRKDERYRRDNNGRVYNY
FCPNDGTVSLKNIQGFGWRGIPEDIASHIPNLYQRVFYQHGEVGAKPDGKTFSLPSARTGDADY
SSIGNASYTAHDVIVNGEELPVKFTFMLQGAGNHKDDDPKTSDKPYTAYIDPDSPDAYISYSAK
AYAIKRTQSATYVVSRYQSLSWRPGHVLTPDELKMESYERGVEVIKGVVTGTKDFPTVTLTWL
RPREALEKEWAKSDPVSYSQHSSIVMSEYAPSHAMAFDLAIGQCRSFDFKAGKFWEELLHRAD
WRDPLNKNQQAVEYYRSGILPDETTKWFMNRPDDILPTGNFGVVNEFNNAITVKPSKDLAAG
NQEIANLQWDMPKTKSDRELGLQNQVSALDLRPYGAGQGSL
>NP_248951
MNDRVRSRTIVSAQSITLPKGGDVHLVPPPPKPCVTIVVHGVNDLAGCYERIERGLCQGLNERL
DMPPTLPGGQANPGYLTPAGYSLPADDEGKAENPDVVYYRRKFASGAGGAAVRSVVVPFYW
GFREEEQYINKTAAHGEWLDRNGNRLDKSGTKEGGQFVNATTNLPDMWGQGFNGKLFGFISL
DWFGGTMTHPLFSAAGRKYMVLAAMRLAMLIKIIRKRYPDDTINVVGHSQGTLLTLLAHAFL
KDDGVAPADGVIMLNSPYGLFEPLNEKLQGWSSQQTREARLATLKGILEFICGRRHPVPALSSV
ALRNCQGYGAIGGPGWVGGQGCQTTIDGERLSFDERDNRGSVYLYFTPQDQTVGLANVQGIG

WRGIAEQVKGLPGRTGLPQGFHQRIFTVRKRNGEKEKIGGHAPPHVYPLLLAGEKTWEDTGL
GGKDRFGRANFDQGDSVLLTAPRLPLPTEARFDFDGAVTAPGENSASGVYQVR
DTLDPIDAAIGVSNGGWKEKDSGHAVAQQVDAALAYRYGRDARSVERALNEGKELAQQTHV
FSARELGTGMVLVTRAETPYEARLRLQTAEGHLEPLSFHSAIPNNPEHNRRVLAYDLAIGAGDS
VDDVVFYQYLCRVADWRLDWKASDKGIFSQGDASVDLPDEEVRALYRAEESKNSQLIDATVA
YRKSGEFPVVVGNRLPSLVGTQTILDRYHEQAVRFGGTI

>YP_443720
MANRPGDRPVPIPRDLPGVVIFIHGVNDPGAAYATVERGLCQGLNERLSRSDLRPAEYGREYAE
AIKAKDRKSPFFDSKIANDPDMYLYRRAESGGAHSMFLPFYWGYRASDNEIAKINHPGEIKSRV
ADSDGNLMTRGQYQDIHGNRLDAHFGKGGGFFANATNNIPQMYSRGFEPDKLERTVMQNAL
AGNTIFAGKSPERRYFVLAAARLANLIKTIRTIQPSALALEHGMDPQHETITVMGHSQGTIITLL
AQAMLKQQGQRCVDCIIMVDTPYSLQFTKDGSQQTGHAKLKTLVDIVNAVTSEPHTIPDLAEL
MIDSAHSCGRAGQNWSQTQGKRLDKSGKHWITFDERDNRGKVYLYFCPEDTVVGLDKVRGI
GTFGVPDEVPADGAAASRGKTMPAMTALEPKRFFQRMWTRLERDQDGRGRRSKVAVGTPPAR
VPVRDPFQRLTPGPDTDGTMLGTLVESGKNMALQASFKRNDIRLINGEQLKPAYEPDLYGGEV
QKGGQVPGHADVAGLMRPDDVTKNVALGNQYAKFQWKDVATTDDPGASIEPHKQAFNRGRP
VDEQSHNWRIVPSRSLGSMLSAAATGGRYQTYVIQREETPDEVRKRMRTDADQLEANNYHSG
VLLSSENHRWVTAMDVAIGQAVTLDDPDWRQLLLLMADWKMTPSAQKKITNCKSFARLDDH
TQGFIDACAKYYQKGLFPSEKFVSLALPSLITSELKLDQKT

>NP_794788
MNGADEYAVAQGNTRLIPNLNTTCKMEVPADLPGVVIFLHGVNDPGASYESVETGLCQGVNE
RLDRPDLVPGRYGGKYKEAGNVPYEKRDSDQKVILDDPDTYLYRRDASDPKTHSLLIPFYWG
HRAAPDQIKRDDAGDPFRMRNQFQDINGNRLDRHFAKAGGFIANATNNIPDVYGEGFRPNLKS
IALETFKPDNALYFGHSPARHYCVLAAHRLAMLIREIRRVSPDETITIMGHSQGTIVTLLAQALL
VDGGDRCADTFIMVDTPYCVLPGNTPKDQDTFSTLVGIVTAITNMPHTQPAMSELRDAKTYCG
RSGSRWLPTQGIRKNKVGSMTVFPERDNRGKVYLYFCPDDTTVSLDDVQGIGTYGMPDALPD
GRMAMMVLPQLRFYQRMWTKRHRYGEAILIGNTPQPELMRATGEARYPGSSFGAGMIARAPI
LEGQERLINAEALSPPHEPEMFGGEASRGTPTTSGLDRPDDVAKGVALGKDEATFMWVRMPSE
YDSPNMSQQEAQNAFNALSDDPENHTRALRKIKSTTNSSSHHEREETPREARERMEKNRDAW
SENSYHSGILRSPENHRWVTAMDIAIGQAKCLDDPAMRDVLIAIADWKIDKKVFEYIEKLPDW
VRLSHKAQALVKASNDYYVKGKFPPSSLVPLTPPPLVGPALNAGTVA

>YP_001915284
MTECSYVVAQANALLLPNRMGERLVEVPADRPGIVIFIHGVNDPGAGYPTVEKGLCQGLNERL
SRIDLRAGQYGVKYAEAKKSPLKPGEQGYKEVASVKYDPDTYLYQRSEDTTSKLPTHSMFIPF
YWGYRASDNEIAKDKRGNPTRLRSQYQDTAGNRLDANFAKAGGFFVNATSNLPDMYGKGFE
TTLKTRGVQMVSPDFTYFGNAPPRRYFVLAAERLAMLVSEIRRLAPDDTITIMGHSQGTMITLL
AQAMLADRRQRCADCLILVDSPYSLLEPKGEEQTTQAKLQTLIKIVKAVTAQPYTRPALNELQV
GQPGYGGRTGHGWTPSQGTRLDAEGKQIVFAERDNRGKVYLYFCPQDTTVALDQVQGIGTYG
VPDTVHVAWKRKFYSTERTASLPAMDALKDMRFHQRMWTKLLRGGKPVAVGLPPQHIPLRME
DEARYPGGGVGPTTTASQTPLPQEGRYINGEALQPPHAPQMEDGEADARQYKSSTPLRGTPTR
AGKDAPDDVSVDVALGNPKASLNQYRVFERFVDKNLSDPDLQELTQQFNANHPDLNDQTPGY
DCENGDDMGYMLWRHATPNEVRAQMAHNPAALVDNSYHSAMLRSTENHRWVTAMDVAIGQ
AQTLDDPEWRKVLIAFANWRTPFKPSESQLPGQLTLLELANFEKLSPGAQMLAQQTAYYYTTG
KFPDGVSKEPPDKYVISRTRAQRAESNE

>ZP_05828070
MTQAIQNETRSVRDMLIAAKVLAKDGKATESPCKTCRVPVWVSFFFDGTGNNKDADAATLNQ
SNVVALFEAHKQDSKNGIEKFYYEGLGTQFRFDKYSVVDSGKITAAARSLQGRKIDITDAEWR
KQGYSESGKGVQGALGLGVALGIKQRLQKAIFELVDYLDKIYTQKGITEINISAFGFSRGATEAR
IFMNWLQHAPNVTTQGTGSGKKLFYRGKPLKAKFLGIFDTVESIGNAAQNKNPELYRTRIEDYI
EHSMHLVASLEMRQSFPLTPTGKPTANTVKGLIHDQKVYPGVHSNVGGGYMPMEQARILGLS
RITLHAMYNRACAYGLKFFTLNELNAAKQRKIVFTRFYAFDSKWQQDLNNFMAYVKGGTSFE
QQMQGQIALYHQWIREGGYARFIHRKTRERIGRKEKITAITKLNDGLFENIRQALNVYVPEGAR
PYDVIKGRDRKSTLPKEVIYYFENYVCDSVGGFIAEASDFQAILNDGKAPNYFIPRGIVRPT
>NP_899682
MQDWSDSSFETQMQAAQKRQELHPLLAGCPTCEQKPWISVFFDGTGNNGEIDADKKKWSNIY
RLFQGHADDQTRGIFPIYIPGPGTPLSVSNAGWLSKLRDSGALGGGFGLGMDARMDKAFKLFS
RNLADCQRVSRIDIAIFGFSRGATLARAWINLLLKECIWEKGKPHWRMLNAKNGVSAEICIRYV
GLFDTVESVGMVAKNWSPSQCMTLPNVVERCVHYVSAHELRGAFPLTTVADTAGAPPGEERV
WPGMHSDVGGGYRPNEQGRFDTLSRLPLNAMRLDAYLAGVPFLSPAELKGNMVENKHVFDY
FEYDAELKNLYDHYKSQISDASSDLDRTIISHMKLYYGWMKLRQDGDIDSLYGKVREERADLA
KELENELSDQRKYLRKIPFENLSRKENVEWARLKISDFRKYREKLESARETYGKNFRPYELNSR
QLTYWDAWEKSVVPDEKITKFFDFYVHDSRAGFTFNSGDYLEPREVLERKVCSVEPARRGKQI
AASNIGVDHQ
>YP_003748701
MSAFRPGWYEVGFDAATRDRHCRYFKSPSAYTAECLVKSTGQQILLWSDPAGPDGVGQPAQW
QPAYRPLAGATTDDWWNHPPGGANYRPELPPSGSRTQRKPTLKELCERAATADFAGKRSLSCS
REIHVGLFFDGTNNNMVRDTPSQSHTNVVVLFNAHRDDRQDHFAFYVPGVGTKFPEIGEAEEL
DAGKTYAAGGEARIHWGMLQVFNAVHTALCGTDLLDQKEMKHMVTSTAGLSTWWRVGDEK
MVRTFGDLQKRLLKAIDGKRPRVTKVHLSVFGFSRGAAQARTCCQWIRKATGMRVGEAALN
MRFLGIFDTVASVGLADSSPVGQGFLDWADGTMDIGDFERVVHFVAAHEIRQSFPLSTARIGSK
HYPPNTKEFVYPGAHSDVGGGYAPGAQGKATGGRSELLSQIPLLDMYVEARNAGVKLLGLNE
MRPEVQADFVVAPTLDKAFSDYSAWTGRMEKQDVAVSNGPPIQNRMQYHTQLYWRWRAHIS
SDAAFKALSSYTHASTQDKTDLFESELDWRRDVSRAQEASKTRHVARGRGAYVAIPPPATELQ
KQIVAQVNAATQVTPGASAFFDKHVHDSHAGFWLLGPITATQRLAFITNVKAKKAKYDALLRR
AQTPGNPHADQQRSAALAYELNGFERRVLASDASSAGSVPVMSDADAADLRANAGIATSAAL
WILGTEKRREPHGHGRYRRIFDHG
>ZP_05133435
MPANGQRALSGAEAQQRARAMACLREKGSECQGQVHVSIYFDGTGNNREWEGTFVTGKTRS
PKTQLARNGHSNVARLYDAALKERENGFFSIYVPGVGTPFADVGDTNQDGDTLGGGAARYGA
DRIHWAILQIINSVHQYLNASDLIQPNEMKVLVASMSETRLLEGMARRSMLTAIAQRLERVVNG
HQRRVKSVHVSVFGFSRGAAQARAFVHRLYETAEAWGSGCGYNIAGIPLYLNFMGIFDTVASV
GVAAMSRVSKGKWDWAAGDMMSIHPEARQCVHFAALHEQRINFPLDLATSGREVLYPGMHS
DVGGGYSPGGQGKDFVSGSADGKAKLSQIPLIDMHHEAVKAGVIVKTIDEIGTRPTLAMHFGC
HPQLIRDYNAWLTGHGVPGGGHAQQIAGHCRQYVAWKGKRLPNGPQSVLQQPFFTQSDAED
QVDLANAQRDFANLVGKLSRGKQEMAAYRSQLDESQKRMEAGRKAGRPVFEPTPRASQAAY
DYAGIPAETSTLLNLVLDGAPVPDVSTNLFDNYMHDSLAGFYIGKWTELNIPAVSTYGYLRYRE
VFSVAGRRAQECRDPATLPPANIPSIGGAFQQLGTAMGG
>YP_005627674

MQLSRAQQAEMMSAGLGRPQDSCCIDLRWGFFFDGTNNNFHRDQPKKAHSNVARLYDIFEAD
RRKPEFVRRYAAGVGTPFKDEVGDQGLGIQEKAGLAAGWGGEARICWALLKFLDNLNYYFER
IDLGEALGQSDPATVRRMAQDITIPSMELRKIAGDETEMLRQISMMASLQSLTATALNLPNHRG
RRAVLAERRAQLRQRVQTWQRAQPKPKLRSIRVSVFGFSRGAAEARVFCSWLKDACDGGGGE
LTLCGIPVQLDLLGIFDTVASVGLANSSRLWSGHGGYASEDDLQIAPYVRRCVHLVAAHEVRGS
FPLDAAAGVNGEEVVYPGVHSDVGGGYEPGEQGKAFIGDSIDDSAKLSQIALCHMYREAMAA
GVPLNLSASRLSKETKAAFKVDKGLIDAFNGYVAATGSIKASTTVALTQAHYALYLRWRRLRL
DDTAPDGMAQQPFVTRARTYKAQDVTDLLQTNAELRQEWAALQQDEKDAAYSSEASVAHVL
RSTLAPIAARDDIVALVWGEKMTQWREVKPAWNDLSPLDRRIVRLHDDYSHDSRAWFKPFGA
ASEEAWKRQYRQRMNRLEAQDNAWQQWNRDVQPVIDDAVRKAQKHPGSFQPTPEVRPMPP
LVAGQDLKDLKQWRSNGGVIPTEQDGRESYGMFGFLRWRTIFVPEKSALAHSIDAVDETLEQI
KQLPGKAKQAVGDAVESAVDSAVEAGKDFVGDQVRKLIPSGLPRM
>YP_443213
MNFRFAPAERPDIGVVTKEEKDAILRRLHDDDGMSCCKTLHIGIFFDGTRNNAERDKSGHKHS
NVARLRDAFPQDRYHKSIYVAGVGTPFSSEIGDYGIGLQAVAGASAGWAGEGRINWALLQIHN
AVHECAFRVGLSTALGVDDKNLVKLMSLDMNFKGIDLGGNAPQPGSTGDIKSRSSPGIGALKLI
AAEQYGAELTWDKDTNWSQLKDDLDSSKWAAAVRAWDGRRRKILGDRRAQLKARVGDMLV
KGKPRIQRIRLYVFGFSRGAAEARTFSNWLVDALESDFSLCGVPVSYDFLGIFDTVASVGIAQSA
AATLFDGHGGWARKELMAVPHYVRRCVHMVAAHEPRGSFPLDLIDCSLEGREEIVYPGVHSD
VGGGYGPAEQGRGRGDADKLSQVPLVDMYRAARIAGVPLDIQGPGITSEAADVFKISAGLKQ
AFTAYVKASEGYYYAKEHGTAGLMRAHYGLYLRWRRMRLKDMSLQPSFKAAQANCPQDAM
DIDSANKELRAEWEDLLEIEKEGGPSVAHYAKKFGAKVARDNPKIVASVSAVLLPGVIVFSTRP
EVIYGVRKAGDRVTELVRAQLQEKWEQWQQVRSDWNMGPPEAPISALYDNYMHDSRAWFK
PLGDDDDVWNYKQIQELKSKQASFEREHAAWRKRAETGAPGPWQIAQAMSAGASGLGPIAM
QPEPEPRSPLTAQQADLLKRYDAAMQSAKQARAAKDPNAPTDSAVLTDPKVTGGLALQTSGR
EFYFLWGFLRWRTVFVNGVRWDQPRVPTVQEEMEGMRMQMQRQVDMKGIGVLFQ
>NP_251980
MPNFGFHIAPTHPVAGRLTYDSKKLSENILKQQSDERVFSRAQEQKRLSEGDVVGGAPCCKAIH
ITLGFDGTNNNDKADGSSVSPSCSNVARLIHASIGSGDDINSRGIFKYYCPGVGTVFPDIKEFTPS
NMGLIGAEGGENRINWGLVQLVDALFYTLLKSRLKLNDVQGLVEEMSTNWTVSTLTGGLLEN
GEKKRRAALEPKLKELEEKLRQRQNSGQKPHILAMRLYIYGFSRGAAEARAFANWLQELTRVS
DADGRVEYRFAGLPISIEFLGLFDTVAAVGLADSAPFAAGHMDWADDTMRLPDEALSQCLPTIL
PEDCSFLKRCVHLVSCHEQRASFPLDSIRRRDMDANGRRTGPSCYRKWTVEYAYPGVHSDVG
GGYGVGNQGKAVGGSEFLLSQIALQHMYAEAFEAGAPLQVPAPAVHPDFHEEWRVMVPKIEA
EFSVSEELATRFNAWQAQAKAGPLEEVIRRETALITAWRIDRYAGGLRNKAFFANVPPDMPEAQ
QKAWEALHKRRSREYAAAQQGEPLPPMSAAEQAEWDRNVALIGGEDQLRDLRVEKQFDPPLD
QRQLLGAAAEFAHDYKGDWGVLDDGMTVGGVIDLLLGGTVFLINEEDEAEEYSQIHRDGSAR
YHQLFSAPDRVAPGQEKLVALFDEQVHDSRAWFMNTSAIGPREPFTDYFRYRLVHFDNESNKR
LSVLATAGRVVGVGVMLASVGLSVKRRDPRMLLGLFLPSLARPLLSGKVGLPEISAFDPLTGIA
LPMVGGAALDNLRAFTREPGDKVEQIGQLPPPPPLAVAAVQSPALQQVLLAQQTVEALKARDL
GSLAGLVAKAELTQAPAAATPAWLAEAKQALQDMGTEQAQPPGPGSAPGWLKRGKDLMESL
>YP_004594328
MSDTAESTLNALKALIAAFQTGATPVAVKTSTAEAASLPPKFPGPDDEKEKTPADNSTQQKAEY
NERGRLPASRTQTEGNYARQYLEGYVGESDHQSDKKTEPGCPASLHISLFFDGTCCTKEGADEI

YGSRNPPLTNIGRLYHAANWKEQDETAENDGYFSYYFPGCGARFPEIGEEHYSLDGELFANGG
EDRINQALLKTYSSISYAVNKTAIKDSELTRYRNSMATVWPFSRLTQKFDRKSALDKFCDDYLG
CVVNQWPRQPTRLHIQRSQRRIAKIKLFVYGYCRGAATARAFARGLESLLDDAKMPLDATNM
LPAGTVVPGGPTLQGIPISIEFMGLLDTVSAVGVPHILPSATGHLGWAANSLRLPTTKGFLKSCY
HFVAGHEQHGDLPLDSIRGPDGKYPAGVVEVVYPGVHADVGGDCKPSELGKVQTDARSLLSK
IVLHDMYAAAFDAGAPLSVPRDVLPGGAKNKTYRVMQGDVADQFKISAQAIALFNAWQQAG
AKTETPAVNEAEVVAIAVAQGRDPVAALEAARKKADGDKAKAAATAAKSPENGPLGKLPDPV
YVPLKAKALEVMLADQLSWITAWRTERFASPQYVDKYYQRKLFYQQALNESSGALPSATAPV
ESEVPREVPFIDKDRLDRAAWGYDFAHTGIVALLGQVLLDVIPTTQTDQLGGPLVAAQESEYIQI
KKSGDARRAALMGSPTMLDFYDNYVHDTLAKYNHDPLHSSFSHGYFASRTIYDNDDDTWDS
VKQFGQRVKAIIDINVGDILVQARTDMKIQLKIFIAANSI
>YP_002396321
MSSKISGLAFPCYAPPVFPEDGRLILSEAQVNANYLKQINKTEEHKTNCSKQAGFRIGFTCNQSL
HISLFFDGTNNNEYNDTPGHPTNIAKLFHTTYQNAEEQGYFNYYIPGVGTPFPKIGEMDYSNSG
LEFATGGEDRINWALLRLVDALSYSIDPSHKRLDDNVAKEHIPLMRAPWPMTGEVNRRNVINP
YLEKLQGLLEQASPRLLNVKLFIYGFSRGAAEARTFVNWLTQLACPQEQQVMLAGLAVSIEFL
GLLDTVASVGAAHVLPGAAGHMGWADSTQQLPDERQFPGLIKCCRHFVAAHEQRLCFPLDSIR
RPDGNYPLNAEEIIYPGMHSDVGGGYPVRDQGKSCGDSGDILSQIALHDMYLAAFDSGAPLAV
YSKFVTPLIKGVSPLRIMSPSSVKEFTIANSLTKRFNIWRQTLLNTTLQGTEEMIDTREGYHPYQ
LAQVLQQIKAMGDGLYPQFFIDSLGTGTRTQPCAELLALYDDQVHDSRAWFMQSSLGGREPW
GGYFRYRMIYFGDEANKELKLISADGEVVGDQPTSNRVIYWMESKTVSRG
>YP_001478026
MSEINTDLAWLPPAFPAQGRLPTQAALVGANCAQQDSHELAYRQALCLAAGRRVEPPCCKTLH
VSLFFDGTGNNLNHDLYIADPKHPTNIARLFRATIGQGAAGGVSKGPELLDADGSGEDKYYKY
YIPGVGTPFPEVNDLDFTMMGLAVATHGEDRINWGLLRLLDALKRTMTRKSLSDDESWKAVD
KMATSMASFGLTGSANRFETFQRLLKDMSPDLQKALMPAEPGKPKLLGIKLYVYGFSRGAAA
ARAFVRWLSELLPKPEEGQDRPEQYLAVGGLKIPLSVEFLGLLDTVASVGVAHVAPVAEGHMG
WADGTQELPAEKIYGGLIKKCVHLVSSHEQRLCFPLDSIRRPDGTYPANSQEVVYPGMHSDLG
GGYPPGDQGKANDEFDRFLLSQLALHDLYASAFNSGAPLKVSPPSLPTDLQKDIWRQMSPELQ
LEFAVAPELINRFNAWRELTLGLTVPLQPLSPEQAAKYDPPRAPVSLEKAVENQLGWITAWRINR
YAGGSYKTQRFYVDSAANGLDKDSDPLVRKQSEAARKALQKEVDDVRRDMKAQHNPEEGFL
KLPPGPKDFDAALGQTQLRQAAEEFREDYHGLSRTSTGNWLFTVADSVNNAIFLLNNDDEYG
EWLRIKTAGDDRVKVLFPISGDASSATQSAGLVRALFDDQIHDSRAWFMHNAFGSREPWGSYF
LYRMIYFGSRSSKPMTPLMIAGAVVGVATLAAGVAVIIKQKSAKGKLAGLVGTAGAIYLETQAV
DLLSGKPLPMLPNAAQLQAPTMEPGVVVAQQTQAVAEQRLALAKSLIESGWAERLKSTVTA
>YP_005954437
MSEITENHAAWVPPPFPPQGRLPGRALQVGQNCHQQNSDERRYHQELCLAAGRRVEPPCCKT
LHISLFFDGTGNNLNHDFFIANPKHPTNIARLFRATIGTGTAGGVPSDDQSKLFDDDGGGDGKY
FKFYMPGVGTPFPEVNDPDYSTMGLVGAFKGEDRINWALLRIIDVLMFSATEKWLTTTESRRSL
KEMSTSWNQLGFGGSHNRYEEFTRLLNDLASDLKPLIIQPEPGKPKLTGIKLYVYGFSRGAAAA
RTFVRWLSELLPPPAAEGEKPPQCLQTGGMQLPVSVEFLGLLDTVASVGVAHVVPVADGHMS
WADGTMELPDDETYGGLIKKCVHLVSGHEQRLCFPLDSVRRANGKYPPCATEVVYPGMHSDI
GGGYPPGDQGKANGENDSLLLSQVVLNDLYSASFQAGAPLKVPVDTLPVDLKKDAWRAMHP
DLIKQFDTDIPLVNRFNAWRELTLGQTTPKTFDPEAASHYEPPAAGGSLETVIAEQMAWITAWRI

DRYARGSMLKTPFYQRAKNTDALPAARKAAEEVRDEKQAAVLRARQNQIANQPPDRMDELV
LQPGVKDFDPKMDQTQLFDAAKEFGKDYHDGYRIPDNLAQLVLDTVLQPVIFILNTDDEAQE
YRRMKRDGEARVAVLFPEAGEASNAEQPAGLVRALFDDQVHDSRAWFMYAALGTREMWTGY
FRYRMIYFSERCSKPLSPLVLAGDLVGFATVTAGVVLSFRQKRLTGKLAGLAATGAVRSLEVAV
LDQITGEALPELPGGEQLRAFTHEPGTVVAQQKARKADEQLARGQAALPASWLEDVLTTTV
>YP_002007700
MGAPDKQVYQIVGGDQKAAKTATSAPGCFVHGKDVFSEPVKGNAVQFFVTGTEYFDNVARAI
EGAQSSVFITGWQVNFDVVLTGKKTLWNCLRTAVRNGASVYVMPWMSPKVGVDTGDLETAL
TVIQLNAGLPSPRAFVLPAVSQCDQPGALGIAFSHHQKLVVIDNKFAYVGGIDLAYGRRDDGKY
SLKAEGRQGSEFYNSCVPAIHSLSSVEQTAYLTRAELVAACFDNKAGRAAQFFLSAPMKPLAGA
MDAYSSASDKIKDVNKQISDWWVTSDVVPEFVRKAQDKVIDAAQETAADASKWAYQQLGTT
LQTKVEKLREYGGAQVADATTALMAWLNGATLDSLPPSLLQNTADTIQAFVMRLVLALQAEG
SQRKQCYANLEKLGKLLPAGGKCPDSSVQPRMPWHDVHCRIEGPSVYDLSRNFVRRWNGVAL
QYERSDGKTVDALLRHLGIAARLKAPRIGAAHRPVRSKAQPGSCWVQVLRSAPKKMRVAEAA
GEADKTTPSVAESSCLSAMLKNIEGASHFIYIEGQFFQSDYGSTMIGNEEADGGAPVSGPMHAL
MDVKGSPGYQKYAAQLGILGVPPGQIYKSLKWSQIDDVQRDIRGGGADFVNDLKRVMATQA
QIAGFSALGPSQKSLKNPICKALGDRITRAIYDGKPFHVYMVLPVHPEGTLDTINIMTQQHLTM
QSLVFGSHSLVNRIRRALLAMKYVRERKMEVKRARDAAESAKIEDLNRNIRQGEWQKYLTLL
NLRNWDVLHGRPVTEQIYVHSKLLIADDRVVVLGSANINDRSQLGDRDSELAVVIHDDKAMS
VRLDGQLVQPVGTFAHGLRKALWKKHFGLMGGVAPATELGLPSILDGPGDLDTWQAVQRVAQ
TNAEAYDKAFPYVPRSKSRSSVWPTWDDSNGKLRGYMPFNERFWREERPRDDSFTWDAKQIL
SESTPSGVRGFIVALPVMWTLGENNDSKMNLTTLAHVESEDEVNRERQTASRSPSQSGQADAY
S
>NP_252177
MLQKKPYNGLHEKELNQINQQDGSPCVAISAPGCFIKGSNLFSEKRAGNRVRFFTTGRDYFSDL
ASALDSASSSIFITGWQVNYDVLLDGRRSLWQCLRQALERSPALKVYVMPWLSPSGSLGTYDF
ETMLAVFQLNAGLEGGARAFCTPAIQQSDMQGLGVAFSHHQKSVVIDNRIGYVGGIDLAYGRR
DDNDFSLDASGRRGNDAYNPGLPHLGWMAEDEHVSSMGLMMATLFDLSRPLASLTLHAPTLR
LSPFPHIAASDEPLLSIPLAPSRARALNGAAYLSDLFRSPMLPSLQWLGRAYNSSKEGLDEGFER
LDALRRQMVASSIRAIANLIADNLDALPIEPELERRLRAWLEELRTAALNLPEALRIKSLLLINQ
WMSETELGQVLTLISGKGFEDIPQNLSGKAGELAGSLFWTLHRLMQARAGGHQQPYRYLDEA
PQPLASPDNARLAADQPRMPWQDVHCRIEGPSVYDLARNFIDRWNGQQAYLAKTPALQDTAL
VRSALEAVMKWLNSLAAAAGLENYLDEKRNLRLELDPPTPCWINAPEQLPQEPEVRRGGMTV
QVLRSAAARMLEQEQAGRLGAGVNLPLQVGVSTEGVQSNCKDAMLLAISGAQQFIYIENQFF
QSEFGKEGEVFKDLPLSGPMASLRDVGSLRRDFVVRIRLEEALEQRDLWLLDWAEVEKIAQEP
GTEARQFLKSMLAMWGVNAQGWLTHKLGEAQHGLLNEIGEALARRIERAIQREHPFHVYLVL
PVHPEGALNVPNIMHQVHLTQQSLVFGEQSLVKRIQRQMALKALEGKSDPAQAREIIERKDAR
GRPVYEQQDWSRYLTLLNLRTWAVLGGRVVTEQIYVHSKLLIADDRVAILGSANINDRSLQGER
DSELAVMVRDSEPLTVRLDGKNDAIVGKAIHQLRVNLWKKHFGLSQGPGGFVKPASELSAYLS
IPAAQEAWEAIQTLAKENTRAYERTFNFIPQNISQTQLQLTPEPPKGFEDGFPASIWPTWAYRKPG
ELRAGGQLMEPMPYQEIFWRSSNLTSVKTFPPPNGVSGFITALPTSWTRGERNDSGLNLSILAH
QDSRSLPTQVAMNGDSSAQGKHRT
>ZP_05133335
MADGTKLGSVPHTALTCLDGQKGTSSGDYYAPPDRQFAPVRRGNKVDAYTDGRSAMKAMAD

AIRGAQKFIFIADWQMNFDTELDSRGGAHASRLSELLFDAINQRGVDVRVLLYDSVEAAAYTH
ENEARTALYKMQDANTPGQVQVGLHNPATGRTDAFNIAFSHHQKILVVDGKIGFVGGLDIAHG
RWDDGNFDVVCDPTLHVLNDHYNNCLSKFRGMTRAEQDLTLDKPDTSPDAVGRVRPGFAQA
YVPGLAIALDRMKAQWDAGAALAELQDYADKLGVPDALEREGIRKLAEMVIPGMEEAVKVQ
RSVAKTFLAIQGWLTEIEKEYADVIAASNRAADEAVKLNVGEAAAAAGRAVEGFTKIQAKKFG
EWVDERKAAIKDAILGPIKTATTVVGYVRDPDSLVTDAKAKYAEIENKFNKAKQAWESLVKWI
NEPVDRIQRLLDSGRQPRMPWQDVHARLQGPAVFDICRNFMHRWNAMVWQNQRGDREIGTA
VRKVLNRGIRGANDWTGAGLPEQTTLARGMELTPLSDQWLQAMGGMQALFGDLARPGTAG
DVTVQIVRSSGTALHALEKKGCKELGLNLDDASCLQPYWERNQPMHSILDAMVNCIASARAF
VYLETQFLISECGWSDAEAGRTVETAIKGGERRPMTQAEKEKAGYVDKGVAMSGPIGKTGVV
QDLVKRKQGVKSAASNPLVAAIAARIRRAIVAGQGFHVYITLPVHPEGSLFDGAVLKQQYWVQ
QTLLRGDDSLIRRICRSLIARDKDIREASVEEADLQAEVKAGRWKEYLSVMNLRSYGVLADIN
HKNHFAPHTIGADQATPLYVVTEQCYVHSKLLIVDDAVAIIGSANCNDRSLLGTGDTEIAAVIVD
GAAKSMDLGNGVQVITRKFARDLRMKLWRKFLGEEIEDVPGKLQGYTAVEGNPNHPPFRKRS
SLVDVGKPASGATWKKVQGIAELNASLYQRVFPNVPRDAMSRYDAVFSGFPSAGLVDDGSKV
GRMLYAQPSDLSPEFMREVSEVVNVRTLKVGRHNVEKVMSYLGPSNLEGGLKGFWVAMPLM
WGHGMDDPAALMPTEIIANVLVPAGPASGTLMASRQGGNDEGHQA

>ZP_19154792

MEQCSNHSGRINNLKTLKLDDGMNQCSVYFSDTSEESYTATATHSHWVHYTSGPERQSENFAE
YTAGNAVQAFLGGKAYFAALLKTLTQAKKCLYITGWQINWDAQLAPGIRLVDALLEAAKNNA
GLQIYIMPWKNPGQVETYATATERVFAAMNTYLGRQVFYIQCAKSQSEIFFSHHQKCVIIDENV
AFVGGIDLAYGRYDDHYGLHAEADGRQGMNRYNSCIPPVSHSTGYSPGEEYVVPDSIYSHPER
YEEQKQAEKKEAESVQHLIDEVLKHQLWQSQGSAKDSTYLDPEIQPRMPWQDYQVQIEGPAV
EDLVKNFVLRWNSYSRHCPKNPFQTFIPDLGLPVTCSDKKGTCQVQVLRSASLNMRLAEYKN
MPEAVPEPRLKQDDILRSIHLLISKAEHYIYIENQFFVSDFKNSSILPESELSPVASKLHPKFDAWA
TRLLPDDETPQNPVAEWLGDRIKRAILSHMKQAFHLYIMLPVHPEGRLDDPAVVAQIHLTRQSLI
FGSHSLLNRIRRSLWVRQQLEAQGIPRKEWWRKAAELEAQCGMAFTHIPLEACNEYVTLLNL
RDHAELNGTAVTEQIYVHSKLMIVDDRYVLVGSANINDRSLSGERDSELAVMISDTEHGFTDLD
GSGTAVPFRKFARDLRQKAWRKWMGSAAAECAEVLDKPALKTGWEKIQEIAQENARVYDKIF
EFIPKNYIYQPVDRNYANVGVTSQAESKKSQPLSGRLWP

>ZP_08684414

MPNDSKTQSKSKPKNGQMPITQKKSTVAESWFVGKGGNKNVPSAEQATFLSLVNGEVAFGEL
YDSIEKAKKSVEIVCWGFQPSMYFKRGVNLAGAPIGELLMEKFIQNNVDVKILCWQTNPFATE
KFLEDNTPGRSTLLFMHEYLAYEHFLDFILEPDNIYSHFEKLEKEKFLEPKVAMHKMGSRERHK
FYRGKRSHSVEQRRFDWAWYDAVEGYNTITSDEEFHKTMEEFQSLHDEFHSLGMGAVGFVLR
VKKSVGTGIDEFKEFFDTHLLPDWIKELRDGWRNMRFGIREEIHFRLMNYVHTAAQGLYEEYR
DGKLVKTAIGVVSKVTGLNSNGPVNDVDDLRIALFDKLFAKVKEEVKGSEPVHLIDQVKGSVT
SRLEKLGGLKDDLESIWDLFFNFNLFRNYVNCMNYYFLLRNEKKYPNTMAFMRLRNQEDYK
NRKQKVSFLPREINPAIFPKDFPFEDKGLSALTKDVLRFTPTHHQKSVMIDYTDPKSAVGFVMG
HNMLDRYWDTSDHYALPDEYKTYTTSRKGPGEVVELEKLENIEESQMRGRDPSAGAFFGTPR
QDISCKLTGKVLYDIDANFVQGWNKAIEDNGYYQNRMDKIEPVERVKREQFKPQNVEKDDSQ
TELLYAQVLRTQPEYQEENILALYNQNFKMATSYIYFENQYFRFPPFAEALRDSCKERKNFAERF
GIDASGMQPLCVFVVTNSSKEGLGPGMINTERMLASLGRRDVMPTVARERILEQAGYGNAIQR
MFPNLSADSIWRKILPDFILPPPPLKPIEGLNDREVKERELAEKVKKTKNTEELRKILKEDLEKE

GIKVHICTLVAEDWEEIYIHSKLCLINDTFATLGSANINTRSMQIDSELNVAVESQPASYKIRHET
WGWHTNGNTEMNPGEQLIDPSLAGDVFDTWYEELDNNKGAKDKNKKRLMPLLEFDLTNKN
RVYTQDLD
>ZP_16295898
MATPNNTFTTPLALNHTSNATATLPWFLQFTEYAAKQATFRPLVNGKEAFGTLYDVLMKAQHT
IDIICWGFQPSMYFKRDGHSLRIGELLEQKGQKGVKVRLLCWRDPLYLTEFGENNMPGYDLVT
GLKQHISDDTYRRSSMLSRDYQTDEERKFDVEWYWRADLNNVTSTSLLSPLNPLAKVAYEKA
RAEYYKDHALKNIDFATRGFSPANRAEIAWRTFWHGEDKDRDLQTKAMNSVGMGAGIPTHHQ
KMVLVDYEDPENAVGFVMGHNMLDQYWDTDDHSYKPKTPSTGRNGPSPWQDISSYVTGPVL
QYLNANFCQAWDDATGQGLGKARDGLKDRLKVRYAPGGDTPMMAQIARTQSQKGRRDIEKL
YLQAVNNATQHVFIQNQYFRWPPLANAIKAVAAKHVSWGRNVTVHGPLYLFAITNSGDEAVGT
GTVNTYRMLNALGKAQSIPGVATLEQEDARQTALKDQMAAVVDQQSQANQALLGALEVQGI
VDSPDSAKRVADAKARIEQLKQRRESIASQMKSAPQPVLNKEYDGLKVHVCTLVAPDSPPGKW
VPVYVHAKLMTIDDVFTTLGSANINTRSMEADSELNICHENGDVTKRLRRQLWSLHTNKEGA
QDDPGQAFNKWEMILNNNADLQGGGKHSPVASIIRFMRMSNERTYKD
>YP_003747859
MATQTFTTPLALSCTRSATITLPWFVQRTEYNPAQATFCPLVNGEEAFGAVYDAIAAAKHTIDII
CWGFQPSMYFKRGLDARGTLPIGELLEAKGKQGVKVRLLVWSDSLNVAQFSENMTPGNNPAS
YLSDTRNSTQRELDQLWYWRANLNNATKGWAGKWLMPGGAMDEIAKAIRNHALRDKAMT
NVEFATRDFTLRQRAEIAWRTRTQGKDTERSAFTKDANAAAMAAEPSHHQKMVLIDYETPAQ
AIGFVMGHNTLDAYWDRDDHGYTRMHPQMGRNDKHPRQDISSRVTGPILHYLNENFCQAWD
DATGRSLEAARKGLADEYKCQLKLRRDFDTPVMAQLLRTQSQKDKRDIERMYLQAVNNTTKF
VYIENQYFRFPPLAEKIKEAAKVQFGEGRDQGKHGALHLFVVTNANDDGIGMGTVNTYRMLE
ALGRADTMPGVATLEREDARQASLSQQRAQAMDQQNQANQVIEDANAFLKTEDTASTRQWR
ADAEQKLKQATAKRAELEAEIKREPQKTIQAMKIDGLNVHICTLVAPDSPPGKWDYVYVHAKL
MIVDDVFMTLGSANVNTRSMQVDSELNICHEHSGVTQPLRKKLWGIHSRPSPSERPKVSAKNR
VYAMAVGDDIAEAFDAWGQIIVQNVQNQNKNLAPCASLVGFMRTSEKRSYLD
>ZP_16673047
MYFKRSENRSDLNIGDLLVQKGRDNVKVRILCWHLPPAQFLENPTPGRNIVSWFSKGKQNRN
DSQEDYDKQWYYQAKLTGVADENKALESSELYLKNVEFATRDFGFIERVEIVWQLLVNGEDK
GGTKLNRFMRTSAMSAAPTHHQKMVLIDYEKPEKAIGFVMGHNTLDAYWDRDDHSYARMH
AQFGRNGSTPRQDISSKVSGPILEHLNHNFCQAWKLQTGVDLMLSRKKYAAQLRTQPGAGKPI
MAQLLRTQNQEGVHDIKQLYLKATNNVTNYIYLENQYFRWEPIAKAIKNSAQKQIQAGRSPSK
HGSIYLFVVTNSSDEGMGDGSMNTYRMLDSLGRKDVLPTVSKLERNEALDRQLKEARLRTIN
AKQSLSTLNSINENLIDSKVLAKLKAEREAEILSAEEQQKKLEVAIPLKKGMAILPMEIPGLKVH
VCTLVAPDSPPEDWMPVYIHSKLTIIDDVFTTLGSANINERSMEVDSELNICHEEPALSQPLRKQ
LWNIHTKGLGAQDDVSEAFRMWEDIISENKARQEQNRVPTIEYEKKSPYASLIEFYRDSPARKN
LD
>NP_253776
MSDLYKPQTLKLYAQQAGSVRLTLDWFANKAFYPPRAGVHIKPLINGQAAFDAVHAAMEAAR
HSIDIITWGFDPAMRFKRPDGPRIGELLQTKGREGVQARVLVWSNQLARLKENTIPGAGVGGS
GGTWVGSGVASGSAVDNEVLRLEQRRQHNLNLIARQQEALERSERLHREGRLPSFDPRGAAH
ARTRIAELEAENAEIQRTLDSSGAQGYGGKRGSGGTRQDPWGQIFTRDWFKAVRGGGLQNVE
FRTRDFEQTARPVMNGEQVRLVNGRLQSLIHLLRADGIDDLGIGQLLVLTQFASHHQKMVLVD

YGSPQAIGFVMGHNMHRNYWDTSAHLFDDRAAGRDPGFGPWQDISMQVQGPVLADLSRNFS
EAWDLETPWYKRWFSTPSLTAERDALPLPKIATPASNSVAQICRTQPQDDERSILEHYLKALGN
ATDYVYMENQYFRYAGFAERLRKTAQVRKARGVPGDLYLFVVTNTPDSSDASKTTYDMMKG
LGQEQLMPQVQRDLAHDLREKREQLKQVRENLHPDPYVRRGQENNIERLERKIEALEEKGVT
PEVEQRLGDLGAQEIPGLAKNTGEDDKPYQLVEVPGLKVVVATLATSDPAPGSPPPARLSAEAE
AALGAPPLKARYKHIYVHSKLLLVDDLYTLLSSANINVRSMHGDSELGVAQPNPDLARAMREE
LWELHAQKVATTTEKNFKLWNQKMDANWRQQRKDEPLVANLLRFWDVVTPYSPGLTVD
>YP_003006804
MSQNKQQISTTEGKLCATVNFNWFLKDAEKFENGTQSEPVPATFKALVNGKEAFEELHDRIAN
AQHSIDIAIWGFQPSMHFKRDGKSPCIGDLLIQKALEGKKVRILVWSLPGSIQTFAEANLGNKPG
VWLNKVEGVTSEQVDYDRWWYEAIQGELDEVIVNAKTDGIVHVWEAHEIEKHEKLVEFTKSP
KRTNLIYKNRKVAPQNEDFKPRILPDGRKVNHSFEDTELPDGKGTLTDGSYDFALKKFKSHHQ
KTVLIDYEDPDLAVGFVLEHNMVDNYWDDSNHSLKTTLPNKGKNSPTPLQDVSSIVTGQVLW
DINHNFCQSWDRQNNKQWGKDPVDIGITGKRQSFTRDHYQPNPSLVDDSKLVMAQIVRTYDQ
PNIEDIMKVYLKNIKQTTSYIYTENQYFRFPPLVREFISHWETIKNNGRTEGPIHWFTVTNSSDE
GIGAGTYTTNEMFKLLGKQDVMPGVARNIKLNELETQLGMAKRSEVRLYNESMKAPTAEGKA
VAAAEFEKNQQEIQRIEKEIGEIKAKQHEEQKTQEAGKAGPTKKEGELNQIESSELGQEEPNLT
KELGYEISDTPGIKAHICTLMPKDENGKYVHTYKKNGKDTPAEVYVHSKVTIMDDVFTVISSA
NLNTRSMQVDTELGIIMECADVAEGLRKRLWDLHTNKNSAANPDDMHDYEVAEGAFKEWQR
LINENKKNMGDKSPKCALREFYRADPTVSRSD
>ZP_09805471
MSQNDIISPVATVDTSECAIITPWFVENTEYPPVPATYMPLVNGEEAFRSVHEAIANAKKTVDIIC
WGFQPSMYFIRDGKAPCIGELLVSIAKEKNVQVRILGWEAPFNSAGVAGESNVPGKDIIRFGDR
NGQSAEDKQHAYDRWWFSRFSNSGEWIKAINGPIVSPAQPTRNSPILTPVFVGRGFDLLERAEI
AYQELLYSVDSGLSGMSAAVMAIAPTHHQKTVLVDYELPDDAVGFVMGHNMLDEYWDKDA
HSSKRRPPALGREFPDRDTWAPNTGPRGKWPRQDMSCRVTGPLLEHLHQNFAQAWNRETEEN
LLLSRNAKCVAKKLKPLAGNGDLVMAQLLRTQMREGRRDIEKLYLKSVNNATQYIYIENQYFR
WPPLAEAIKENIKAQKKAGRIPEKDGYLYLFVVSNVTDEGIGAGTANTQRMLDSLGRGDTIPEL
TRLRRMEELKAKMYEPSPYPALSDEGIAEGFRKRAEYEKAAGELKKKVADDFMVPIDGLKVHI
CSLVAKDSPAEAWMPTYVHSKIMLINDVFTTHGSANINTRSMEVDSEMNIAHECAKVTKALRK
RLWNIHTGGQGVQDDPADAFDAWNKLIKRNHDNQYGEEKKTPETPLIQFLYDKTTLKDKD
>NP_900904
MSQPLAKHKPRQHNVDPSQHQTTRTSTPWFLADDHPVGYKPEFHEVNATFEPLINGERAFGAI
YDAILAAKHSVDIVCWGFQPSMYFKRGAGAPPIQYYLQQPQLPPLTADNIDKIADDMAGKLPF
TRRKLHPKDKIMPIGELLEMKSLEGVKVRILTWNDPISQIPEVMNPGYNVKRGDNENFLQECY
DIGWHQDIRANKNIQFRTRHFDDIDKNAYILPNLVRNGASTMHAAATSKFPSHHQKTVLIDYEH
PESALGFVMGHNMLDAYWDRDDHHYVQQEPHLGRNGATARHDISSRLTGPVLVHVNHNFVA
GWDKATGENLTGKRAHLELKHFTPRPNLGSRAMAQIARTQSQTRPKGKVKDELTVQDIKHLY
LNAVNHACQYIYIENQYFRWVNFAEATKQAVKNQLAFGRDPGQHGPVYLMVVTNDNAEGMG
LGVKNTYKMLDALGKRSQMPTLARSSINEQVKQAEREVQIGENQNAKRTSKPLSDLPERRKK
LAALKQEQAEANQGKFVDGDIPGLKTHVCSLVSMTGYQQGQPWQYVYIHAKLMMIDDTFMT
LGSANINLRSMVCDSEINVCHCQPDITRAARQHLWNMHTVGQGNTADASKPQPLADVYDRW
ERIMKANQKNRQSKRAPIAPLVKFSNDTSSTKDLD
>NP_231061

MDSFNYCVQCNPEENWLELEFRSENDEPIDGLLVTITNQSAPSNTYTQTTSSGKVLFGKIAAGE
WRASVSQASLLTEVEKYASRKEGQESPVKKRAAAELDAADKDTKQYRFTTIGDFWDEAPKDE
FLQKQHKGIDVNASAEKAGFRLSHNQTYVFEIKALRSYMPVIIDTDEFNLVNSYTFALLSKLAY
ATNDFNRDDGKTIDNQGAISTVISQLKRKERPTYSGDLQAKWLLEEIPYSKALSAQYYAEDDV
GSEGYIIFNDELAIIGVRGTEPYFQSKKPPVDNTKFKIIKAASGMAAVIADKIESATDSPGMKDLI
ITDLDAAQIAPEEFGGTYVHRGFYQYTMALLSLMEKDLGLHKIKKFYCCGHSLGGAGALLISA
LIKDSYHPPVLRLYTYGMPRVGTRSFVERYQNILHYRHVNNHDLVPQIPTVWMNTDVSEGFHV
LDVFKSRVDLMRKMLTDDDDDNYQHHGHLSQLLTYNSNNQVLLTPKQTQVTMLDLANLATN
DSVAMVDGLSDASIVEHGMEQYIPNLFEQLTALSDESLMVHYQRAISALEQEIATLQQSYLTVK
QAWIESIGNGTPTMNIGRLMSEMHSINKLIENRNKIRGELRQIVSDPQRMPATKFLISQQTLPDEI
KVQIR
>ZP_11083947
MTAATPRTLEGCVDCKMLKYEAEIKLVDELGQPLPNLPYVLWVGHGPKKIIRQGKQSSGDGVI
VEKELPPGPLYLMLEADALADVLQEPHRHLRLSRSDYGTPVQREAEQQGRLPRYARFGQLVDR
LPALFEEEQQKAKDKRHPLPPYHFPHGDPAKASAARRPLYIFTKAGARSIKITLEITPLRAWVLM
LEHSPEYNLGNAHNLALMAHLAYAGGDVNEANTKRVKEKRKTDPKYIPSASERAHSITHFFVE
QMQDLSRLPYGINALSKAALVKDVPYRERYETPIFIDCTELGEQGEAPPSDMIGGSYDTQFFYV
QRPEELIVSWRGTASLSDGLTDGNFTPVPCQGMNIADQGKAHEGFYNQFAAVNKHPKPSIAKV
YTDIANSLTGKKLFICGHSLGGALALLHAASLKAKNPLLYSYGMPRTFTESALRELTFPHFRHR
NERDKVTSLPPGRGVDTPLARMPVIGKVTSAAALLVPDKDPYAHHGKLVHFDYTDTSYRLRQ
ERGQDVMAPLPVQTKLLVVPHLAAEGKVNMPKEAKEALAKAYPSGDNPSHGGGAHASSHGS
GEYAGYLQRRFLGLLDGQQNLPDPYVPKLAEYVSTLKDYKAKIAPEVNWREHGAAQIDNQLQ
SLLPSGVTESEQFAITRLWQAPRK
>ZP_11985746
MTTAITSLKHKYPHAPAEEQLPYWVEILLVDEQGDAVSDMPWKVESHHPGDGIIKQFTYTGRS
GSDGLIRIDMPHGLELKLTLDADSLAKEMEKRPLRVGRDAEMDSLIRQLAEDKGYIWHYAVIG
ELCKVTPSIKLESGEGWPPYHFPHGKSFKGFIIRTNELERRHVIEICPFRAWELVLHHQKDYSIAN
GINLGVAATLAYADDNALSKSSITNFFINQCQDLSRLPRLYKDKSSWNTLVRDVPYSERYSPPVF
MDTSIDSSSKEDKDNQQGTISTKNDVSNVKADGDTQLYYVYNSDKIIIAWRGTESLFDAGTDIA
FNPVKTESCDVNKTQCAALVPAGKVHNGFWSGYLRLGRVFNNELDELLTLIKSRDLFVCGHSL
GGALALIHSAALKLEKPLLYTYGMPRTFTRNAIMELSDITHFRHINDNDPIPAVPMEANLDNEW
YKLWGLLGGTLGFFWSLGELMAYQLVAWGDCFWHHGNTVAFLTVTQSREWKEYKRNLPSPA
GGITIRKRLPVKAKLYLVPVLAEQEMQEAGQKQKEFQASLTKTDLTRFFPQGGNPERGVNINIF
EHFMTSYMPYMYNKLLELIDKAGIVEKRTFTEHLYNIDLFKMQMEENKGCIPDKEFSRNKIFLD
IENMLDVSLSSTLSMPSGNDTLLRFAKYSEEVMENA
>ZP_06124587
MPYQYNDPYCADCSKRKAWIECVLVDEFNQPLAEMAYTLKVRNGVIRKGVTDAQGYLRQED
LPRTVATLTIESQKLTDEMEKRPLRTLRGEAHSTVKPDALLQGYSYRYAVIGELCDKAPDIDKW
ESARFGLPYYHFPKENEFNGLKLMGEDFNQRHVIEVCPFRAWSLILNHTPEYDMVNAYNLGL
MSLLVYKNEVMVDPDKTEDMRDFINTPDTTTSFFYQQCFDLSQSPVIKDSHDYPAIVTDVPFN
QRYRPVIFLDVTQSENHQKGDHDTKLFFVENETQIIVAWRGTASLRSVLTDTTYQPIPCPTTLIPE
GKSNVHRGFLEAYQCVEKYFKENTNKIKDLSQDVDNKKLYICGHSLGGALALLHSSELRKNN
PLLYTYGMPRVFTISGAKSLSSLNHYRHVNDADSVTSVPFDTNMDSWLFEIGGGLGTTLGVLW
TVATLPTVPLQKSLPDFGEVYSHQGNPVSLFKARQIGEEIQYTHTGVTVGKKRWRMNGEYKFY

LVPNIANVLNDKLQKEQTGLVQSLGIDKQRLDDTFPQRNNPNLDTIITMPLDHFMGSQYQAIIN
NQLLFTVSPEKTPKHVASKQRFEALTHHQDAYKLNAERNTTFISLESKLTKGVTQQINQNSRLK
DALERYKEETHELPVS
>YP_002151060
MTFRYDKYNDPYCPDCAKHNAWIEILLVDELNNPISDMPYTLTVSGGEKRTGKTDRNGIVRET
DLPPTGGRFSINAQMLADEMEKRPLRVRRNGSSKIRYDATQRNDNYRYLTIGDLCDATPKILK
WDQPELPKYHFKKQQPNGYMVIFRNERWVFEVCPFRAWSFLLHHQKDYSITNACNLSILSVLS
YASFKANNDKEPNAGNYLGSIEDVFFNQLFDLSQIPQQFAKESFTPIIYDVPFSERYTDVEFIDSA
KNLDTQMFYIANNKEVIVVWRGTAGKTDIFTDIKFKPVKLRQDMGIEGYVHSGFYNSFRTMD
GKYKLRPKIGSKNDDENPLNLIKGLASNRKLFIAGHSLGGALALLHAIKLREYNPVLYTIGMPR
VLTLSITEQLGDIIHHRHVNEDDPVPALPFEKDMNNIAFISDHDWLGYSIEIAITYIDWKHQGMA
SKAIDQLKTNFNIKSDTFIHHGDAVHFYKPTSKTSMYKYVLASVGYAPSLLVNMEIDKKRYLVS
DTDEKLYLVSELLPDHRLKQEFFELYKLPKTASPGVTGGGDHSSRKYTNFIISKIISLVKYNGLTE
SAYDIRAELVNQKRLSQAKYDESSYLLDLDLMVADTLLPTLLMPEGITALKRFKYANDNN
>AAL18491
MSKLSPLDCLDCKNMLKHWIEFQLVDEQGKPLVNMPYRLKIRGNPLLGRKGVTDGNGLLREE
DMPPHPVTLYIGAQPLADEMEQRPLREIRGEGASVVKPKAEAEGYQYRYVTIGQISDGLPALD
DWNDPKKIPPPYHFPDPEPKGYQVHPVNRRYVLEVCPFRAWILLLHHQKEYSIVNAYNQCLMS
VLAYAGGDVDVEGSVLHFFNRQMVDVSKLPYKVEALSATPVVYDVPFSKRYTRVEFIDSQAG
NNKQGDTKLFYAASKKDMIISWRGTVSLDNYLTDATFQPLALSCADEKALCSEFIHHGKVHKG
FWEAFSLVGKLTVPSEETKVTTVFSDISDLVKNKLLFICGHSLGGALALLHSAQLKEHNPCLYS
YGMPRTLTRSAVEELSSIIHYRHVNEDDVIPAVPFEQDMDNVFFNYWAPAGYDWAVMKLLSPSP
IIQAIKQATASKEIYLHHGKVVHFFQANSCPEWLISARNASLITGVAERILDNTTKLYLIPELNQE
TEKDFSLAGERQNALFNQLSQQEKDKLFVENRSADLKGGFGFSNHSSYKYAGYIDKRLRELCE
PDKITVYQDSQRQFKAKMDSYKMLIPDNVYYRNLYFLDMDKQLIKSLTVSQQEEQGELALQH
YCDKQELSV
>YP_003467415
MSNANSIYCHDCSGMLKNKIEIQLVDEHNKPITNMPYTLKNHKMAREGITDGNGMIHEEHLTS
SPLRLFLDGQKLADEMEQRPLRLKRYHEYTRSTESMAKISAVFAESQKSGRQYRYAKIGELVD
KIPVIEGWKEEDPLPSYHFPDSEPMGIEVMPTAFERYRYVIEVCPFRAWSLVLHHQKDYSLVNA
YNLSLMSILTYSNDNSGHWGSVTHFFNKQLLDLSRSPYQVNDERFMPVVYDVPFSERYTKVV
YIDSKVQGNTGHTQLFYAANKQEIIVGWRGTEMTETQDLMTDGTFQPIELGSTANGVSSGFSE
KGKVHKGFWDAFHLITEIKVSEGNDKKTVFEEIIKLTESKKLFVCGHSLGGALALLHSAQLKSY
NPCLYTYGMPRLFTQSAVQELTEIIHYRHVNENDFVPSVPFNKDMDNVAFQAGRKFLGYAIEVF
DAAGSLEAKIIKSNNSDNDPFLHHGKLVYFYALPDSESKFYLLPELNEETIKTTQKFIKKQKNVD
RYIMQDIHSFFQGNENPTGKRGTGLFDHSSTLYAEYIDKRLRELCSLSPDKKLQRKPEDLSFLES
QPNSIRAIFQRAKTEEYFFLREIDNQLAITLEVTQKDERGPRALKRYFEKR
>YP_345882
MTSFEKDLQSPLGSRMLTCPAGGKWTSFQLIDEFCSGEPYAGLAYIVTDSEGHKYTGRLDEAG
KGKVNNHFAGPVTLLFDQAYEGKEKLYSYLQGRPHYPLKITELQVRAEATHYLNPNATRTRER
PEIADGGDYFQVEVRHLVRHASHLPPEVYRDYPLDSGCAAIMREHGKLGVALMPQRHTILEVR
PLRALRPILSTAPAFCALNLYQLALMATLSYCPFGQKPKVPCAETRSVKFLLQPSVGNWFGDAL
PKGEELWKVDSAQTKAYYPLYEDVPYSARLEIIPFDPNLYAVNRHTPERDPEHPASVHFLDDIGS
KDSTDTQAFITHNDELILIAVRGTAEIVADGLRDADALQVPFAEGEGQVHRGFYEAAKKAAAF

AVNYLEKFYTGQTLLICGHSLGGAITLLLAEMLRRRPEGYKIQLYTYGAPRAGDADFVKGAAD
LVHHRMVNHNDPVPSVPGSWMNTKADIYGTGAALTFVNVPLGLSVFVAGITNWTGEAYDHH
GRLRHAMPVEFGRQQVSSILWEPGCDTITQHAACDVAIRQRHGLPDRPTLLKQIFDAGHHSMT
GAYIPACWAVLRRWQEAQESNRTLVTEREFALVESALQRITDQLRRQRSNLPGRPDSYVRSRKN
IVEALNHEIENIRTTRERLASLRHRRLTTTDVYGSLAEQPERLAESLPRWRLHPENLATEQLAM
APEAAEDDPLLVTLYGHRIGAPHTFDIDSII
>YP_001349297
MRALSPLLSTGEAFCYLNLYQLALMSTLSYSPFGQEPDTQPVETDSVSFPAQPSVGNWFGDAL
ARSDELWQVDATQAGGKAYYPLYEEVAYSRRLEVVPFDPELYPEVNSPELGADQEHPARLHYL
DDAKKRGGTDTQAYVTHNDELMLLVVRGTASMADVLRDVDAAQTPFEETSGKVHNGFYESA
KVAIKFFATYLDKFYSGQKLVITGHSLGGAVALLVAEMLRQQPEKYDIVLYTYGSPRVGDKTFV
ENARPLVHHRMVNQNDPVPSVPAAWMKTSWRMSGAGVLLMLFNPAIGGAVVLLSPVNIVGEP
YTHHGKLRHFMPVSFADGHKSAILWTPGCESITERGGAAVCAKALAARNGLPERGGLLRQML
DNADHKMVASYIPHCWASLRRAQEALEEGRTVVTPTEYERVGEALQDFKAQLREKEAAAYGS
AREQQIMGPLRAEIGRLQQTAVRLKALSVERVSETKVYGRVADRPEALAVSLERWLAHSFNRS
HEQLAMAPPDADSNERAIAALVGGHVPGAVFDLDIDASG
>NP_793265
MTVQSIKTWEKPFFNNRIHACPMSGHSVSFQLVDEFGDGKPYAGLAYEVIDYEGVLYSGKLDA
NGSGKVDNHYCGPVVLKTCEEYVGEDDFYTALSGRGSYPLPITELQVRAEKTRFSNKSGVRTR
SNPAQSEADAFCQVEVSELVKHACHLPPIVDRNFPPNEYVRNLMQTPPEDVGAGESGFGPKPA
RVYGIGLLPNKHHVLEVRPLRALRPALSTDSEFCALNLYQLALMATLSYSDFGQKPDLFKVKS
DSDEAYVVEADTVSFPLQPSVGNWFGNTLSKFEELWQVDAAQAGGKNYYPLYEEVAYSKRLE
IVPFDPDLYPEVNRPSLGDDQEHPAKIHFFDDTTSKNGTDTQAFITHHDEIVLLSVRGTASTSDA
FRDLDAAQVPFEEGVGKVHNGFYGSAKAVINFVTSYLDRFHVGQKVIVTGHSLGGAVAFLVAE
MLRRRKGYDYDIVLYTYGAPRAVDETFATAATALIHHRTVNHTDPVPSVPTTWMNTSKPVYIT
GAIVTFVNVPIGLALFGGGIANLTGEPYTHHGKLRHFMPVSFADGHKSSIIWEPGCDTITEHAAC
TVALQQKSGLPKRGGTLRQIIDNANHKMVVSYIPACWAVLRRYQESQEFKRSLVTEREFRWVD
DALESISLQLQAKERQFTMQALESQVEAQKQALRDERSKIQETRDRLGTLRFTKATETQVYGL
AAAVPEALAVNLERWRLHAINTTLEQLAMAPPDADSHDRAVASITGGHIIGAPSHFDVDSFA
>YP_610877
MGHNQFLKEVNHPLNNIMLVCPVRGVSTSFQLVDEQGHGAPYAGAFFEVVDMDGTTYKGAL
DTEGRGEVLNHCRGPVSLRFASEYAGGDDGYMKLQTRDFYPLKITDIQVRAEQTHFQNRDGR
RTQSNPAAVSADEFYQVEVSELVCHICHLPPRSLSDFPSDLGIRRIMGKHCEWGVGLMFGKHTV
LEVRPLRALRPVLSMDQEFCALNLYQLALMATLSYTPFGQEPPGHPVKAKSVNFPYVPTVGN
WFGDALAKGQEIWRVDTKQQTEYFPFYEDVPYSQRWEIVPFDPELYEENDPALGEDQKNPARI
HFLDDREYSDTTDTQAFMTHNADVMIIAIRGTSEKIPDLLRDVDALQVPFEEGHGKVHRGFYL
AAKRALQFVEVYMDKFYQSQQLIICGHSLGGAVALLLAQMLRTGGYSGPLQLYTYGAPRVGD
STFLASAADLRHHRIVNNDDMVPNLPLPWMNTRYEVIATGAVLASINFPLGAMVMRTGLVNQ
DGEPYGHHGELQHFMPIQLSNQESSAILWRPGCTTITAQPACNYYLEKVDGLPQHRSVSLADH
FMVSSYIPACWAMLRRHQQALANRTPAVTVRELEAVDTALASISQQLRERRSRLSRGDYYPRSR
EPSLTAIEKELERLQATRERLASLRRAPVSEADVYGSLAGQPQLAEALDRWQAHAASTQAAPL
AMAPKEQDINVVTFDELFASLDDPLDLI
>YP_001186316
MSDSPHTDMTETLTCPLRGHWVSVRLVDEHGNGKIYAGLNYVLYDSQGQKYQGSLDEDGFSR

IDGIYCGPTVLSFADEYLGQDSWYVGLIRREKFALPLVALQVAAEQSPCGPRKPDGKTYLAEER
ALSENARFLRVEVRDLVEATAHLPDRDTAWWPRPSAVLKRNAGLAAERTGVALTPNLHHVLEV
KALRAYSPILSRDKAFCALNAYHLAVMSTFVYAPFGEPKREDADYTSSPPPYPRAGSIGHVLRE
QLASLNKANAFNSARYHLLYEEVAYSKRLEIMPYDPERYQAEAAGEWSNPEDVHFLYDTGTDT
DTDTDTQAFITHSDKVVLISVRGTQETPDILRDMDARQVPYEEGIGQAHRGFYRAFKAAKVFT
QRYMDAFYTGEQTVIVCGHSLGGAIALLLAEWLRRLPTAPDVVLYTYGAPRAGDRAFVQGAQ
ALVHHRLVNHNDPVPAVPFTWMDAEWKLATAGTVTLFSSPVLGIVLLLGGLLNLKGDPYEHH
GEQRHFVPRKANGGSEASILWQPNCALIDEQACARYAGAIDLQGDMPKRMSFIEQAFSAAEHS
SDGGYSRAMLTTLLRWNASVEERDGALFNAGEIRDIHNLIEQAEAQLASWRPRSFMEFRRAVR
AHHDTRFYNKSDLELRRMYVEGITLARSLSRQQKEALARAKQRLLNESERRLTVRDVFGDQT
EREDLSELVAQWRAIGENQKAEKLAGIALSNPHYA
