# Supplementary material for: A phospholipase effector of the type VI secretion system modulates plant reproduction
Source: mBio. 2025 Aug 5;16(9):e01546-25. doi: 10.1128/mbio.01546-25 (PMC12421835; doi:10.1128/mbio.01546-25)
Supplement: Data S2 — TleB homologs sequence for Fig. 2K. [file mbio.01546-25-s0003.pdf]

Supplementary Data 2. TleB homologs sequence for Figure 2K.

>PXO_02032 *Xanthomonas oryzae* pv. *oryzae*
MMSAGLGRPQDSCCIDLRWGFFFDGTNNNFQRDQPKKAHSNVARLYDIFEAD
RRKPEFVRRYAAGVGTAFKDEVGDQGLGIQEKAGLAAGWGGEARICWALLK
FLDNLNYYFERIDLGEALGQKDPGTVRRMAQDMTIPSMELRKIAGDETEMLR
QISMMASLQSLTATALNPPNHRGRRAVLAERRAQLRQRVQTWQRAQPKPKLR
SIRVSVFGFSRGAAEARVFCSWLKDACDGGGGELTLCGIPVQLDLLGIFDTVAS
VGLANSSRLWSGHGGYASEDDLRIAPDVRRCVHREGHD

>PXO_02034 *Xanthomonas oryzae* pv. *oryzae*
MQVSFAPLCPENPLQLSRAQQAEMMSAGLGRPQDSCCIDLRWGFFFDGTNNN
FHRDQPKKAHSNVARLYDIFEADRRKPEFVGRYAAGVGTAFKDEVGDQGLGI
QEKAGLAAGWGGEARICWALLKFLDNLNYYFERIDLGEALGQKDPATVRRM
AQDMTIPSMELRKIAGDETEMLRQISMMASLQSLTATALNLPNHRGRRAVLAE
RRAQLRQRVQTWQRAQPKPKLRSIRVSVFGFSRGAAEARVFCSWLKDACDG
GGGELTLCGIPVQLDLLGIFDTVASVGLANSSRLWSGHGGYASEDDLRIAPYV
RRCVHLVAAHEVRGSFPLDAAAGVNGEEVVYPGVHSDVGGGYEPGEQGKAF
IGDSIDDSAKLSQIALCHMYREAMAAGVPLNLSASRLSKETKAAFKVDKGLID
AFNGYVAATGSIKASTTVALTQAHYALYLRWRRLRLDDTAPDGMAQQPFVTR
ARTYKAQDVTDLLQTNAELRQEWAALQQDEKDAAYSSEASVAHVLRSTLAPI
AARDDIVALVWGEKMTQWREVKPAWNDLSPLDRRIVRLHDDYSHDSRAWFK
PFGAASEEAWKRQYRQRMNRLEAQDNAWQQWNRDVQPVIDDAVRKAQKH
PGSFQPTPEVRPMPPLVAGQDLKDLKQWRSNGGVIPTEQDGRESYGMFGFLR
WRTIFVPEKSALAHSIDAVDETLEQIKQLPGKAKQAVGDAVESAVDSAVEAGK
DFVGDQVRKLIPSGLPRM

>WP_237654774.1 *Xanthomonas translucens*
MQVSFAPLCPENPLQLSRAQQAEMMSAGLGRPQDSCCIDLRWGFFFDGTNNN
FQRDQPKKAHSNVARLYDIFEADRRKPEFVGRYAAGVGTPFKDEVGDQGLGI
QEKAGLAAGWGGEARICWALLKFLDNLNYYFERIDLGEALGQKDPGTVRRM
AQDITIPSMELRKIAGDETEMLRQISMMASIQSLTATALNPPNHRGRRAVLAER
RAQLRQRVQKWQRAQPKPKLRSIRVSVFGFSRGAAEARVFCSWLKDACDGG
GGELTLCGIPVQLDLLGIFDTVASVGLANSSRLWTGHGGYASEDDLQIAPYVR
RCVHLVAAHEVRGSFPLDAAAGVNGEEVVYPGVHSDVGGGYEPGEQGKAFI
GDSIDDRAKLSQIALCHMYREAMAAGVPLNLSASRLAQRSKDAFKVDKSLID
AFNGYVAATGSIKASTTVALMQAHYALYLRWRRLRLDDTAPDGMAQQPFVT
RARTYKAQDVTDLLQANAELRQEWAALQQDEKDAAYSSEASVANVLRSTLA
PIAARDDIVALVWGEKMKQWREVKPAWNDLSPLDRRIVRLHDDYSHDSRAW
FKPFGAASEEAWKREYRQRMNRLEAQDNAWQQWNRDVQPVIDDAVRKAQK
NPGSFQPTPEVRPMPPLVAGQDLKDLKEWRSNGGVIPTEQDGRESYGMFGFL
RWRTIFVPEKSALQHSIDAVDEKLEQIKQLPGKAKQAVGDAVESAVDSAVEAG
KDFVGDQVRKLIPSGLPRM

>WP_229011180.1 *Xanthomonas hortorum*
MSAGLGLPQDSCCIDLRWGFFFDGTNNNFQRDQPKKAHSNVARLYDIFEADR
RKSEFVRRYAAGVGTPFKDEVGDQGLGIQEKAGLAAGWGGEARICWMLLKF
LDNLNYYFERIDLGEALGQKDPATVRRMAQDITIPSMELRKIAGDETEMLRQI

SMMASIQSLTATALNPPNHRGRRAVLAERRAQLRQRVQKWQRAQPKPKLRSI
RVSVFGFSRGAAEARVFCSWLKDACDGGGGELTLCGIPVQLDLLGIFDTVASV
GLANSSRLWTGHGGYASEDDLQIAPYVRRCVHLVAAHEVRGSFPLDAAAGV
NGEEVVYPGVHSDVGGGYEPGEQGKAFIGDSIDDSAKLSQIALCHMYREAM
AAGVPLNLSASRLAQRSKDAFKVDKSLIDAFNGYVAATGSIKASTTVALMQA
HYALYLRWRRLRLDDTAPDGMAQQPFVTRARTYKAQDVSDLLQANAELRQE
WAALQQDEKDAAYSSGASVANVLRSTLAPIAARDDIVALVWGEKMKQWREV
KPAWNDLSPLDRRIVRLHDDYSHDSRAWFKPFGAASEEAWKREYRQRMNRL
EAQDNAWQQWNRDVQPVIDDAVRKAQKNPGSFQPTPEVRPMPPLVAGQDLK
DLKEWRSNGGVIPTEQDGRESYGMFGFLRWRTIFVPEKSALQHSIDAVDEKLE
QIKQLPGKAKQAVGDAVESAVDSAVEAGKDFVGDQVRKLIPSGLPRM
>WP_234416390.1 *Xanthomonas fragariae*
MQLSRAQQAEMLSAGLDRPQDSCCIDLRWGFFFDGTNNNFQRDQPKKAHSN
VARLYDIFEADFEKPEFVGRYAAGVGTPFKDEVGDQGLGIQEKAGLAAGWGG
EARICWALLKFLDNLHSYFEKIDLGKALGQSDPATVRRMAQDITIPSMELRKIA
GDETQMLRQISMMASLQSLTATALNPPNHRGRRAVLAERRAQLRQRVQKWQ
RAQPKPQVRSIRVSVFGFSRGAAEARVFCSWLKDACDGGGGELTLCGIPVQLD
LLGIFDTVASVGLANSSRLWTGHGGYASEDDLQIAPYVRRCVHLVAAHEVRG
SFPLDAAAGVNGEEVVYPGVHSDVGGGYEPGEQGKAFIGDSIDDSAKLSQIA
LCHMYREAMAAGVPLTLSASRLSKETKAAFKVDKGLIDAFNGYVAATGSIKA
STTVALMRAHYALYLRWRRLRLDDTAPDGMAQQPFVKRARTYKAQDVTDLL
QANAELRQEWAALQQDEKDAAYSSEASVANVLRSTLAPIAARDDIVALVWGE
KMKQWREVKPAWNDLSPLDPRIVRLHDDYSHDSRAWFKPFGAASEEAWKRQ
YRERMNKLDAQDKAWQQWNREAQPVIDDAVRKAQKNPGSFQPTPEVRPMP
PLVAGQDLKDLKEWRSNGGVIPTEQDGRESYGMFGFLRWRTIFVPEKSALQLS
IDAVDEKLEQIKQLPGKAKQAVGDAVESAVDSAVEAGKDFVGDQVKKLIPSGL
PRM
>WP_065470271.1 *Xanthomonas bromi*
MQVSFAPLCPQNPLQLSRAQQAEMMSAGLGRPQDSCCIDLRWGFFFDGTNN
NFQRDQPKKAHSNVARLYDIFEADRRKPEFVRRYAAGVGTPFKDEVGDQGLG
IQEKAGLAAGWGGEARICWMLLKFLDNLNYYFERIDLGEALGQSDPATVRR
MAQDITIPSMELRKIAGDETQMLQQISMMASLQSLTATALNPPNHAGRRAVLA
ERRAQLRQRVQKWQRAQPKPQVRSIRVSVFGFSRGAAEARVFCSWLKDACD
GGDGELTLCGIPVQLDLLGIFDTVASVGLANSSRLWTGHGGYASEDDLQIAPY
VRRCVHLVAAHEVRGSFPLDAAAGVNGEEVVYPGVHSDVGGGYEPGEQGK
AFIGDSIDDSAKLSQIALCHMYREAMAAGVPLNLSASRLSKETKAAFKVDQG
LIDAFNGYVAATASIKASSTAALMQAHYALYLRWRRLRLDDTAPDGMAQQPF
VKRARTYKAQDVTDLLQANAELRQEWAALQEDEKDAAYSSQASVANVLRST
LAPIAARDDIVALVWGEKMKQWREVKPAWNDLSPLDRRIVRLHDDYSHDSR
AWFKPFGAASEEAWKRQSRERMHTLDAQDKAWQQWNREAQPVIGDAVRKA
QKHPGSFQPTPEVRPMPPLVAGQDLKDLKEWRSNGGVIPTEQDGRESYGMFG
FLRWRTIFVPEKSALQQSIDAVDEKLEQIKQLPGKAKHAVGDAVESAVDSAVE
AGKEFVGDQVKKLIPSGLPRM
>WP_152249237.1 *Xanthomonas maliensis*

MKISFAPPCPANPLQLSRAQQAELLASGLDRPQDSCCIDLRWGFFFDGTNNNF
QRDQPKKAHSNVARLYDIFEADFEKPQFVGRYAAGVGTPFKDEVGDQGLGIQ
QKAGLAAGWGGEARICWSLLKFIDNLHYYFERIDLGQALGQNDPATVRRMA
RDINIPSMELRKIAGDETEMLRQISMAASIQSLTATALNPPDHSGRRKVLAERR
AELRKRVEQWQRAQPKPRVRSIRVSVFGFSRGAAEARVFTSWLKDACDGGN
GDLRLCGIPVQVDLLGIFDTVASVGLANSSRLWKGHGGYADEHDLRIAPYVR
RCVHLVAAHEVRGSFPLDTAAGVSGEEVVYPGVHSDVGGGYEPGEQGKAFIG
DSIDDSAKLSQIALCHMYREALAAGVPLNLSASRLAQRSKDAFKVDKGLIDA
FNGYVAATAGIKAGNSIALIQAHYALYLRWRRLRLDDSTPEGMAQQPFVKRA
AKYKQQDVTDLLKANAELRQEWAALQQDEQDSAYSTQSSVGRVLRRMTILS
VRDDFVAALWGEKMKQWKEVKPAWNDLSPMDPRIVRLHDDYSHDSRAWFK
PFGAANEEAWKRQYRERMAKLDAQDKAWQAWNREVQPVIGDAVRKAQQN
PGSFQPTPEVRPMPPLVAGKDLEDLKAWRSSGGVVPDEAEGRESYGMFGFLR
WRTIFLPAPSAEASAFQKSMDAVDEKLDQIKQLPDRARRAVGDAVNSTVDSAI
DGVIDSGKEFVGDQIKKLIPTGLPRF
>WP_231732391.1 *Stenotrophomonas*
MSDPEQDCYVDLKWGFFFDGTNNNLGRDRPHLAQSNIARLYDVFDLGRAGS
ENIKQYIAGVGTPFEEEVGDQGKGVHQAAGLSAGWGGEARINWALLKVLDN
LHQYYEGVGLGRMLGVLDPQAVKGMSADMNIPIHQLRKLTGDEVELLREIG
MPKSVRLITSTAATSPNDPARRATLKARREVLASKLSAWRRTRKPRVRVIRISV
FGFSRGAAEARVFSSWLKDALDPDFTLCGIPVQVDFLGIFDTVASVGLANSSIA
FDGHGGWGREQDMEVPDYVKECVHMVAALEVRASFPLDAYRGLAKRATQL
VYPGVHSDLGGGYIPSEQGKGYVGAEGRDAAKFSQIPLQEMYQRARAAGVP
LNKDNGMLAESAREAMEVDQALISAYSDYFEATGGVSYGSLQQIMHDHYGR
YLQWRKLRLGDDAPAGLKHQPFVSRAGKKSGQDVVDLVMANYELKWEYEA
LLKDERSDPALRIPNGLSDNISKFIGLRARNIFPLASLRDAAVSYVWGYKMKC
WQEIKPIWEDTSDVDPRISRLMDDYIHDSRAWFKPFGAPNEAAWKRQQEERM
KLLEAQDARYKEWERIKQEDERILREGNALERMAAAHRRSAMNSTPQAWPK
RLEGADRKDLDAWKSNRQLPYEIEGRESWSIFGYLRWRMVYEDEASWLQQV
GDFAGAQARAVKRGIKDKVDDAVDRAAEAAGRAIGNGAKKGADYLLDKAK
DALSNGVPRTRL
>WP_123087829.1 *Lysobacter psychrotolerans*
MRIRSAPECSSQPTNLSIQGRSDALCNALVGATAQCEIDLHWGFFFDGTNNNL
KRDAPDFTHSNVARLHEVFSETRDETRLRRYIAGVGTEFNKQIGDRGQGAQQ
KAGLAAGWGGEARLCWALLKFLDNLSRFLCQRELGEILGQPDPTTVRTMATD
IGLPRYQLEQMATDEAQAIRMLRSVGMAASVAATYDTANADPNHAGRRRVL
AERRMLLEKQMEQRHAARIKPEIGRIRLSVFGFSRGATEARVFCNWLREACDP
APGGGLNLCGIPIEFDFLGIFDTVASVGVAKITTVFDGHGNYAQEADLRIPDYV
KRCLHLVAAHEVRASFPLDTAWDSNCEEVVYPGVHSDVGGGYRPGEQGKGY
VDGKASDAAKLSQIPLAHMYREAVKAGVPLDLDAATDKATAAFNIAPQLIAA
FNGYVRASQSVRGASTRELVQGHYGLFLRWRRLRMQDGAELFANQPFVARA
KAFKQQDYEDLVRANRELISEWQFLVTLERVGLAVAAQNVLVQTILKLHAYRT
AATWVLQQRLRRWLEVKPYWNETQPLDSRIVRLFDDYVHDSRAWFKVLGA
ASEAAWIQEQGERMVELERRDAAWQSWKQQALRSPGFAARSPPPPSISAEDK

QALQAYRRSGGRDLPEERSGREPYELFGFLRWRTSFPGGESFRARERLQDEAR
RAHEAAVQAHEAAAKAAIRNIRFGPNGVPLIL
>WP_052511451.1 *Cupriavidus* sp. HPC(L)
MHMEKIDIAHPFALTKDERARMLAHQVARKVSSCCEEIDIGIFFDGTNNNKDR
DAPRNAHSNVGRLFDAFDVPTRDLTRYKYYCAGVGTPFFKETGDTGRGYDS
RAGLGAGWGGEARINWALLQISNAIHFRFFRVDLSGALQTTDRACVEKMSTD
VNMADRLLRSAGDTEIEQLRNAGTLGTTGYILDTAAMAPNHAGRKSVLKQR
RDYLRSKLAPVLASHKPALKRIRLSVFGFSRGAAAARVFCNWLTDALDKDMT
IAGIPVELNFLGIFDTVASVGFAQSFLLFEGHGGWAQEQYLRIPSYVKRTVHLV
SAHEVRGSFPLDKAEAENCLELVYPGVHSDLGGGYVPGEQGKGCDEKGAPE
DSCKLSQIPLARMYREAVAAGVPLDINASSMAPEAKQAFKVSSSLARAYNDY
VDVANKVVSLQGGGTTGAAKAQYGMYLRWRKLRLPGTPEALENQPFFKRAE
KFSRQDAEDLRSANRELQAEAAALPAMEKASGLIDGWMRRSGLAWTPVAAG
PVLMGQLYQQVAGEKVKQWREVKSIWNHKEPLDSRVVRFFDDHVHDSRAW
FKPLGATSEEVWKLQQKERMERLKKQHEDFQQLMKDLSRDLHGTVKRYTPT
HEGGGGEVAPLPLTGEQLAQLEAYKKGQLPTETEGREWSSIWGYLRWRTHYQ
PERTLGEKVMAIWGEATSLPERAVDKAKDAVTDLESAMKKKAAEFLGTGIEK
GTDYLQRQAADALRRFLGNGIPTF
>WP_096745538.1 *Ralstonia pickettii*
MAELERLDSNPFSLTLDEKARCMANHMETFSGQCTEEFRIGFFFDGTNNNKD
RDAPKQAHSNVARLFDLFEEKDEQVKVYIAGIGTPFQKEIGDTGRGYDARAG
LMAGWGGEARINWALLQVTDALYRRYYGDNVSRAMGSTDLATVRKISADLS
FPVHKRAAAARDEAQKIKDEAEELNDTSTVRTLGGAADTADNFPNDIARKKE
LKRRREFVSQKLKQLIADRKPKLLRIRLSVFGFSRGAAEARVFSNWLKDALDD
DMTLAGVPVSFDFLGIFDTVASVGFAQSSMFSTGHGGWGQEEFLRIPKYVKRT
VHLVSAHEIRGSFPLDKAVANNCLELAYPGVHTDVGGAYQPGDQGRGCRADG
TPDDSTKVSQVTLAKMYREAVAAGVPLNPAAANLSQKAREALKISPQLIKDY
NAYVDAVNSLIRKRGGGTTGAAHVQYGLYLRWRRLRLGSGADAFEKQPFFK
RAQNFSAQCAQDLSGANEELRDEAKTMAEMENSIAYSDGWMTAVLRNTGLP
AAAVLKIQRSIWGEKVAQWRQVKQYWNDTSPLDPRVVKFFDDYVHDSRAWF
KPMGATNEGVWKLQQKARLEELKRKDAQWKANWGAEWQKLNTDIQRDPR
GTLERYAPLSEGGKGEVAPPPPPVMGQDRIDLDRYLKDGSLPLEKTGREFSSV
WGYLRWRTHYAPEPTLGERAQAAWNKVTAATDKVVQKGKNAAHDAEESLA
RTARKLLEAGQDSLEHAAKDVLQRFMDGGVRPL
>WP_064046074.1 *Ralstonia solanacearum*
MAELEHLGPDPFALTLDEKASCMASYMEAVMGQCTREFRLGFFFDGTNNNK
YRDAPRQAHSNVARLFDIFDISPDQQALYVPGIGTPFEKEIGDTGRGDHARAG
LGAGWGGEARINWALLQVTDALYKLYYGRNLSIAMGADDLSLVRQISSDLNL
GLANVRTAGRDEVEQLANATNWEMLTSAAGTVLKPPRHEERRAVLRERREFL
SQKLKALIAGRKPKMLRIRLSVFGFSRGAAEARVFANWLKDALDDDMTLAG
VPVSFDFLGIFDTVASVGVANSTKVATGHSGWGEEEFLRIPSYVKRTVHLVSAH
EVRGSFPLDKAVADNCLELAYPGVHTDVGGAYQPGDQGRGCNADGQPDDSN
KLSQITLAKMYREAVAAGVPLNPSGRNMTREMKNALKISPDLIKAYNDYVDA
VNPLIRKRGGGTTGAAQVQYGLYLRWRRLRLTGGAQAFESQPFFKRAQHYG

AQCANDLSVANALLREEAKDLETRENDPAYSDGWMRRVLVVLPITQGTAMA
DRLKQTVWGDKVREWREVKAYWNDTSPLDPRIVRICDDYIHDSRAWFKPFG
APSDSVWRMRQQARLEQLKQQDAAWKQVAADLNRDLIGTLKRYESTANGG
EGEVPPNPVVGQDRIDLDRYLKEGSVPMETKGREPSSMWGYLRWRTHFAPTP
TLGERAQAAWDEVAAVPGKVARKAKDVAHDAEARLANTARKLLDAGQESLE
RAAKDALQHFLGSGGVPRL
>WP_104611227.1 *Ralstonia mannitolilytica*
MAELEQLDPNPFSLSVDEKGRCMASNMDASSGQCTQEFRIGFFFDGTNNNKD
RDSPKRAHSNVARLFDIFEVTPEQVRFYVPGIGTPFQREIGDTGRGYDARAGL
GSGWGGEARINWALLKITDALYRYYYIRDLSADMGTDDLKLIKKISTDVNLP
VHLRASAGSDEVDELNNAGTFGTLAGTLDTATLAPNHQARRHELARRREFVE
QKLKPLIDGRKPTLKKIRLYVFGFSRGAAEARVFCSWLQDALGKDMTLAGVK
VSVDFLGIFDTVASVGFAQSFMIASGHGGWGNEEFLRIPGYVERTVHLISSHEI
RGSFPLDKAESKNCLELAYPGVHTDVGGAYQPGDQGRGCRADGTPDDSTKM
SQVTLAKMYREAVAAGVPLNPAAANLAQKFKDALTISPQLIKDYNDYVDAVN
PLIRKHGGGTTGAARVQYGLYLRWRRLRLNAGAQAFENQPFFKRAQKFSAQ
CAQDLAGANEELREEAKIMAEMESSPVYSDGWMGKVFRNVPGPGGVVAEIQ
QFVWGEKVKQWREVKSYWNDTSPLDARIVKFFDDYVHDSRAWFKPTGATN
EEVWKRRQRERLEDLKRKDAEWKAQWAEINAASARDPEGVRKRFAPVDEG
GQGDIYPPLLVGQDRTDLDRYLKDGSLPLEKTGREFSSVWGYLRWRTHFAPTL
TMSERAQAAWNKASKIPGKVADKITDKAKDVAHSAEEKAAEAAGKILEAGK
ESLIRGAKDTLDRFTGGGIPRL
>SFY31574.1*Pseudomonas* sp. NFPP02
MQLEKLGENPSALLQDEQAQFLSNQDISNNMECCTSLHLGFFFDGTNNNKER
DTPKQAHSNVARLFDVFEETSNTVKIYVPGVGTPFPKETGDTGRGYHARAGL
AAGWGGEARINWALLQLSNQLYRYFEKTTLSDASNIQDVDLVKKISTDINLPQ
TALKGAGTTEVEQLNNAGTLGTIGNIIDTAYIEPRHRQRRELLQARHDELTQKL
ASRIASDKPKLVSIRLSVFGFSRGAAEARVFCNWLSDALDKNRTLAGVPVNID
FLGIFDTVASVGLAQSALLFEGHGGWGQERFLRVPNEIKKVVHLVSGHEVRGS
FPLDIVGDHPNCIELVYPGVHSDIGGGYPPGDQGRGCSTNDAPDDGSKLSQVP
LAKMYRSAVAAGVPLNASNPRLAPEIKNAFTISPALRQSFNDYVAIVNAMTPS
NASTTQAANIQYGLYLRWRRLRAANNSNALEQQPFFSRAKINSPQDAEDLRR
ANDELRREVVVLSERENDLVYSDGWMARTLLQSMPASALSSEIERAIWGEKV
RQWREVKPFWEYEKALKPEIIRLFDDYVHDSRAWFKPFGATNEDVWKHNQTI
RMKKLMQQEATYRQYQTEFERDAKAAIKRYAPYDEGGYGDVAPSPVWGLDR
IDLDRYLKDGSLPVEPDGRETSSIWGYLRWRTHYSPEDTLVEKAQRAWNKAK
NATNEVIDGARKRVTNAAADTAQEVADTVMEEAGKTVKRIINRQPWR
>MBA6041754.1 *Pseudomonas lactis*
MQSKILRLSLLRDLNMQLEKLGENPSALLQDEQAQFLSNQDISNNMECCTSL
HLGFFFDGTNNNKERDTPKQAHSNVARLFDVFEETSNTVKIYVPGVGTPFPKE
TGDTGRGYHARAGLAAGWGGEARINWALLQLSNQLYRYFEKTTLSDASNIQ
DVDLVKKISTDINLPQTALKGAGTTEVEQLNNAGTLGTIGNIIDTAYIEPRHRQ
RRELLQARHDELTQKLASRIASDKPKLVSIRLSVFGFSRGAAEARVFCNWLSD
ALDKNRTLAGVPVNIDFLGIFDTVASVGLAQSALLFEGHGGWGQERFLRVPN

EIKKVVHLVSGHEVRGSFPLDIVGDHPNCIELVYPGVHSDIGGGYPPGDQGRG
CSTNDAPDDGSKLSQVPLAKMYRSAVAAGVPLNASNPRLAPEIKNAFTISPAL
RQSFNDYVAIVNAMTPSNASTTQAANIQYGLYLRWRRLRAANNSNALEQQPF
FSRAKINSPQDAEDLRRANDELRREVVVLSERENDLVYSDGWMARTLLQSMP
ASALSSEIERAIWGEKVRQWREVKPFWEYEKALKPEIIRLFDDYVYDSRAWF
KPFGATNEDVWKHNQTIRMKKLMQQEATYRQYQTEFERDAKAAIKRYAPYD
EGGYGDVAPSPVWGLDRIDLDRYLKDGSLPVEPDGRETSSIWGYLRWRTHYS
PEDTLVEKAQRAWNKAKNATNEVIDGARKRVTNAAADTAQEVADTVMEEAG
KTVKRIINRQPWR
>WP_048403003.1 *Pseudomonas weihenstephanensis*
MQLEKLNNDPSALTLAEQAQFLSNQNLSSNMDCCTNLHLGFFFDGTNNNKD
RDTPKLAHSNVARLFDVFEVTKNTVKIYVPGVGTPLPKETGDTGRGYHARAG
LGAGWGGEARINWALLQLSNQVHYFFTNKTLSEVNGLEDIELVKRISTDINLP
QADLIAAGNTDVEQLKNASTIPTIGKILDTAYFEPRHTERRKLLQSRHNDLTQK
LSSQIASVKPKLVSIRLSVFGFSRGAAQARVFCNWLTDALDKNRTLAGVPVSID
FLGIFDTVASVGVAQSTLLFEGHGGWGQERFLRVPKEIKRVVHLVSGHEVRGS
FPLDIVGDHPNCIELVYPGVHSDIGGGYPPGDQGRGCSTNDMPDDGSKLSQVP
LAKMYRSAVAAGVPLNINNPRLAPRIKNAFTISPALRQSFNDYVAIVTAMTPSN
ASTTQAATIQYGLYLRWRRLRITNNSNALEQQPFFARARINNRQDAEDLQRAN
DELRREAVVLRERENELVYSDGWMARTLLQSVPASALSSEIERAIWGEKVRQ
WREVKPYWEHEKALKPEIIRLFDDYVHDSRAWFKPLGAPSEDVWKHNQTIR
MKKLMQQEAAYRQYQAEFQRDTKAAIKRYAPYEEGGSGDVAPSPIWGLDRID
LDRYLKDGSLPVEPDGRETSSIWGYLRWRTYYSPQDTLTEKAQRTWNTAKKA
TNEAIDGTRKRVVDAAADTAQEVADTVIEEAGKTVRRIINRQPWR
>WP_096746043.1 *Ralstonia pickettii*
MTVLERLEPNPFALTPDEKGNCTANNINAFSGQCTQEFRIAYFFDGTNNNKDR
DAPLKAHSNVARLFDIFEVTPDQVRFYVPGIGTQFQKEIGDTGRGYDARAGLG
SGWGGEARINWALLKLTDALYGRYLGDDLSKAMGTDDLKLIKKISTDVNLPV
HLRASAGSDEVDELNNAGTFGTLAGTLDTATLAPNHQARRQELARRREFVEQ
KLKPLIDGRKPTLKKIRLYVFGFSRGAAEARVFCSWLQDALGKDMTLAGVKV
SVDFLGIFDTVASVGFAQSFMIASGHGGWGNEEFLRIPGYVERTVHLISSHEIR
GSFPLDKAESKNCLELAYPGVHTDVGGAYQPGDQGRGCRADGTPDDSTKMS
QVTLAKMYREAVAAGVPLNPAAANLAQKFKDALTISPQLIKDYNDYVDAVNP
LIRKHGGGTTGAARVQYGLYLRWRRLRLNAGAQAFENQPFFKRAQKFSAQC
AQDLAGANEELREEAKIMAEMESSPVYSDGWMGKVFRNVPGPGGVVAEIQQ
FVWGEKVKQWREVKSYWNDTSPLDARIVKFFDDYVHDSRAWFKPTGATNEE
VWKRRQRERLEDLKRKDAEWKAQWAEINAASARDPEGVRKRFAPVDEGGE
GDIYPPLLVGQDRTDLDRYLKDGSLPLEKTGREFSSVWGYLRWRTHFAPTLTV
RERAQAAWTKASQIPGKVADKITDKAKDVAHSAEEKATEAAGKILEAGKESLI
RGAKDTLDRFTGGGIPRL
>WP_095747218.1 *Variovorax boronicumulans*
MTTCKLLEPIGEDPLALTKAQKALCLASNMSTAGLPCSKELHFGFFFDGTNNN
RIRDTPQRSQSNVARLYDVFDATPDENARIYVAGVGTPFMAEVGDSGLGAAA
NAGLGAGWGGEARINWALLQLHDKLFRYFHRGSLSFSESLGTNDQATVKEM

SADINMSPGAIRNLGKTEVQMLREVGGLTKMGNIVETAMTSVRDEARSDILRT
RRQALWTKLSNMVQQRNPKLEKLCISVFGFSRGAAEARAFCNWLTAALDPDF
TLAGIKVEVVFLGIFDTVASVGMAQSFLVKDGHDAWGRPKDLAIPAYVKRCV
HLVSGHEVRGSFPLDLATGNGIEVVYPGVHSDVGGGYPPGEQGRGCATGPTL
KANDSEKLSQVPLCHMFREAVVAGVPLNPQTPGVTQEMRDAFKVAPALREVF
NAYVTATRPLLGDRISTTAAMQTHYGLYLRWRRLRLGPTGAEALEAQDFLKR
AKQFKHGQDYTDLTLANAELRDEWKALKKDEDDIWYEETWLGAILRGGLIP
VYLADRVWGEKVRQWQDVRRHWNDTAPLPSAVVRLLDDYAHDSRAWFKP
MGQTNEATWRAEQADRMTRLEAKDAASKEWERKNRPLLDAAQEAARKGRP
VYAPMLSPMPPGLTKQELADLTAYRKDKTLPTEPGPREYSSMWGYLRWRTLY
RNGVALTNKERLDLFISAQPPKEPPPMDAEGMRQIMSTKGIGDIFSGMR
>WP_104566914.1 *Ralstonia mannitolilytica*
MTELERLEPNPFALTVDEKGRCTAGNVGAFSGQCTRDFRIAFFFDGTNNNKDR
DTPKQAHSNVARLYDIFEVNPDQVRFYVPGIGTQFQKEIGDTGRGYDARAGL
GSGWGGEARINWALLKLTDALYGRYLGDDLSKAMGTDDLKLIKKISTDVNLP
VHLRASAGSDEVDELNNAGTFGTLAGTLDTATLAPNHQARRQELARRREFVE
QKLKPLIDGRKPTLNKIRLYVFGFSRGAAEARVFCSWLEDALGKDMTLAGVK
VSVDFLGIFDTVASVGFAQSFMIASGHGGWGNEEFLRIPGYVERTVHLISSHEI
RGSFPLDKAESKNCLELAFPGVHTDVGGAYQPGDQGRGCRADGTPDDSMKM
SQVTLAKMYREAVAAGVPLKPSTDLPTRVLDALKISPQLIKDYNDYVDAVNPL
IRKHGGGTTGAARVQYGLYLRWRRLRLNAGAQAFENQPFFKRAQKFSAQCA
QDLAGANEELREEAKIMAEMESSPVYSDGWMGKVFRNVPGPGGVVAEIQQF
VWGEKVKQWREVKSYWNDTSPLDARIVKFFDDYVHDSRAWFKPTGATNEE
VWKRRQRERLEDLKRKDAEWKAQWAEINAASARDPEGVRKRFAPVDEGGQ
GDIYPPLLVGQDRTDLDRYLKHGSLPLEKTGREFSSVWGYLRWRTHFAPTLPV
RERAQAAWTKASQIPGKVADKITDKAKDVAHSAEEKAAEAAGKILEAGKESL
IRGAKDMLDRFTGGGIPRL
>WP_247319026.1 *Ralstonia pseudosolanacearum*
MEQFEFHCSTEFHLSFFFDGTNNNRYRDTPRQAHSNVARLFDIFEEQEHQIRIY
VPGIGTPFEKEIGDTGRGDHARAGLGAGWGGEARINWALLQVTNALYAYYYT
QSLSMAMGVDELSLVHQTSSDLNMGLGAVSTAGRDEVEQLANAKNSEMVIT
AAETVLKPPRHEERRALLRQRREYLSEKLKALIAGRKPKMLRIRLSVFGFSRG
AAEARVFANWLKDALDDDMTLAGVPVSFDFLGIFDTVASVGVANSTKVATGH
SGWGEEAFLRIPGYVKRTVHLVSAHEVRGSFPLDKAVADNCLELAYPGVHTD
VGGAYQPGDQGRGCGTDGKPDDSNKLSQITLAKMYREAAAAGVPLNPMAR
NLTPEVRAALKISPDLIQAYNDYVDAVNPLIRKHGGGTTGAARVQYGLYLRW
RRMRLAGGAQAFENQPFFQRAEQYGAQCANDLSVANALLREEAKDLAAREN
DPAYADGWMQRVQRVLPVTQGVAVWNGVKQKVWGDKVREWREVKAYWN
DTAPLDPRIVRICDDYIHDSRAWFKPFGAPSDSVWRMRQQARLEQLRQQDAA
WKQVAADLSRDLIGTLKRYESTANGGDGEAAPNPVVGQDRRDLEQYLKDGS
VPMETKGREPSSMWGYLRWRTHFAPTPTLGERAQAAWDEVASVPGKVAKKA
QEAAHDAEARLANTARKLLEAGQESLERAATDALQRFLGGGVPRF
>RYO78518.1 *Monosporascus cannonballus*
MATRFQIQRRRSDEPAWVNDFPDSALAQLFGALPEANVYALVDNAFDTGFAQ

RLRSRFPGLHPQSLYEGRYDGPGLAEIAPSVVRIPVEESERRTFLEFVLNETSGK
PMLSFLHRIASALDPVAHLQDQMEAVDHEGKAFLIRFADTRSLDALLQVFDD
AQRERFLNGLRWWYFRRDGRLQAVGHPDDASADRTDEPYVFSREQMDRLD
ALARPDGLLRLIQTNVHWWGELTGTPSQAHSCIRMALDSPEFNAGAHDAVVF
RVVAVMKIRTWMALVLVVLATLSAGCKPAEQANAAQQDEDLGLEVRVLNYM
DEGLDIVYVNGVWVGSEKRHAGGFGVAGAIGVPRKWHPGLTVEVEWQDDT
LYRKDHDATYKAQVPVEPYPDGDPSSLWLAFYPGKKIRAIASRYTPLNPKFPG
GLKSDEMAELERLQPNPFALTLDEKGRCMANNIEAFSGQCTEELRIGIFFDGT
NNNKDRDAPKHAHSNVARLYDIFERKKNQVAFYVPGIGTPFEKEIGDTGRGY
DARAGLMAGWGGEARINWALLKLTDALYQLFYGDDLSRAMGTEDLKVLRKI
SGDLSFPVHKRAEAGARDEAQKIKDEVEQLNDAGTGRTLAVALDTADNIPNAI
PRKQELQRRREFLSQKLKALIADRKPKLQRIRLYVFGFSRGAAEARVFSNWLE
DAFDPGMTLAGVEVSFDFLGILDTVASVGFAQSSMFSTGHGGWGQEEFLRIPK
YVNRTVHLISSHEIRGSFPLDKAVARDLLEIAYPGVHTDVGGAYRPGDQGRGC
RADGTPDDSTKLSQVTLAKMYREAVAAGVPLKPSADLPPRVLDALKISPQLIK
DYNAYVDAVNPLIKKKGGGTTGAAHVQYGLYLRWRRLRLGSGPDAFENQPF
FRRAQNYSAQCAQDLSGANEELREEAKTMSEMENSILYSDGWMAAVFRRSG
VAAEALIRLHRAIWGDKVAQWREVKEYWNDTSPLDPRIVKFFDDYVHDSRA
WFKPMGATNEGVWKLREKNRLEDLKRKDAEWNARWKAEWQKMSADLQR
DPRGTIERYAPVSDGGKGEVAPPPPPVMGQDRIDLDRYLKDGTLPLEKTGREF
SSVWGYLRWRTHFAPELTLGERAQAAWDRVAAVPGKVADTVSQKVGEVAHS
AEEGLASTARKLLSAGQDALERGAKRVLEQYSGGGVPML
>WP_101490920.1 *Variovorax* sp. RO1
MTTSKFLEPLGEDPLALTKTQKALCLARHSSTAGLPCNKELHFGFFFDGTNNN
RIRDTPTKSQSNVARLYDVFDDAPAENARIYAAGVGTPFREEVGDSGLGAAAN
AGLGAGWGGEARINWALLQLHDSLYRYFFRGSNFSASLGTSDLATVKKMSA
DVNMSPGAIRNLGKSEVQMLREVGLTEKVGNIFDTTVTPVRDKARRDILRER
RQQLWNKLGSLVQQRKPKLERLCISVFGFSRGAAEARAFCNWLTDALDPDYT
LAGLKVEVVFLGIFDTVASVGMAQSALIRDGHDAWGRPKDLAIPGYVQRCVH
LVSGHEVRGSFPLDLAGGDGIEVVYPGVHSDVGGGYPPGEQGRGSANGTSLK
PNDSAKLSQAPLCQMFREAVTAGVPLDLNGPRVLQYMRDAFTVAPALREAFN
AYVAAVKPLLGDGISTPAAMQTHYGLYVRWRRLRLGPTGAEALETQDFLKRA
KQFKYGQDYTDLTLANAELRDEWKTLKKDEKDPLYDDTLLGTFLRGSSIPRF
LTDWVWGEKVRQWKDVRRHWNSPDPLPSAVIHLLDDYAHDSRAWFKPTGET
NESTWRAKQTDRMAQLEAKDAANKEGERRNRAILDTAQEAARRGRPVYAPT
ISPPPPALTKQELADLTAYRKDKTLPTETGSREPSSIWGYLRWRTLYRNGVALT
NKERLDSYISAQPPKEPPPMDAEGMRQIMSTKGIGDIFSGMR
>WP_198789541.1 *Variovorax* sp. IB41
MTTSKVLEPIGEDPLALTNAQKALCLAQQSSTASLPCSKELHYSFFFDGTNNN
RLRDTPNQSQSNVARLHDVFDANPAEHRRIYVAGVGTPFLAEVGDSGLGAAA
NAGLGAGWGGEARINWALLQLHDSLYRHFFGANFSASLGTSDIATVKKMSA
DINMSPGAIRNLGKSEVQMLRDVGLTDKLGNIFDTTVTPVRDTVRGDVLRER
RQQLWSKLSSLVQRKPKLEKLKISVFGFSRGAAEARAFCNWLNDAVDPDFTL
AGLKVEIVFLGIFDTVASVGLAQSALIRDGHDGWGRPKDLAVPSYVKRCVHL

VSGHEVRGSFPLDLAGGDGIEVVYPGVHSDVGGGYPPGEQGRGCATGPTLKA
NDNAKLSQAPLCHMFREAVTAGVPLNPNAPGVTQEMRDAFKVAPALRDAFN
AYVSAVKPLLGDGISTPAAMQTHYGLYVRWRRLRLGPTGAEALETQDFLKRA
KQFKYGQDYTDLTLANAELRDEWKAVKKDENNPLYEDTWLGKLLRNSSVST
YLTDWVWGEKVRQWNDVRRHWNSPDPLPSAVVRLLDDYAHDSRAWFKPTG
ETNESTWRAKETERMTQLEAKDAANKEGERKNREILNTLQEEARKGRPVYA
PMLSPPPPALTKQELADLTAYRKDKTLPTETGSREPSSIWGYLRWRTLYRNGVA
LTNKERLDAYISAQPPMEPPPMDAEGMRQIMSTKGIGDIFSGMR
>WP_118891105.1 *Ralstonia solanacearum*
MAELEPLGPNPFAPTVDEQARYLGCRMEGFETHCSTEFHLSFFFDGTNNNKY
RDTPKQAHSNVARLYDIFDANKERVRIYVPGIGTPFETEVGDTGRGNHARAGL
GTGWGGEARINWALLQVTNELHRLYYGVTLSDSLGFNERDLVRKISADLNIG
VAKMIAAGGDEVDQLAKASSVDVVAAAASAELFPPRHLQRRALLRQRREFLS
QKLKPVIDGRKPTLLRIRLSVFGFSRGAAEARVFSNWLKDALDDDMTLAGVE
VSFDFLGIFDTVATVGVANSTNVASGHSGWGEEEFLRIPKYVKRTVHLVSAHE
VRGSFPLDKAVADTCLELAYPGVHTDVGGAYQPGDQGRGCNAKGEPDDSNK
LSQLTLAKMYREAVAAGVPLNPQGRNVTERIKAALKISPDLIKAFNDYVDVV
NPLIRKRGGGTTGSVHVQYGLYLRWRRLRLASGAQAFENQPFFKRAQQFGA
QCAKDLSVAHEAIREESQDLATRENDPSYSDGWMRRALQVAPAGWLIAFGDG
FKRRMWGEKVKEWHEVKTYWNDTSPLDHRLVRVFDDYVHDSRAWFKPFGA
PSDAVWKQRQKVRLELLKQQDAAWKQVAADLNRDLLGTLKRFDTVQNGGQ
GEIAPLPVAGQDRADLDRYLKDGSLPLETEGREPSSMWGYLRWRTHFAPAPTF
GERAQAAWDRVAEVPGKVAHKVTEKVGEVVHNAEDSLANTARKLLSAGQES
LERSAKNMLEQYLGGGGAPRL
>RYP59163.1 *Monosporascus* sp. 5C6A
MPRWHSIRRPARRNPARAFLVSKKQKAPSNAARRLAICSKQHARTVTLASSAP
ATHAGPGARPAIVVGPDGQTQAGGDGEHHASPAGEIRVRFPWQQGERADDRS
SRWVRVAQRQAGSGMGWQWLPRIGQEVLVKFSEDDVDQPVVIGALYNGQGE
AGVAPTPGGQTAAQMDTAALYKQGSDTAPSAQGNVAGGHSPAWHGMGTDA
DGHRNAAAHTGFKSAEHGGSGYNQLVFDDSDGQLRTQLATTQLHSQINLGHL
VHQQDNRRGSFRGQGFELRTDGHAAVRGQAGLLLTTYRDAASGKAVPTGDN
AAGIALIKQAKALTQSLGQGAVTHQTAGLSSAKDENAPLAKQEKAAAGMVD
GKALDAAKQDASAGNTSTPGKVPHQGEAMVHANGRAGLVMVAGQDLQLA
NGESIALGSGQDTNIAVGKQARMHAGQAIGVAAGLSKAGDNNIGLQLTAGQG
NIDVQAQHDTLKLMAKDDLKLVSANMNVDFAAAKRIRLATAAGAAITLEDG
NITVECSGPITYKAAQRTFDGPVNQSYPLPAFPQSISDIEVIGTMATRFQIQRRR
SDEPAWVNDFPDSALAQLFGALPEANVYALVDNAFDTGFAQRLRSRFPGLHP
QSLYEGRYDGPGLAEIAPSVVRIPVEESERRTFLEFVLNETSGKPMLSFLHRIAS
ALDPVAHLQDQMEAVDHEGKAFLIRFADTRSLDALLQVFDDAQRERFLNGLR
WWYFRRDGRLQAVGHPDDASADRTDEPYVFSREQMDRLDALARPDGLLRLI
QTNVHWWGELTGTPSQAHSCIRMALDSPEFNAGAHDAVVFRVVAVMKIRTW
MALVLVVLATLSAGCKPAEQANAAQQDEDLGLEVRVLNYMDEGLDIVYVNG
VWVGSEKRHAGGFGVAGAIGVPRKWHPGLTVEVEWQDDTLYRKDHDATYK
AQVPVEPYPDGDPSSLWLAFYPGKKIRAIASRYTPLNPKFPGGLKSDEMAELE

RLQPNPFALTLDEKGRCMANNIEAFSGQCTEELRIGIFFDGTNNNKDRDAPKH
AHSNVARLYDIFERKKNQVAFYVPGIGTPFEKEIGDTGRGYDARAGLMAGWG
GEARINWALLKLTDALYQLFYGDDLSRAMGTEDLKVLRKISGDLSFPVHKRA
EAGARDEAQKIKDEVEQLNDAGTGRTLAVALDTADNIPNAIPRKQELQRRREF
LSQKLKALIADRKPKLQRIRLYVFGFSRGAAEARVFSNWLEDAFDPGMTLAG
VEVSFDFLGILDTVASVGFAQSSMFSTGHGGWGQEEFLRIPKYVNRTVHLISSH
EIRGSFPLDKAVARDLLEIAYPGVHTDVGGAYRPGDQGRGCRADGTPDDSTKL
SQVTLAKMYREAVAAGVPLKPSADLPPRVLDALKISPQLIKDYNAYVDAVNPL
IKKKGGGTTGAAHVQYGLYLRWRRLRLGSGPDAFENQPFFRRAQNYSAQCA
QDLSGANEELREEAKTMSEMENSILYSDGWMAAVFRRSGVAAEALIRLHRAI
WGDKVAQWREVKEYWNDTSPLDPRIVKFFDDYVHDSRAWFKPMGATNEGV
WKLREKNRLEDLKRKDAEWNARWKAEWQKMSADLQRDPRGTIERYAPVSD
GGKGEVAPPPPPVMGQDRIDLDRYLKDGTLPLEKTGREFSSVWGYLRWRTHF
APELTLGERAQAAWDRVAAVPGKVADTVSQKVGEVAHSAEEGLASTARKLLS
AGQDALERGAKRVLEQYSGGGVPML
>RZL61392.1 *Variovorax* sp
MARQRRPTMTTSKHLDPIGEDPLALTKTQKTLCLAHHAATANLPCAKELHFSF
FFDGTNNNRLRDTPTQSQSNVARLYDVFDANPAENRRIYAAGVGTEFQKEVG
DSGVGAAAAAGLGAGWGGEARINWALLQLHDLLYRHFFGANFSESLGTSDL
ATVKAMSADMNMSPAAVRKLGDQTELKMLRSVGLLTKLGDILNTSRISARDR
ERGDILRARRQVLWNKLSPLVLQRKPKLERLCISVFGFSRGAAEARAFCNWLT
DALDPDFTLAGLKVEVVFLGIFDTVASVGLAQSAMVSDGHYGWGRPKDLAV
PGYVQRCVHLVSGHEVRGSFPLDLAGGEGIEVVYPGVHSDVGGGYPPGEQGR
GCASGRSLRSNDSEKLSQVPLCQMFREAVTAGVPLDPNAPGVSKEMRDALKV
APTLRAAFNAYASTVNALLGHRISTPAAMQTHYGLYVRWRRLRLGPQGAQAL
ETQDFLERARQFQNGQDHTDLTQANAELREEWMAIQKDENNPAYDDTWVG
RALRGGDQLNALNPASVVFLPTKVRNWIFGEKIRQWRDVRGHWNNLAPLPS
SVTRLLDDYAHDSRAWFKPFGETNEATWRAKQVERMEALKQKRKVRQDWE
DRLNNPVDPTAMAPPGLTSQERADLAAYECSNEKKLPAELVGREPTSIWGYLR
WRTLYRDGIALTNTERLDAFIGAQPHTDPPRLSPLKPSSFAQ
